# Supplementary material for: Switchable bifunctional molecular recognition in water using a pH-responsive Endo-functionalized cavity
Source: Nat Commun. 2022 Apr 28;13:2291. doi: 10.1038/s41467-022-30012-4 (PMC9051166; doi:10.1038/s41467-022-30012-4)
Supplement: Supplementary file 1 — Supplementary Information [file 41467_2022_30012_MOESM1_ESM.pdf]

Supplementary Information

Switchable Bifunctional Molecular Recognition in Water Using a pH-Responsive *Endo*-Functionalized Cavity

Xiaoping Wang,[1] Mao Quan,[1] Huan Yao,[1] Xin-Yu Pang,[1] Hua Ke,[1] and Wei Jiang*[1]

[1] Shenzhen Grubbs Institute, Guangdong Provincial Key Laboratory of Catalysis, and Department of Chemistry, Southern University of Science and Technology, Xueyuan Blvd 1088, Shenzhen, 518055, China.

**E-mail:* jiangw@sustech.edu.cn

Table of Contents

1	Experimental Section	S2
2	pK_a Values and Critical Aggregation Concentration of **3**	S5
3	Host-Guest Binding Properties of **3b**	S7
4	Data of Configurational Assignments of **4** and **5**	S20
5	pK_a Values and Critical Aggregation Concentration of **5**	S29
6	^{1}H NMR Spectra of Host-Guest Complexes	S32
7	Determination of Association Constants by NMR Titrations	S46
8	Determination of Association Constants by ITC Titrations	S58
9	Association Constants of **5** with Tetramethylammonium	S65
10	Data for Switchable Bifunctional Molecular Recognition	S66
11	X-Ray Crystallography	S69
12	Synthetic Procedures	S73
13	Computational Data	S95
14	Supplementary References	S108

1. Experimental Section

1.1 General Method.

All the reagents and guest molecules involved in this research were commercially available and used without further purification unless otherwise noted. Solvents were either employed as purchased or dried prior to use by standard laboratory procedures. Thin-layer chromatography (TLC) was carried out on 0.25 mm Yantai silica gel plates (60F-254). Column chromatography was performed on silica gel 60 (Tsingdao 40 – 63 nm, 230 – 400 mesh). ^{1}H, ^{13}C NMR, 2D NMR spectra were recorded on a Bruker Avance-400 or 500 NMR spectrometer. Chemical shifts are reported in ppm with residual solvents as the internal standards. The following abbreviations were used for signal multiplicities: s, singlet; d, doublet; dd, doublet of doublet; m, multiplet. Electrospray-ionization high-resolution mass spectrometry (ESI-HRMS) experiments were conducted on an applied Q EXACTIVE mass spectrometry system. Fluorescence spectra (FL) were obtained on a Shimadzu RF-5301pc spectrometer. The synthesis of **S1**, **A1** and **A2** has been reported earlier.[1,2, 3]

1.2 pK_a Determiantion of Secondary Amine of Hosts

NMR method: 5 mL solution of host (2.0×10^{-4} M) was prepared in D_2O/H_2O = 1/9 and sodium methanesulphonate (1.0×10^{-4} M) was added as an internal standard. The pH value of the solution of host was adjusted from ca. 6.0 – 12.0 by adding NaOH (8 M) or HCl (2M) gradually. The real pH values of the solution were determined by a pH meter (Mettler Toledo, InLab® Expert Pro-ISM) at 25 ^{o}C. At each pH value, 0.5 mL solution was transferred to a NMR tube and ^{1}H NMR experiments were performed on a Bruker AV-500 NMR spectrometer at 25 ^{o}C. The ^{1}H NMR spectra and the chemical shifts of the corresponding protons are thus recorded.

For **5** with one secondary amine group, the data were then fit to the Henderson-Hasselbalch equation (Supplementary Equation 1) adapted for fast chemical exchange on the NMR time scale. The pK_a value and Hill coefficients are obtained from the curve fitting.

$$S = S_{(\mathrm{up})} + \frac{S_{(\mathrm{p})} - S_{(\mathrm{up})}}{1 + 10^{\mathrm{n}*(\mathrm{pH}-\mathrm{p}K_{\mathrm{a}})}} \quad \text{(Supplementary Equation 1)}$$

Where $S_{(\mathrm{up})}$ and $S_{(\mathrm{p})}$ are the chemical shift values (in ppm) or fluorescence intensity (a.u.) of the unprotonated and protonated states of secondary amine, respectively, and n is a Hill constant reflecting the number of proton binding sites.

Fluorescence method: a solution of host (5.0×10^{-6} M) was prepared in water (deionized water). The procedures of adjusting and determining the pH value are similar as the NMR

method. At each pH value, 2.5 mL solution was transferred to cuvette. The samples were excited at 330 nm and the emission spectra were collected from 350 to 600 nm. All fluorescence experiments were conducted at room temperature (~25 ^{o}C) on a Shimadzu RF-5301pc spectrometer.

For **5** with one secondary amine group, fluorescence intensity (a.u.) at 410 nm versus pH data were fit to a Henderson-Hasselbalch equation (Supplementary Equation 1) as in the NMR experiments.

For **3b** with two secondary amine groups, fluorescence intensity (a.u.) at 410 nm versus pH data were fit to equation (Supplementary Equation 2).

$$I = \frac{I_a * 10^{(2*\mathrm{pH}-\mathrm{p}K_{a1}-\mathrm{p}K_{a2})} + I_b * 10^{(\mathrm{pH}-\mathrm{p}K_{a1})} + I_c}{1 + 10^{(\mathrm{pH}-\mathrm{p}K_{a1})} + 10^{(2*\mathrm{pH}-\mathrm{p}K_{a1}-\mathrm{p}K_{a2})}} \quad \text{(Supplementary Equation 2)}$$

Where I_a, I_b and I_c are the fluorescence intensity (a.u.) of the doubly protonated state, mono-protonated state, and unprotonated state of both secondary amines, respectively.

1.3 Job Plot

5.0 × 10^{-4} M solution of host and guest were prepared in phosphate buffer (50 mM, pH = 7.4 or 12, H_2O/D_2O = 9/1), respectively. Next, 0.5 mL solution (V_{host} + V_{guest} = 0.5 mL) was added to 11 NMR tubes, respectively. And then, ^{1}H NMR experiments were performed on a Bruker AV-500 NMR spectrometer at 25 ^{o}C. The ^{1}H NMR spectra and the chemical shifts of the corresponding protons are thus recorded. Job plots were constructed from the chemical shift change (Δδ) of proton of host in ^{1}H NMR spectra by varying the ratio of host and guest with a fixed total volume (V_{host} + V_{guest} = 0.5 mL).

1.4 ^{1}H NMR Titrations

For **3b** with a critical aggregation concentration (CAC) of 0.16 mM at pH 7.4, a 1.0 × 10^{-4} M solution of host was prepared in phosphate buffer (50 mM, pH = 7.4 or 12, H_2O/D_2O = 9/1). For **5** with a higher CAC (> 0.8 mM) at pH7.4, a 2.0 × 10^{-4} M solution of host was prepared in phosphate buffer (50 mM, pH = 7.4 or 12, H_2O/D_2O = 9/1). This solution (0.5 mL) of host was placed in one NMR tube. The sample was then titrated with a solution of guest. For all the host-guest pairs, chemical exchange is fast on the NMR time scale. To determine the association constants, NMR titrations were performed by adding guest to the solution of host with a fixed concentration. Nonlinear curve-fitting method was then used to obtain the binding constants through the following Supplementary Equation 3:

$$\delta = \delta_0 + \Delta\delta\left(\frac{0.5}{[\mathbf{H}]_0}\right)\left(([\mathbf{G}] + [\mathbf{H}]_0 + \frac{1}{K_a} - \sqrt{\left([\mathbf{G}] + [\mathbf{H}]_0 + \frac{1}{K_a}\right)^2 - 4[\mathbf{H}]_0[\mathbf{G}]}\right) \quad \text{(Supplementary Equation 3)}$$

All of these ^{1}H NMR titrations experiments have been independently performed two or three times.

1.5 Switchable Bifunctional Molecular Recognition

0.1 M NaOH, 2 M NaOH, 0.1 M HCl and 2 M HCl were prepared in D_2O, respectively. 4.0 × 10^{-4} M solution of **5b** prepared in D_2O, which was adjusted to pH value pD 7.4 by adding 2 M NaOH and 2 M HCl. The real pH values of the solution of **5b** were determined by a pH meter (Mettler Toledo, InLab® Expert Pro-ISM) at 25 ^{o}C. 50 mM solution of **G4** and **G5** were prepared in D_2O, respectively. 500 uL solution of **5b** and 4 uL solution of **G4** or **G5** was transferred to a NMR tube and ^{1}H NMR experiments were performed on a Bruker AV-500 NMR spectrometer at 25 ^{o}C. The ^{1}H NMR spectra are recorded after gradually adding 0.1 M NaOH or 0.1 M HCl.

1.6 ITC Titrations

Isothermal titration calorimetry, ITC. Titration experiments were carried out in phosphate buffer (50 mM, pH = 7.4 or 12) at 298 K on a VP-ITC instrument. In a typical experiment, a 1.4338 mL solution of host was placed in the sample cell, and 292 μL of a solution of guest was in the injection syringe. Heats of dilution, measured by titration of guest into the sample cell with blank solvent, were subtracted from each data set. All solutions were degassed prior to titration. The data were analyzed using the instrumental internal software package and fitted by “one set of binding sites” model. All of these ITC titration experiments have been independently performed three times.

2. pK_a Values and Critical Aggregation Concentration of 3

2.1 pK_a values Determiantion of 3b by ^{1}H NMR and Fluorescence spectra

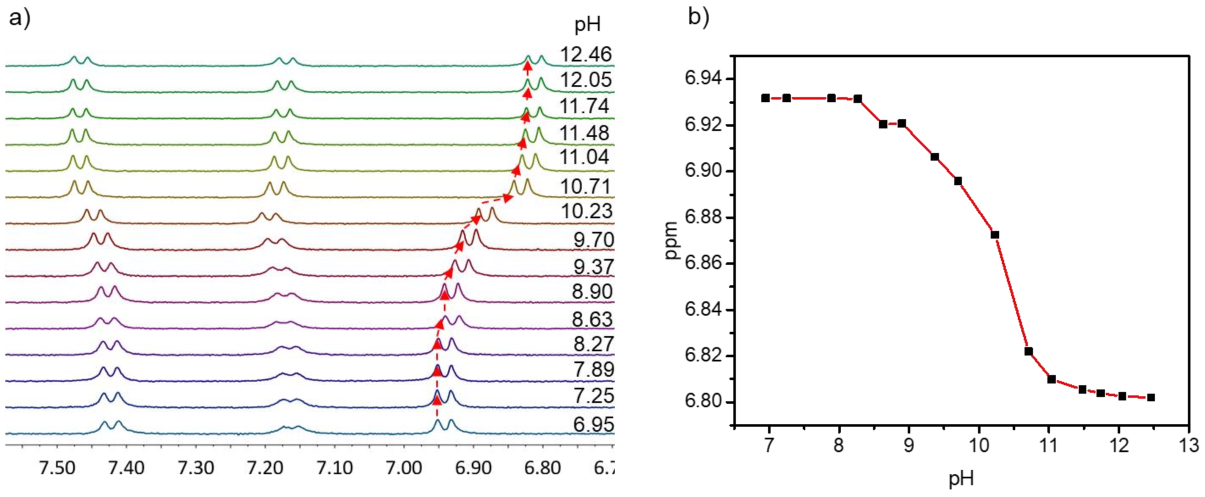

Supplementary Fig. 1 a) ^{1}H NMR chemical shift dependence on the ionization state of the **3b**. The proton signals of **3b** toward to up field with increasing the pH values. b) Titration curve tracking the change in chemical shift with pH for **3b**, which cannot be fitted may due to the barely noticeable two inflection points. However, the chemical shifts change was no longer present at pH < 8 or pH >12, which indicated that two secondary amine group of **3b** was full protonated at pH 8, and full deprotonated at pH 12.

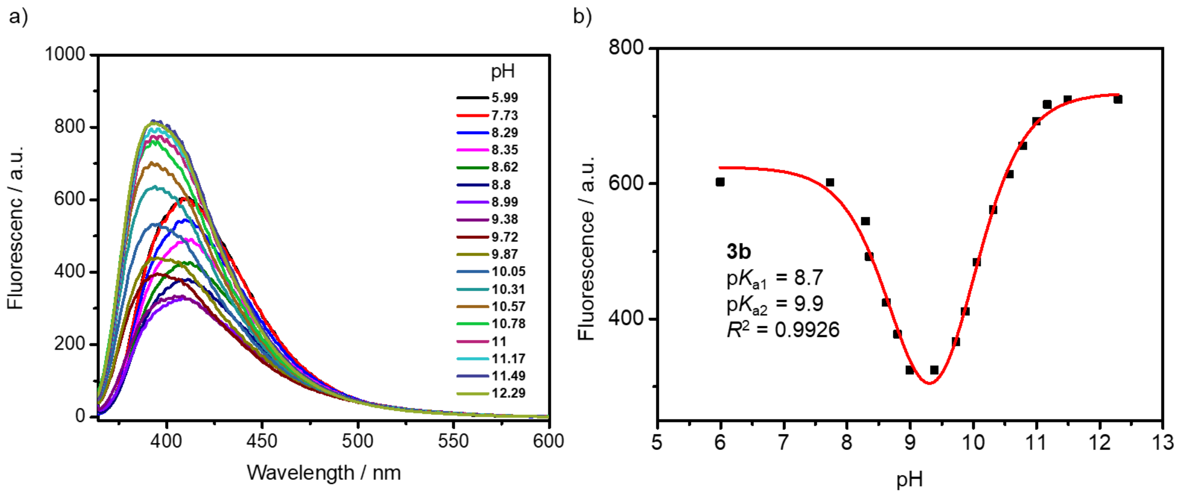

Supplementary Fig. 2 a) Fluorescence intensity dependence on the ionization state of the **3b**. The fluorescence intensity of **3b** changed with increasing pH. b) Titration curve tracking the change in fluorescence intensity with pH for **3b**, which was fit to Supplementary Equation 2 to determine pK_{a1} and pK_{a1} value. The pK_{a1} and pK_{a1} for this trial is 8.7 and 9.9, respectively.

2.2 Critical Aggregation Concentration of 3

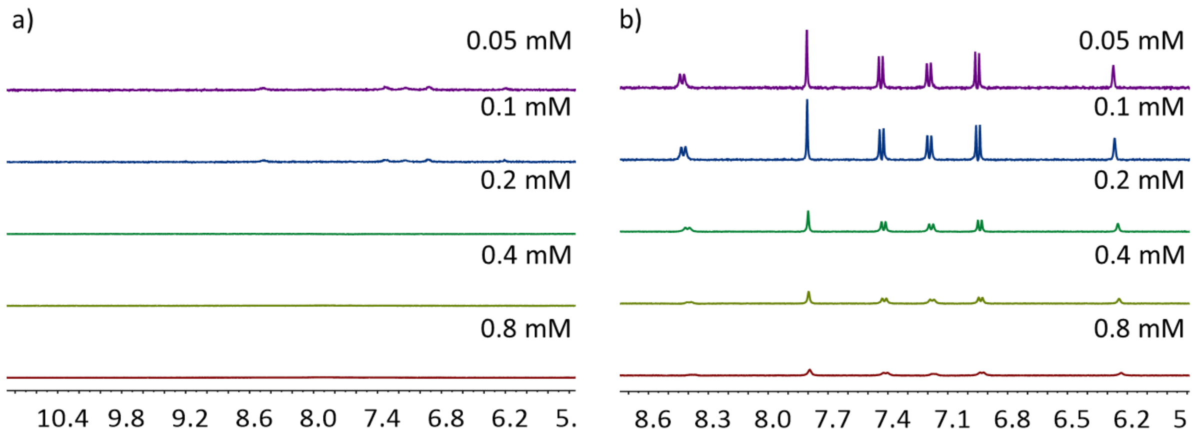

Supplementary Fig. 3 Partial ^{1}H NMR spectra (500 MHz, D_2O/H_2O = 1/9, 298 K) of (a) **3a** and (b) **3b** at different concentrations (0.8 mM – 0.05 mM) in phosphate buffer (50 mM, pH = 7.4). The peaks of **3a** almost disappear into the baseline, which indicates the severe aggregation at pH 7.4 (phosphate buffer, 50 mM). The peaks of **3b** disappear gradually into the baseline from the concentration of 0.2 mM, suggesting aggregation of the host. But the peaks remain sharp and similar below the concentration of 0.1 mM, suggesting the host exists in unimolecular form. Thus, the critical aggregation concentration of **3b** should be within 0.1 - 0.2 mM.

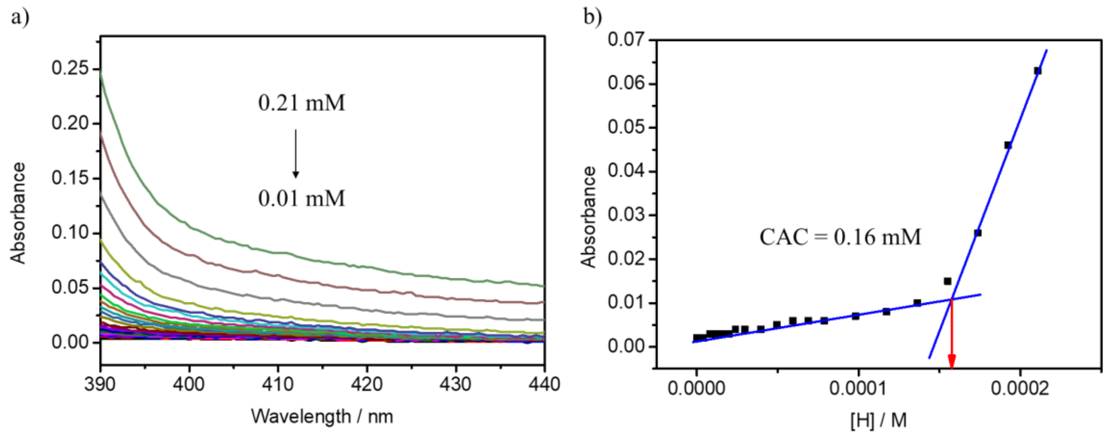

Supplementary Fig. 4 (a) UV–Vis spectra of the aqueous solution of **3b** at different concentrations (0.21 mM – 0.01 mM) in phosphate buffer (50 mM, pH = 7.4) at 298 K. (b) A plot of the absorbance against concentration of **3b** at 425 nm, respectively. The critical aggregation concentration of **3b** in pH 7.4 PB was determined to be 0.16 mM which is in line with the result from the NMR experiments.

3. Host-Guest Binding Properties of 3b

3.1 Association Constants of 3b with G1-G7

Supplementary Table 1. Association constants (K_a, M^{-1}) of naphthotubes **3b** with **G1** – **G7** in phosphate buffer (50 mM, pH = 7.4 or 12, H_2O/D_2O = 9/1) at 298 K as determined by ^1H NMR titrations. [a]

Guest	Host	K_a (M^{-1}) pH 7.4	K_a (M^{-1}) pH 12
**G1**	**3b**	$(3.8 \pm 0.1) \times 10^2$	$(3.9 \pm 0.7) \times 10^1$
**G2**	**3b**	- [b]	$(5.0 \pm 0.6) \times 10^2$
**G3**	**3b**	$(4.4 \pm 0.2) \times 10^2$	$(3.7 \pm 0.7) \times 10^1$
**G4**	**3b**	- [b]	$(7.0 \pm 0.2) \times 10^1$
**G5**	**3b**	$(8 \pm 1) \times 10^1$	- [b]
**G6**	**3b**	$(1.6 \pm 0.1) \times 10^1$	$(7 \pm 1) \times 10^2$
**G7**	**3b**	- [b]	$(1.7 \pm 0.2) \times 10^4$

[a] The data were averaged from two or three independent titrations. [b] The association constants could not be obtained due to severe aggregation in the host-guest mixture.

3.2 ^{1}H NMR Spectra of Host-Guest Complexes of 3b with G1-G7

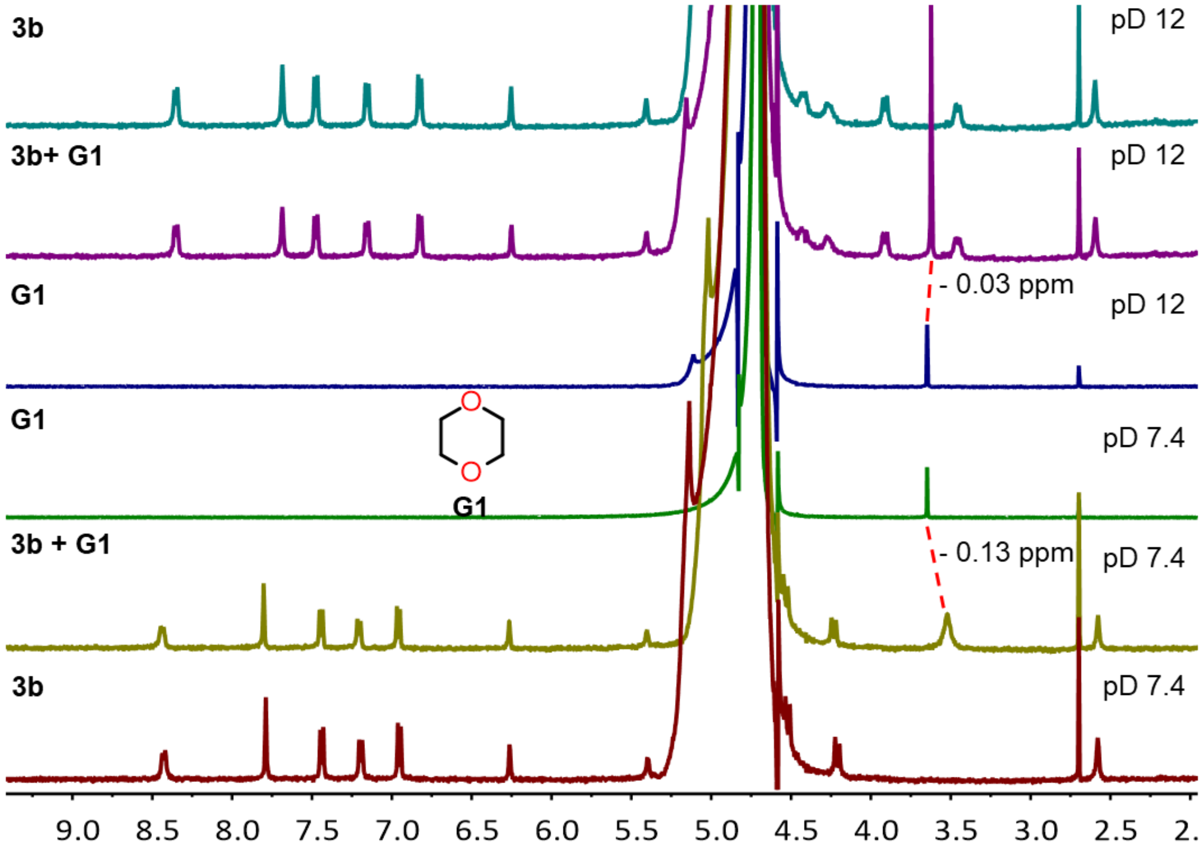

Supplementary Fig. 5 ^{1}H NMR spectra (500 MHz, D_2O, 0.1 mM, 298 K) of **G1**, **3b**, and their equimolar mixture in pD 7.4 and 12 (phosphate buffer, 50 mM), respectively. The protons of **G1** undergo more significant upfield shift in pD 7.4 than that in pD 12, suggesting that the binding behavior between **3b** and **G1** in pD 7.4 phosphate buffer is stronger than that in pD 12.

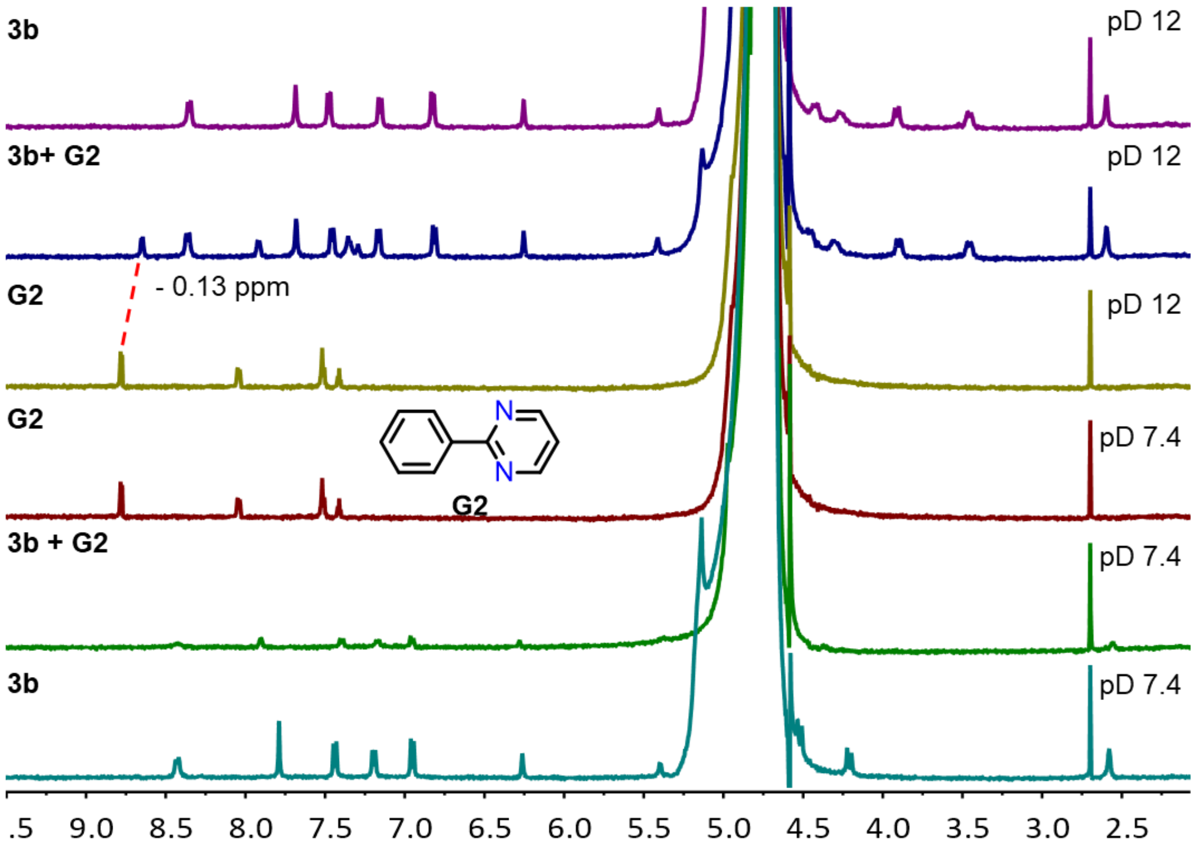

Supplementary Fig. 6 ^{1}H NMR spectra (500 MHz, D_2O, 0.1 mM, 298 K) of **G2**, **3b**, and their equimolar mixture in pD 7.4 and 12 (phosphate buffer, 50 mM), respectively. The peaks of **3b** and **G2** almost disappear into the baseline, which indicates the severe aggregation in the host-guest mixture at pD 7.4 (phosphate buffer, 50 mM). However, the protons of the guest **G2** undergo minor shifts, suggesting the weak interactions in the complex of **3b** and **G2** at pD 12 (phosphate buffer, 50 mM).

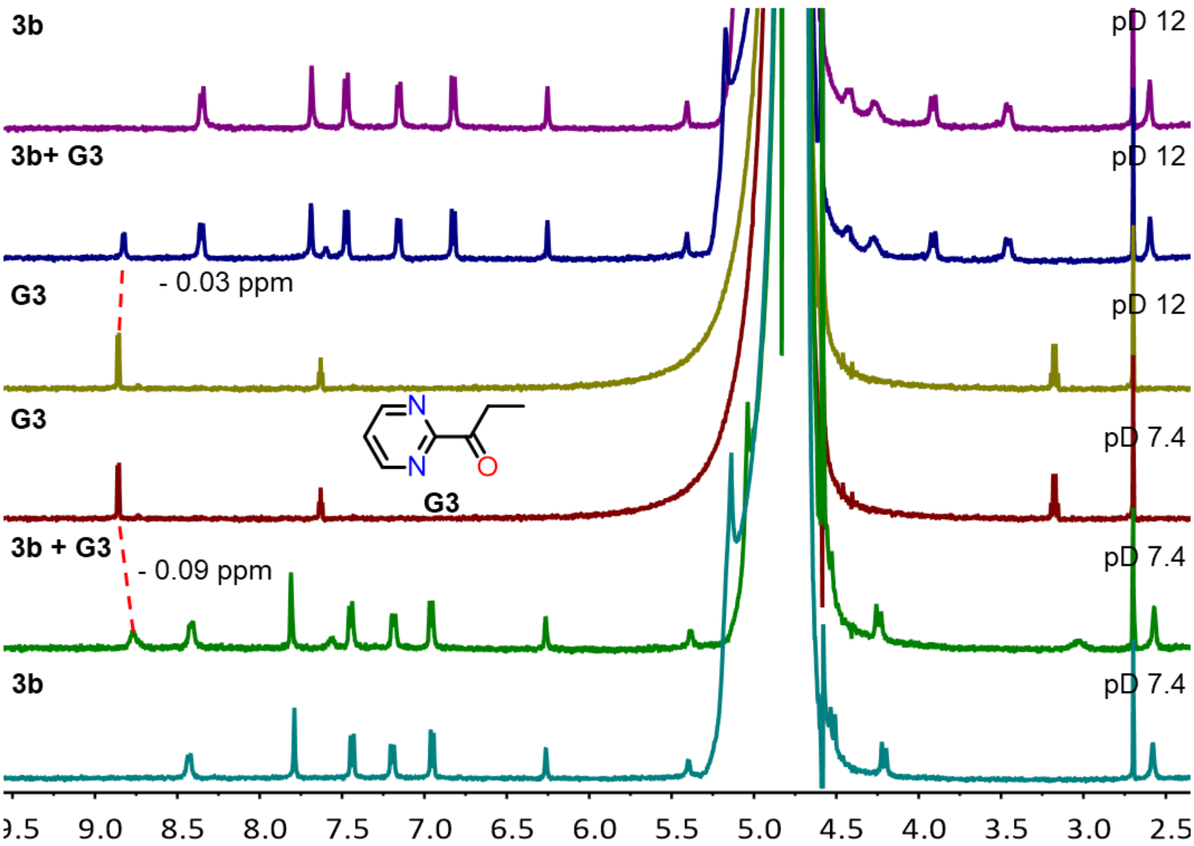

Supplementary Fig. 7 ^{1}H NMR spectra (500 MHz, D_2O, 0.1 mM, 298 K) of **G3**, **3b**, and their equimolar mixture in pD 7.4 and 12 (phosphate buffer, 50 mM), respectively. The protons of **G3** undergo more significant upfield shift in pD 7.4 than that in pD 12, suggesting that the binding behavior between **3b** and **G3** in pD 7.4 phosphate buffer is stronger than that in pD 12. The protons of the guest **G2** undergo minor shifts, suggesting the weak interactions in the complex of **3b** and **G2** at pD 7.4 and 12 (phosphate buffer, 50 mM), respectively.

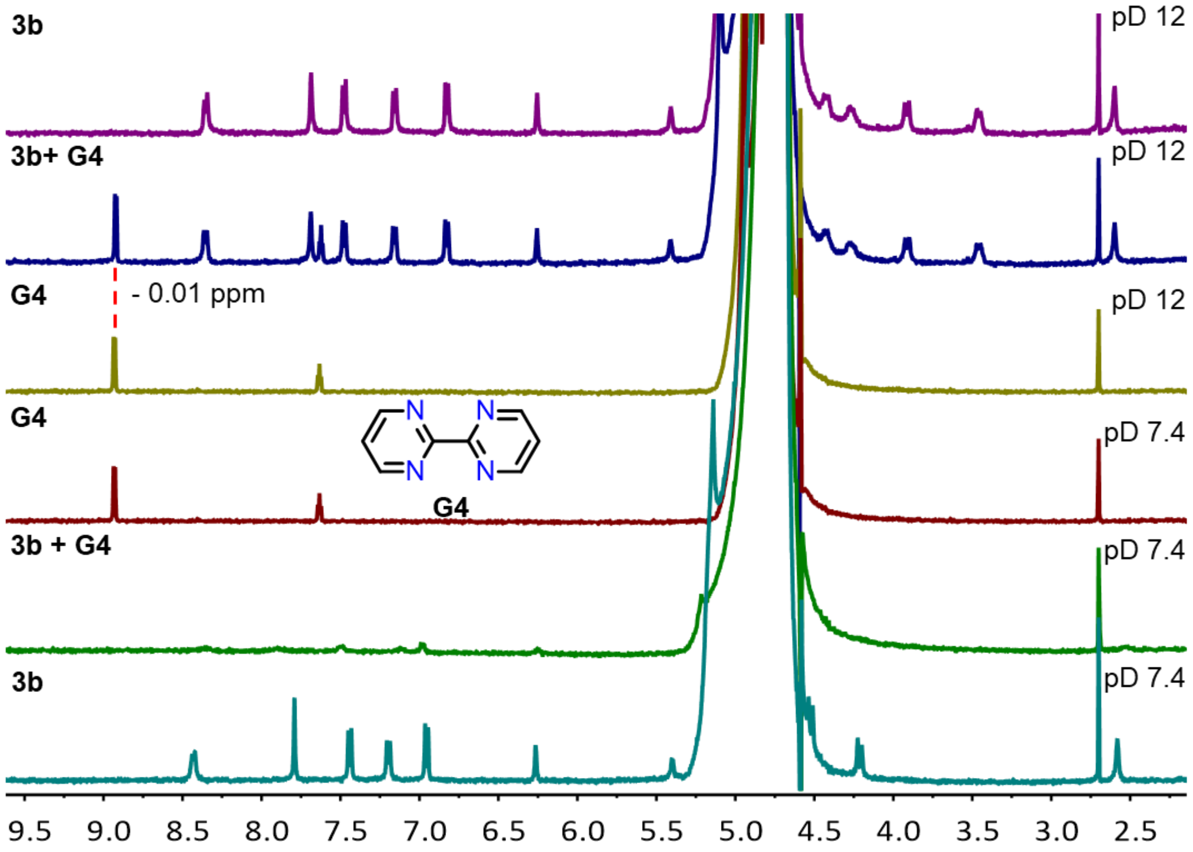

Supplementary Fig. 8 ^{1}H NMR spectra (500 MHz, D_2O, 0.1 mM, 298 K) of **G4**, **3b**, and their equimolar mixture in pD 7.4 and 12 (phosphate buffer, 50 mM), respectively. The peaks of **3b** and **G4** almost disappear into the baseline, which indicates the severe aggregation in the host-guest mixture at pD 7.4 (phosphate buffer, 50 mM). However, the protons of the guest **G4** undergo minor shifts, suggesting the weak interactions in the complex of **3b** and **G4** at pD 12 (phosphate buffer, 50 mM).

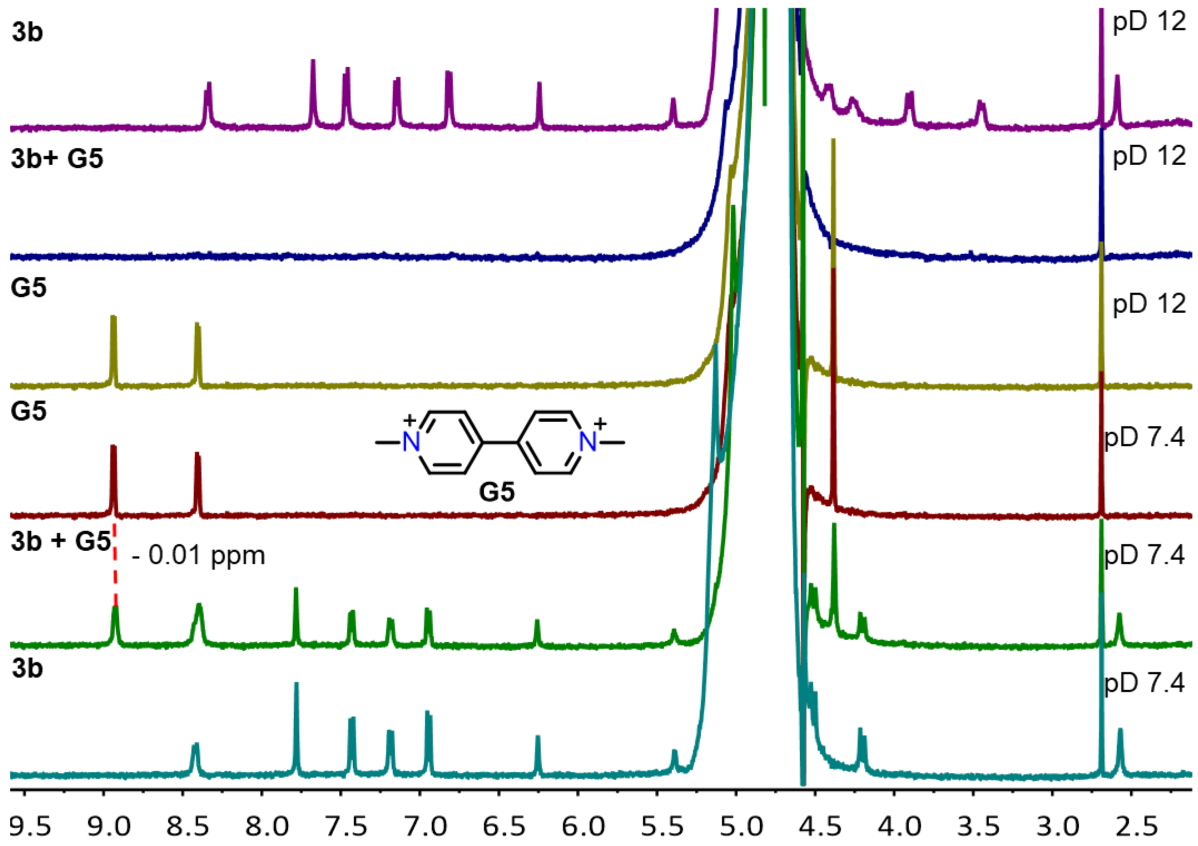

Supplementary Fig. 9 ^{1}H NMR spectra (500 MHz, D_2O, 0.1 mM, 298 K) of **G5**, **3b**, and their equimolar mixture in pD 7.4 and 12 (phosphate buffer, 50 mM), respectively. The peaks of **3b** and **G5** almost disappear into the baseline, which indicates the severe aggregation in the host-guest mixture at pD 12 (phosphate buffer, 50 mM). The protons of the guest **G5** undergo minor shifts, suggesting the weak interactions in the complex of **3b** and **G5** at pD 7.4 (phosphate buffer, 50 mM).

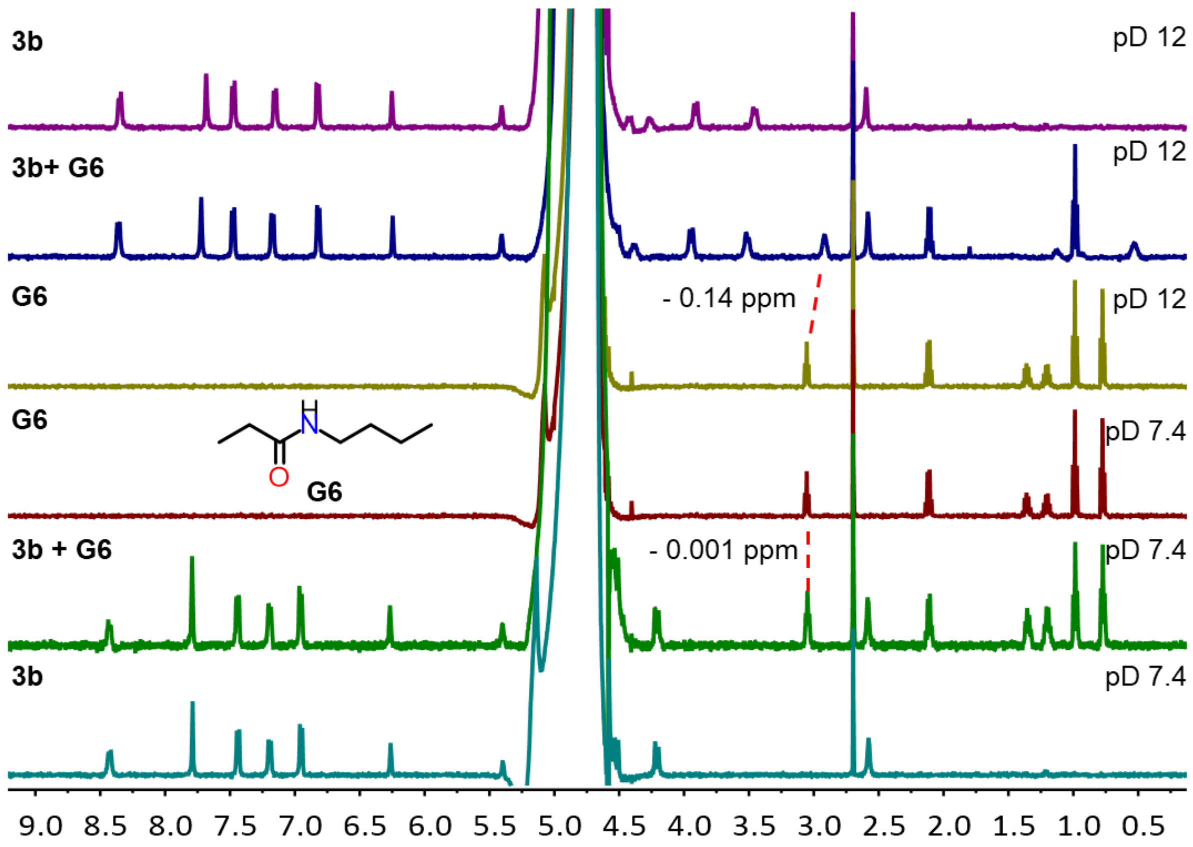

Supplementary Fig. 10 ^{1}H NMR spectra (500 MHz, D_2O, 0.1 mM, 298 K) of **G6**, **3b**, and their equimolar mixture in pD 7.4 and 12 (phosphate buffer, 50 mM), respectively. The protons of **G6** undergo more significant upfield shift in pD 12 than that in pD 7.4, suggesting that the binding behavior between **3b** and **G6** in pD 12 phosphate buffer is stronger than that in pD 7.4.

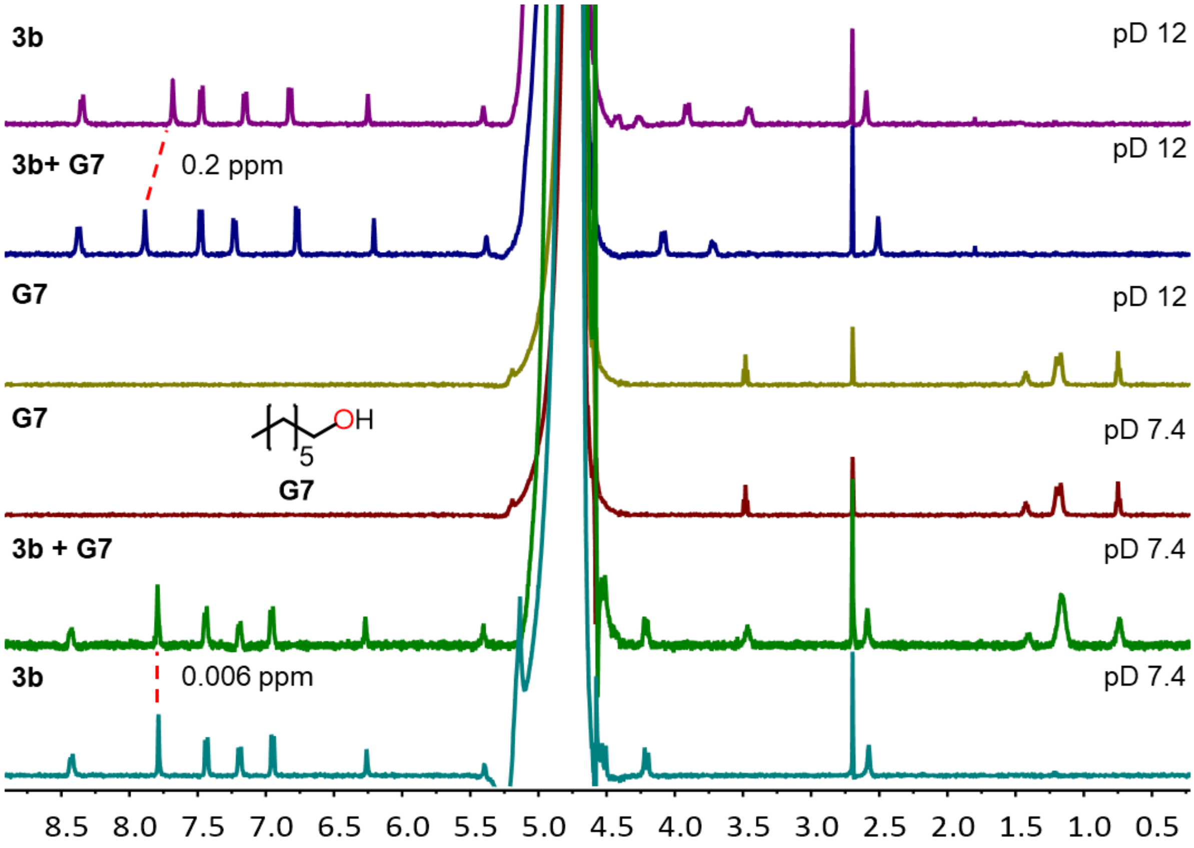

Supplementary Fig. 11 ^{1}H NMR spectra (500 MHz, D_2O, 0.1 mM, 298 K) of **G7**, **3b**, and their equimolar mixture in pD 7.4 and 12 (phosphate buffer, 50 mM), respectively. The protons of **G7** undergo more significant upfield shift in pD 12 than that in pD 7.4, suggesting that the binding behavior between **3b** and **G7** in pD 12 phosphate buffer is stronger than that in pD 7.4.

3.3 Determination of Association Constants by NMR Titrations

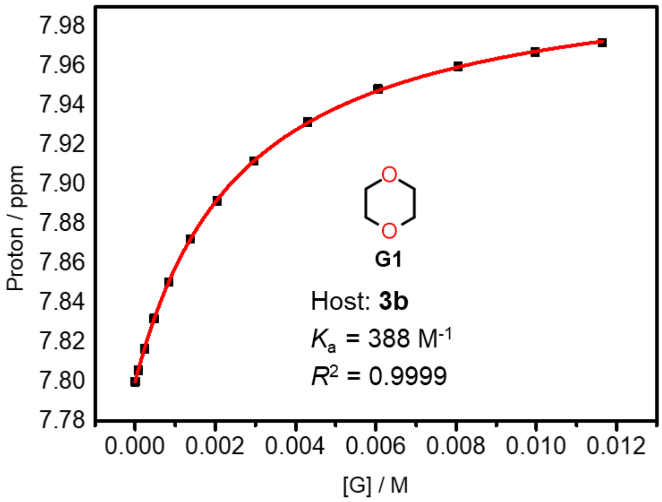

	K_a / M^{-1}	R^2
1st	388	0.9999
2nd	367	0.9991
average	$(3.8 \pm 0.1) \times 10^2$	

Supplementary Fig. 12 Non-linear curve-fitting for the complexation between **3b** and **G1** in phosphate buffer (50 mM, pH = 7.4, D_2O/H_2O = 1:9) at 298 K. K_a was given by averaging the values obtained from two independent titrations.

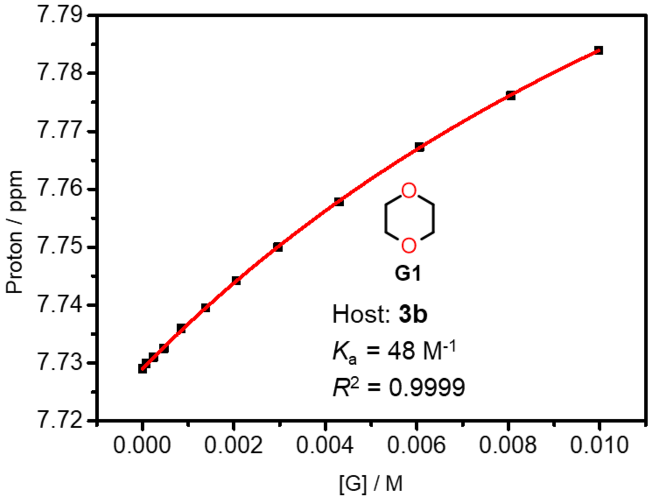

	K_a / M^{-1}	R^2
1st	48	0.9999
2nd	39	0.9998
3rd	30	0.9995
average	$(3.9 \pm 0.7) \times 10^1$	

Supplementary Fig. 13 Non-linear curve-fitting for the complexation between **3b** and **G1** in phosphate buffer (50 mM, pH = 12, D_2O/H_2O = 1:9) at 298 K. K_a was given by averaging the values obtained from three independent titrations.

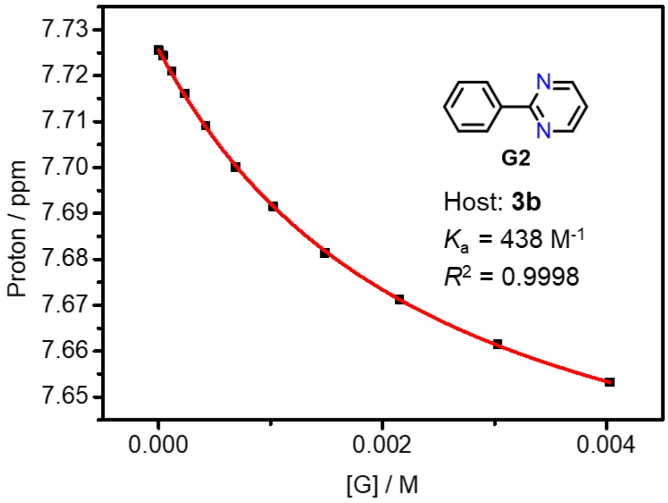

	K_a / M^{-1}	R^2
1st	438	0.9998
2nd	560	0.9971
average	$(5.0 \pm 0.6) \times 10^2$	

Supplementary Fig. 14 Non-linear curve-fitting for the complexation between **3b** and **G2** in phosphate buffer (50 mM, pH = 12, D_2O/H_2O = 1:9) at 298 K. K_a was given by averaging the values obtained from two independent titrations.

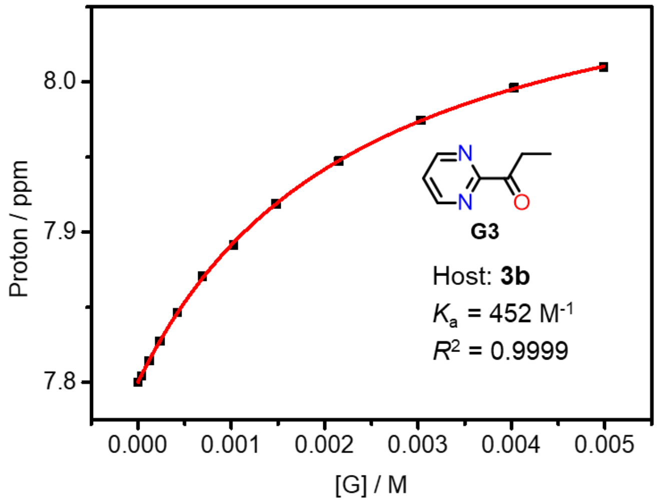

	K_a / M^{-1}	R^2
1st	452	0.9999
2nd	418	0.9999
average	$(4.4 \pm 0.2) \times 10^2$	

Supplementary Fig. 15 Non-linear curve-fitting for the complexation between **3b** and **G3** in phosphate buffer (50 mM, pH = 7.4, D_2O/H_2O = 1:9) at 298 K. K_a was given by averaging the values obtained from two independent titrations.

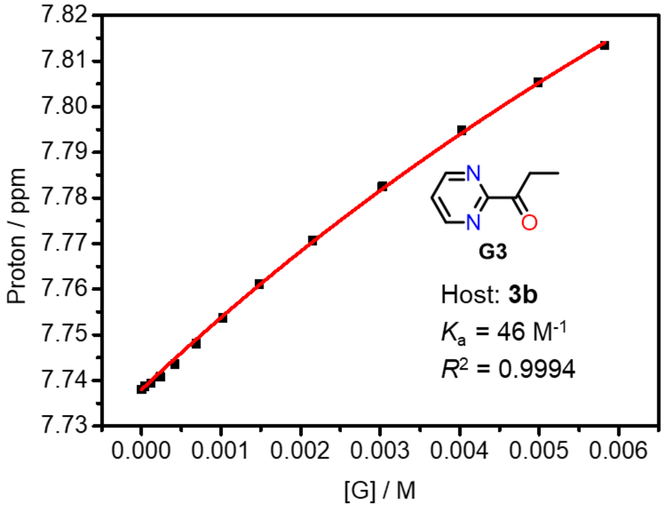

	K_a / M^{-1}	R^2
1st	46	0.9994
2nd	35	0.9998
3rd	30	0.9996
average	$(3.7 \pm 0.7) \times 10^1$	

Supplementary Fig. 16 Non-linear curve-fitting for the complexation between **3b** and **G3** in phosphate buffer (50 mM, pH = 12, D_2O/H_2O = 1:9) at 298 K. K_a was given by averaging the values obtained from three independent titrations.

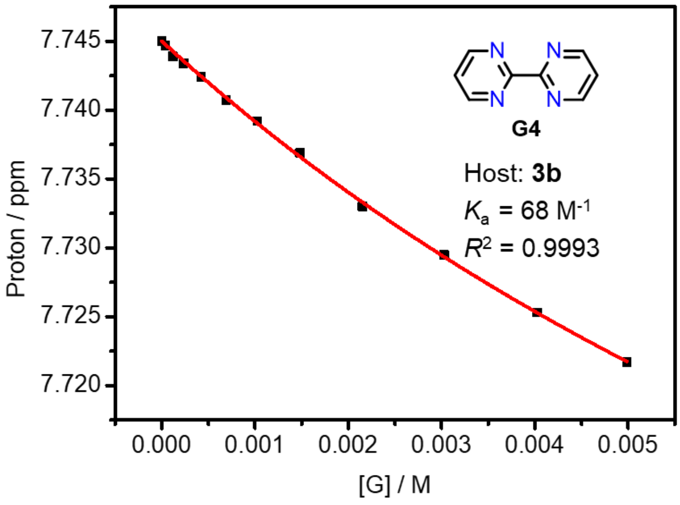

	K_a / M^{-1}	R^2
1st	68	0.9993
2nd	72	0.9994
average	$(7.0 \pm 0.2) \times 10^1$	

Supplementary Fig. 17 Non-linear curve-fitting for the complexation between **3b** and **G4** in phosphate buffer (50 mM, pH = 12, D_2O/H_2O = 1:9) at 298 K. K_a was given by averaging the values obtained from two independent titrations.

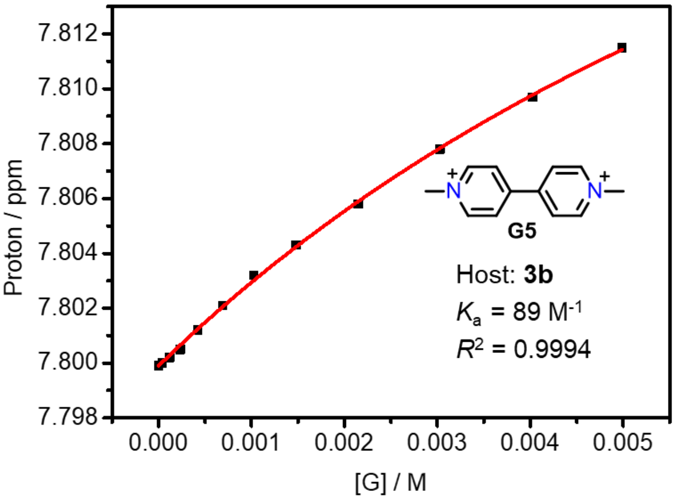

	K_a / M^{-1}	R^2
1st	89	0.9994
2nd	61	0.9994
average	$(8 \pm 1) \times 10^1$	

Supplementary Fig. 18 Non-linear curve-fitting for the complexation between **3b** and **G5** in phosphate buffer (50 mM, pH = 7.4, D_2O/H_2O = 1:9) at 298 K. K_a was given by averaging the values obtained from two independent titrations.

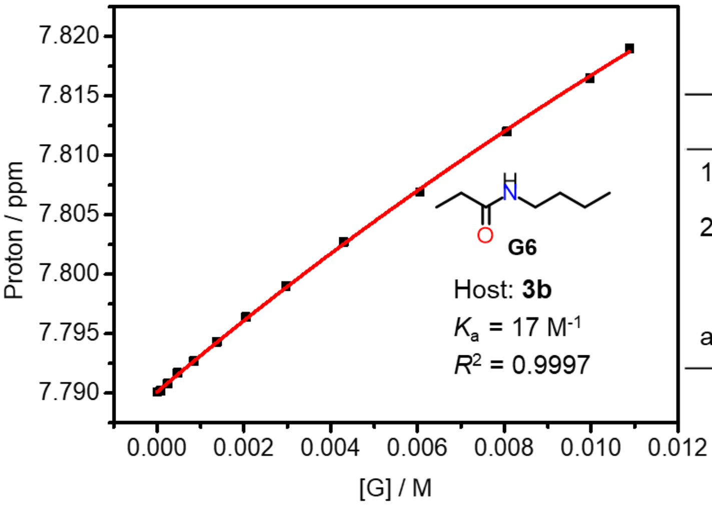

	K_a / M^{-1}	R^2
1st	17	0.9997
2nd	15	0.9997
average	$(1.6 \pm 0.1) \times 10^1$	

Supplementary Fig. 19 Non-linear curve-fitting for the complexation between **3b** and **G6** in phosphate buffer (50 mM, pH = 7.4, D_2O/H_2O = 1:9) at 298 K. K_a was given by averaging the values obtained from two independent titrations.

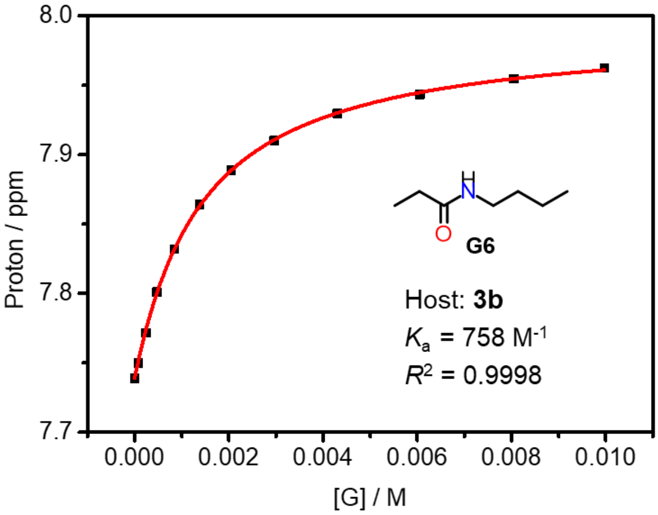

	K_a / M^{-1}	R^2
1st	758	0.9998
2nd	567	0.9990
3rd	893	0.9999
average	$(7 \pm 1) \times 10^2$	

Supplementary Fig. 20 Non-linear curve-fitting for the complexation between **3b** and **G6** in phosphate buffer (50 mM, pH = 12, D_2O/H_2O = 1:9) at 298 K. K_a was given by averaging the values obtained from three independent titrations.

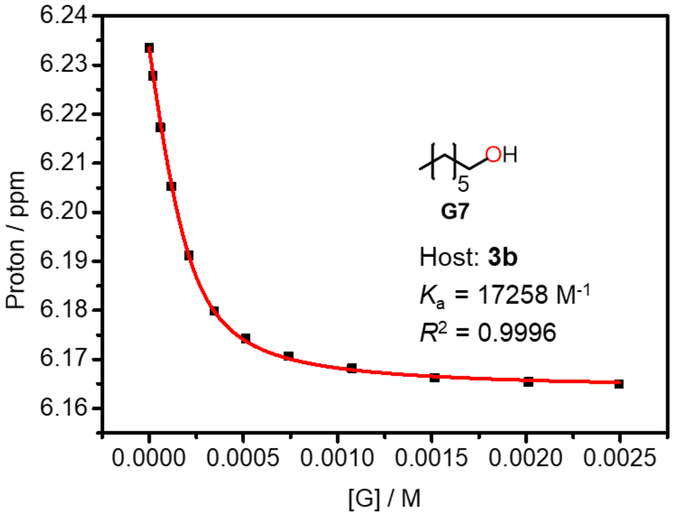

	K_a / M^{-1}	R^2
1st	17258	0.9996
2nd	19208	0.9992
3rd	14271	0.9996
average	$(1.7 \pm 0.2) \times 10^4$	

Supplementary Fig. 21 Non-linear curve-fitting for the complexation between **3b** and **G7** in phosphate buffer (50 mM, pH = 12, D_2O/H_2O = 1:9) at 298 K. K_a was given by averaging the values obtained from three independent titrations.

4. Data of Configurational Assignments of 4 and 5

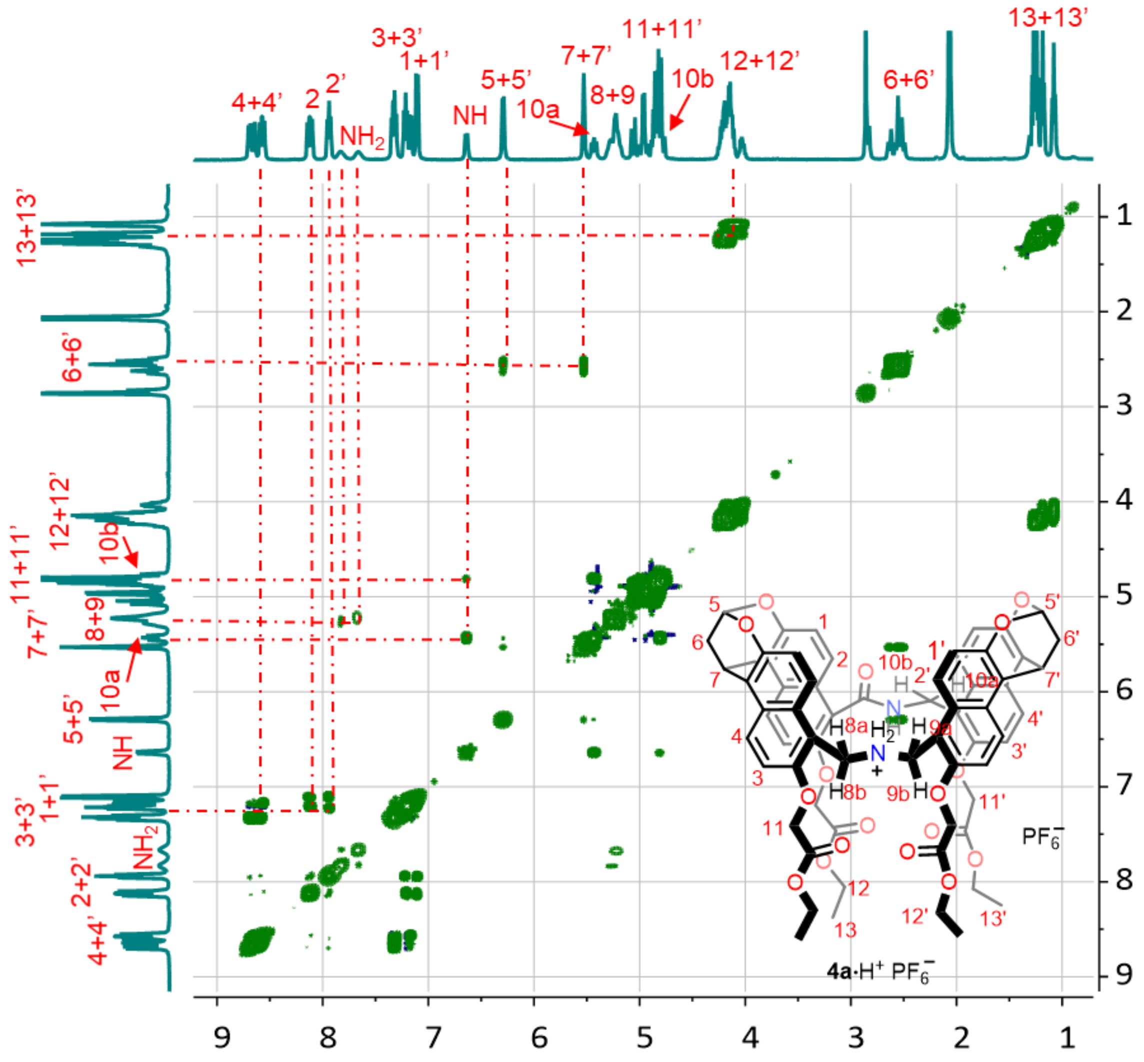

Supplementary Fig. 22 ^{1}H, ^{1}H-COSY NMR spectrum of **4a**·H^{+} PF$_6^-$ (500 MHz, acetone-d_6, 298 K).

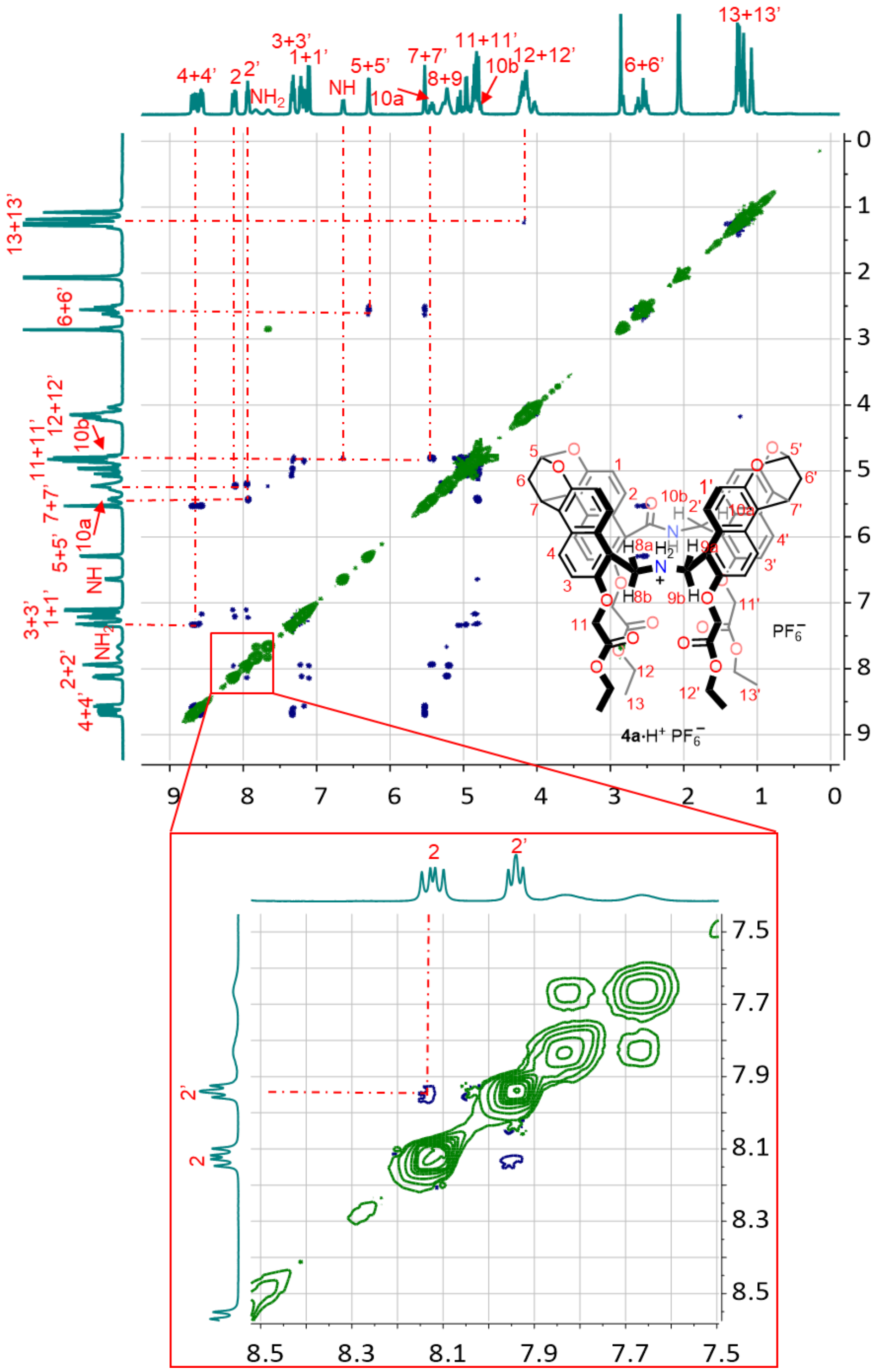

Supplementary Fig. 23 ^1H, ^1H-ROESY NMR spectrum of **4a**·H^+ PF_6^- (500 MHz, acetone-d_6, 298 K). NOE effect was detected between the protons 2 and protons 2'. This structure was assigned to the *syn* isomer, which was confirmed by the crystal structure.

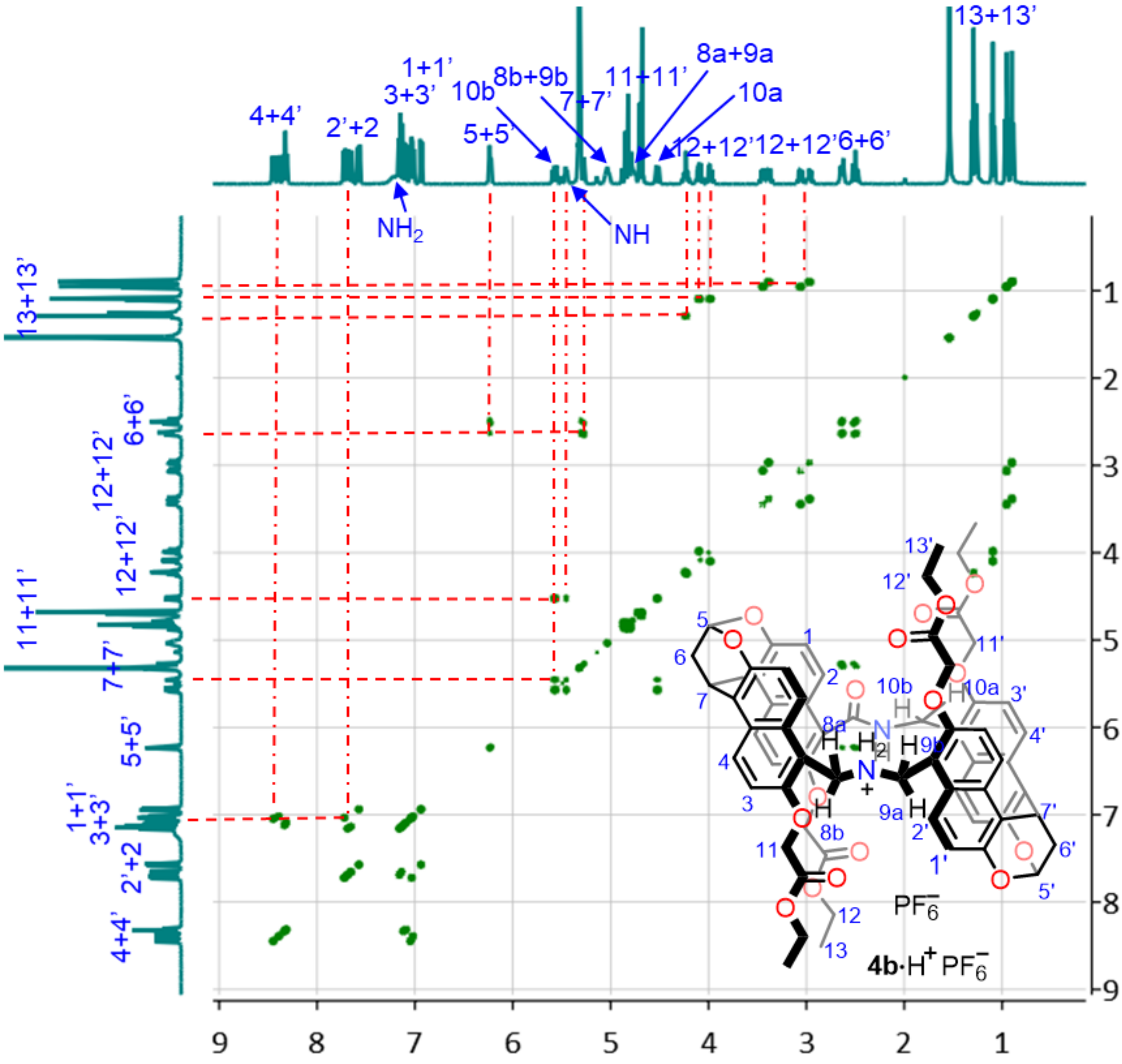

Supplementary Fig. 24 ^{1}H, ^{1}H-COSY NMR spectrum of **4b**·H^+ PF_6^- (500 MHz, CD_2Cl_2, 298 K).

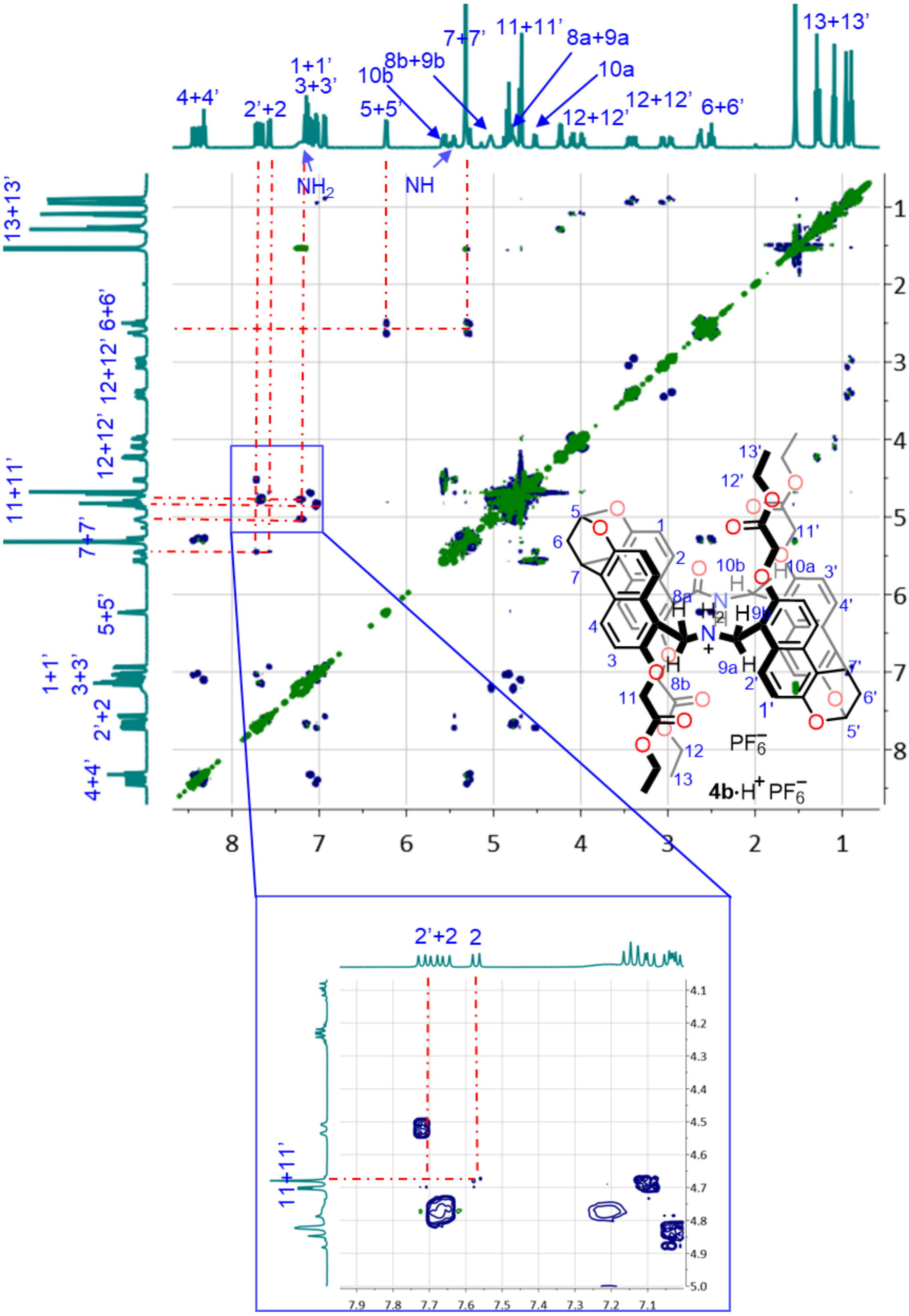

Supplementary Fig. 25 ^{1}H, ^{1}H-ROESY NMR spectrum of **4b**·H^{+} PF_6^- (500 MHz, CD_2Cl_2, 298 K). The protons (11+11') have NOE contacts with the aromatic protons (2 and 2'). Therefore, this structure was assigned to the *anti* isomer, which was confirmed by the crystal structure.

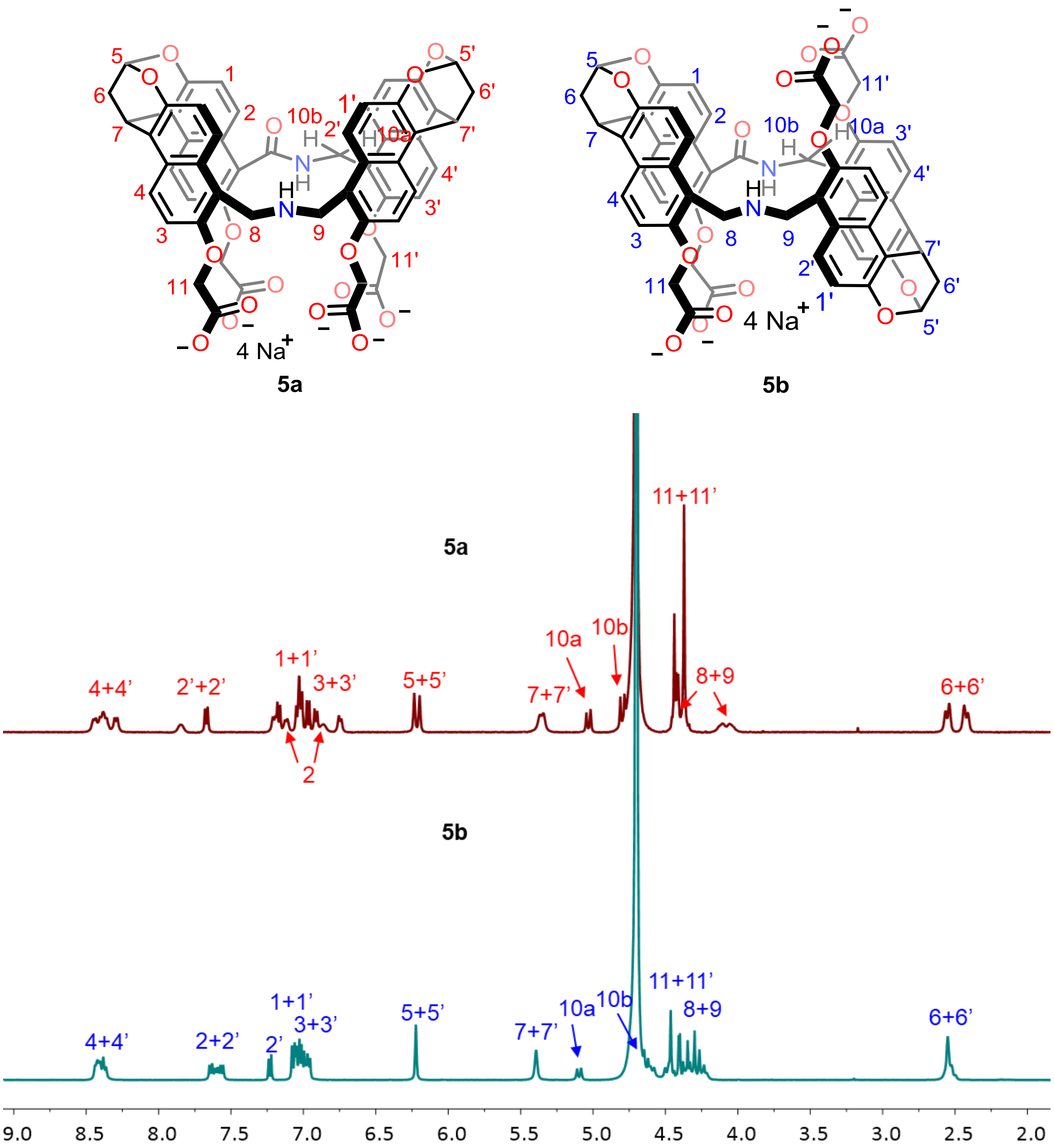

Supplementary Fig. 26 Chemical structures and ^{1}H NMR spectra (500 MHz, D_2O, 298 K) of **5a** and **5b**. Peaks were assigned according to the following 2D NMR spectra.

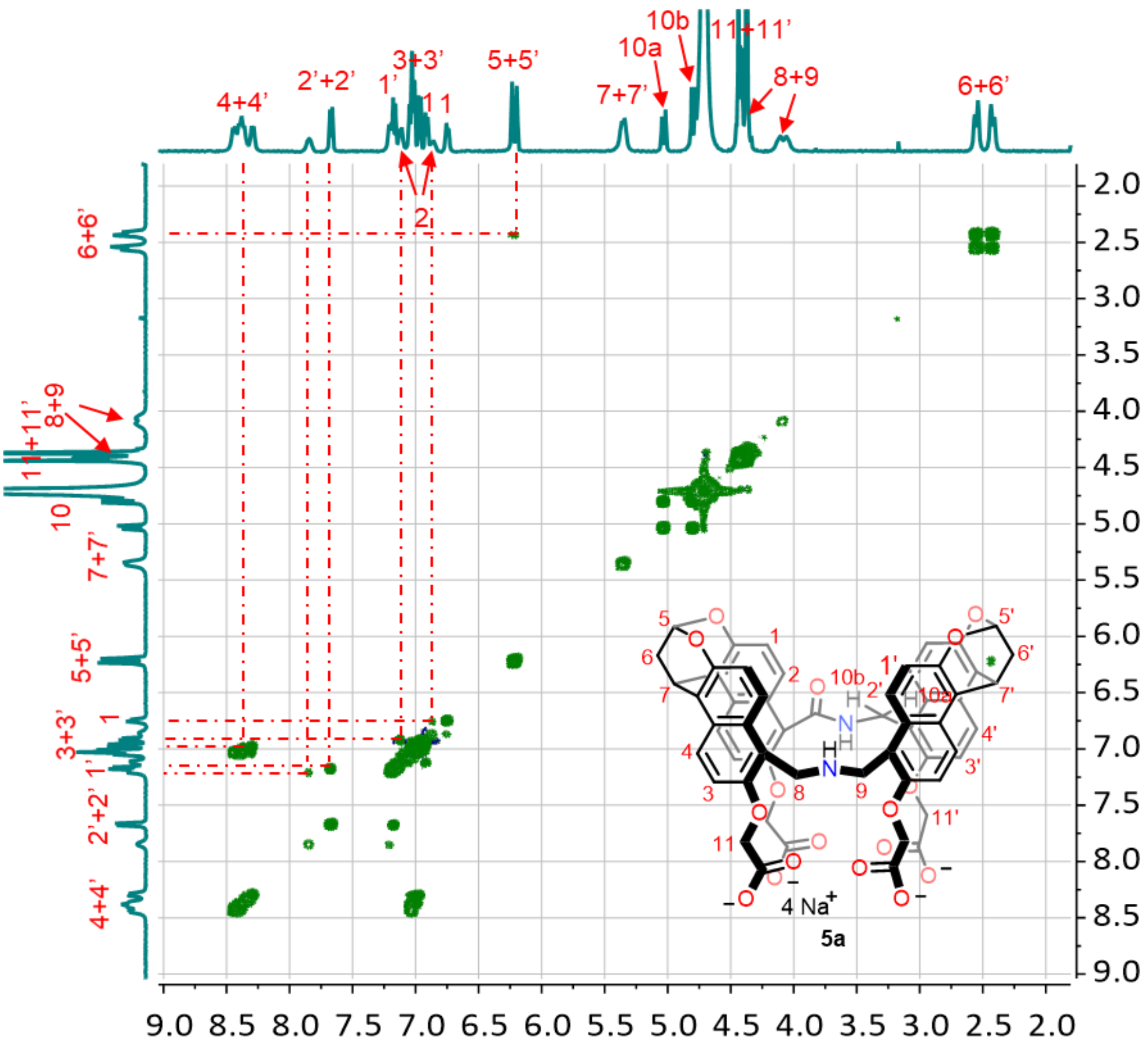

Supplementary Fig. 27 ^{1}H, ^{1}H-COSY NMR spectrum of **5a** (500 MHz, D_2O, 298 K).

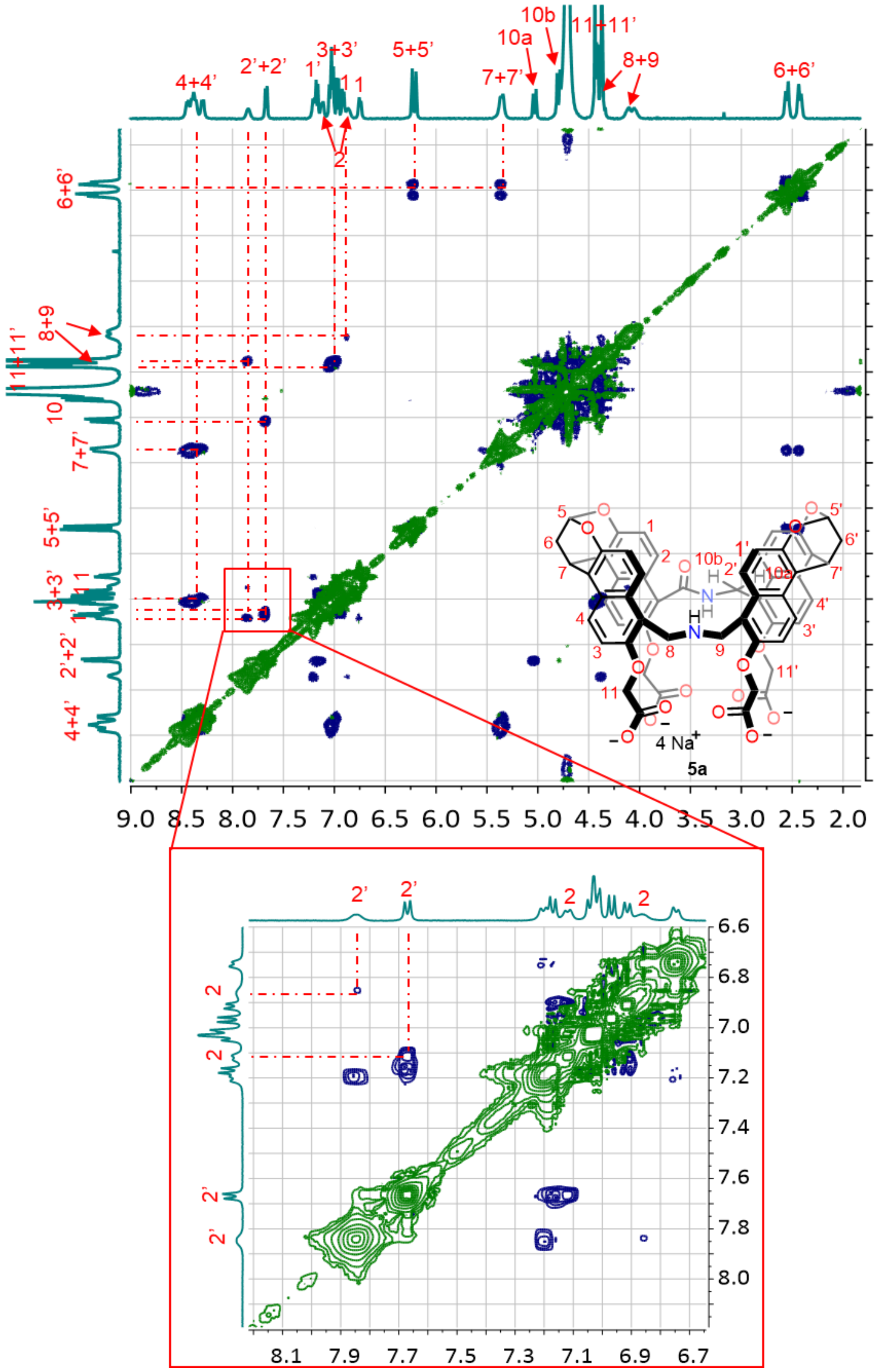

Supplementary Fig. 28 ^{1}H, ^{1}H-ROESY NMR spectrum of **5a** (500 MHz, D_2O, 298 K). NOE effect was detected between the protons 2 and protons 2'. This structure was assigned to the *syn* isomer, which was confirmed by the crystal structure of its precursor **4a**.

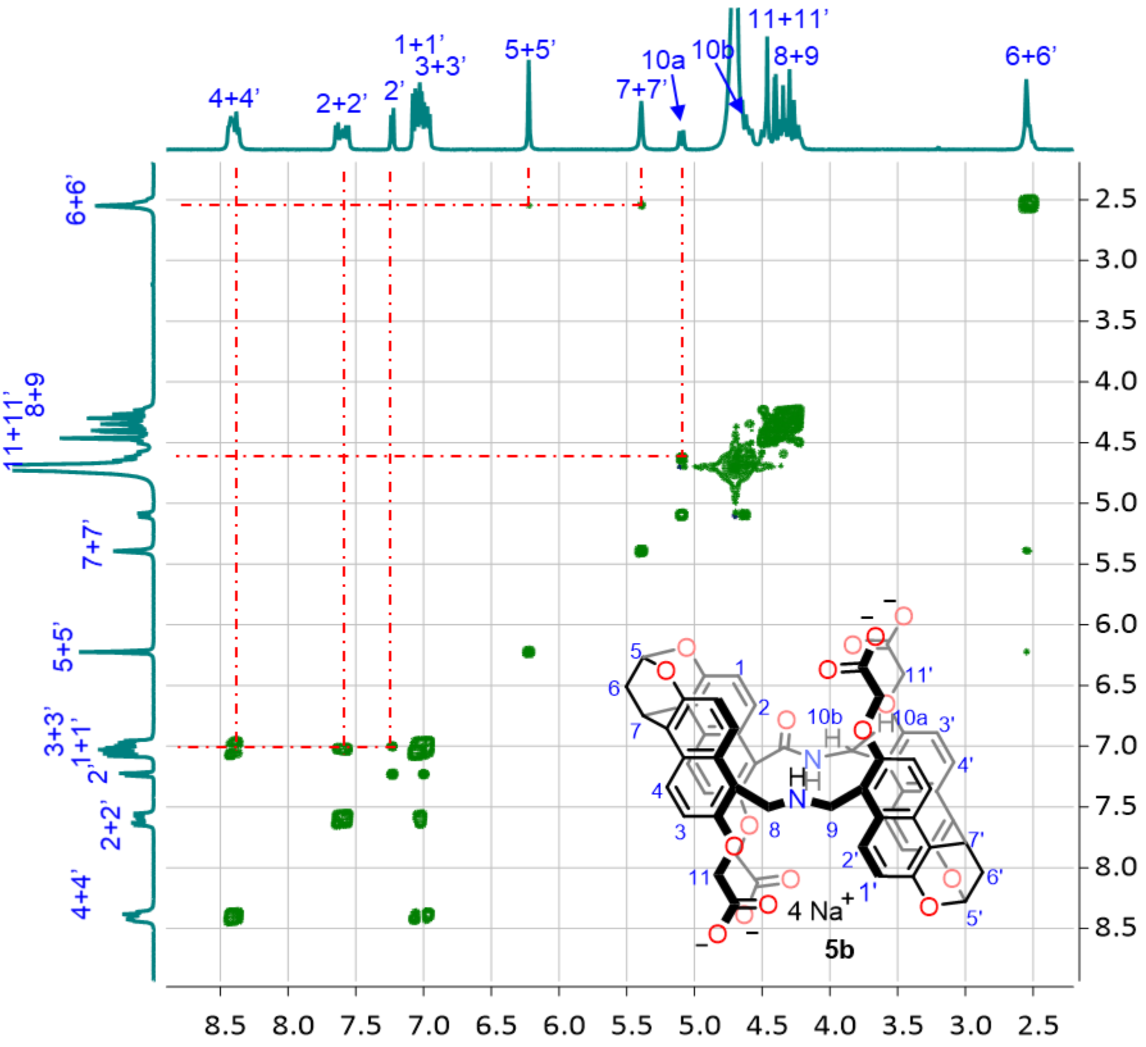

Supplementary Fig. 29 ^{1}H, ^{1}H-COSY NMR spectrum of **5b** (500 MHz, D_2O, 298 K).

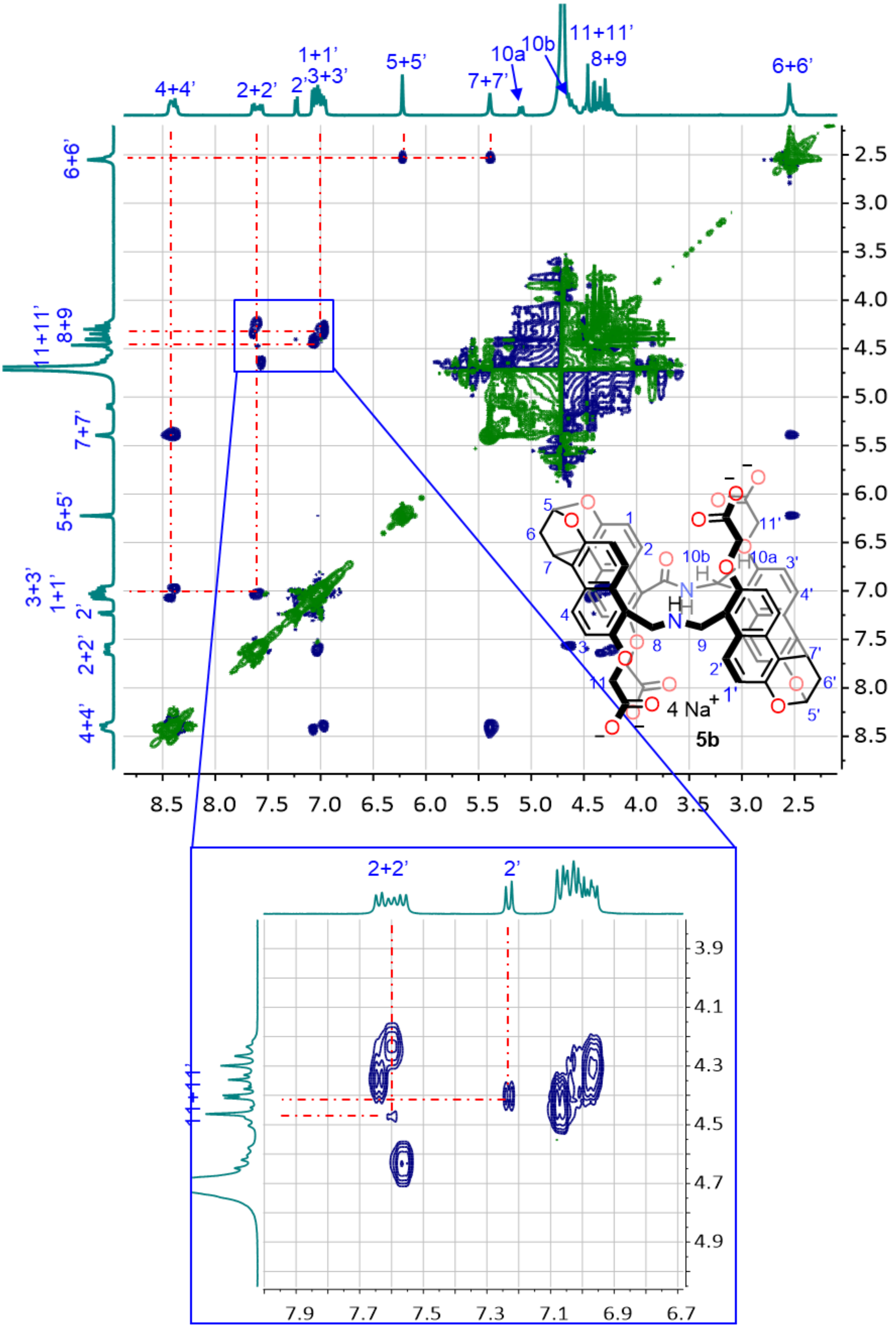

Supplementary Fig. 30 ^{1}H, ^{1}H-ROESY NMR spectrum of **5b** (500 MHz, D_2O, 298 K). The protons 2 have NOE contacts with the protons 2'. Therefore, this structure was assigned to the *anti* isomer, which was confirmed by the crystal structure of its precursor **4b**.

5. pK_a Values and Critical Aggregation Concentration of 5

5.1 pK_a Determiantion of 5a and 5b by ^{1}H NMR

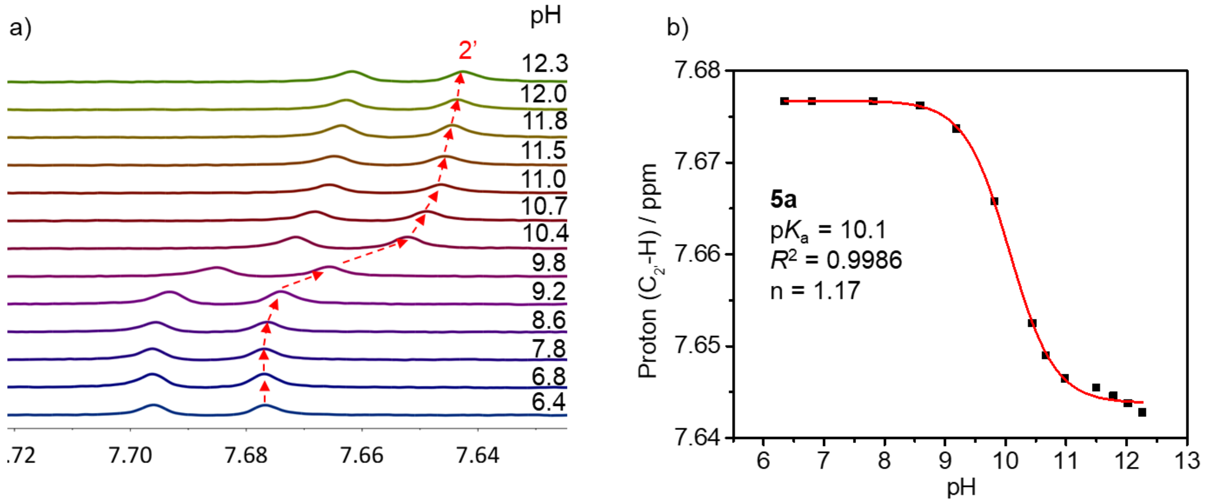

Supplementary Fig. 31 a) ^{1}H NMR chemical shift dependence on the ionization state of the **5a**. The proton signals of **5a** toward to up field with increasing pH. b) Titration curve tracking the change in chemical shift with pH for **5a**, which was fit to the Henderson-Hasselbalch equation (Supplementary Equation 1) to determine pK_a value and Hill coefficient. The pK_a for this trial is 10.1 with a Hill coefficient of 1.17.

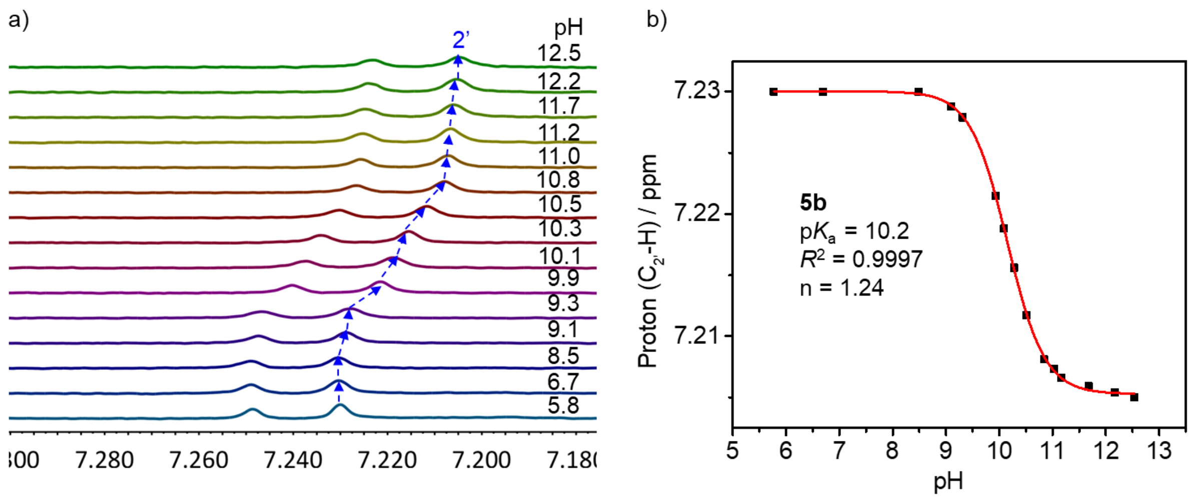

Supplementary Fig. 32 a) ^{1}H NMR chemical shift dependence on the ionization state of the **5b**. The proton signals of **5b** toward to up field with increasing pH. b) Titration curve tracking the change in chemical shift with pH for **5b**, which was fit to the Henderson- Hasselbalch equation (Supplementary Equation 1) to determine pK_a value and Hill coefficient. The pK_a for this trial is 10.2 with a Hill coefficient of 1.24.

5.2 pK_a Determiantion of 5a and 5b by Fluorescence Spectra

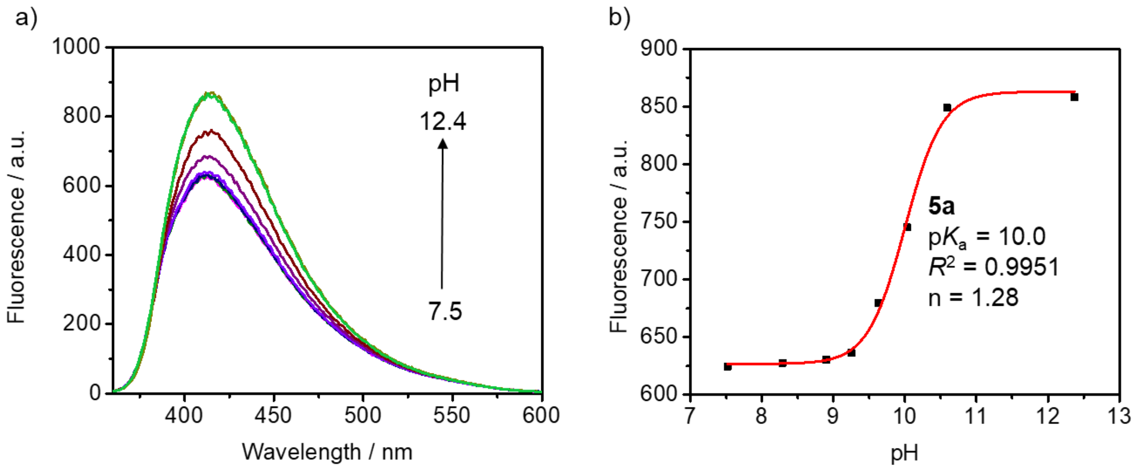

Supplementary Fig. 33 a) Fluorescence intensity dependence on the ionization state of the **5a**. The fluorescence intensity of **5a** enhanced with increasing pH. b) Titration curve tracking the change in fluorescence intensity with pH for **5a**, which was fit to the Henderson- Hasselbalch equation (Supplementary Equation 1) to determine pK_a value and Hill coefficient. The pK_a for this trial is 10.0 with a Hill coefficient of 1.28.

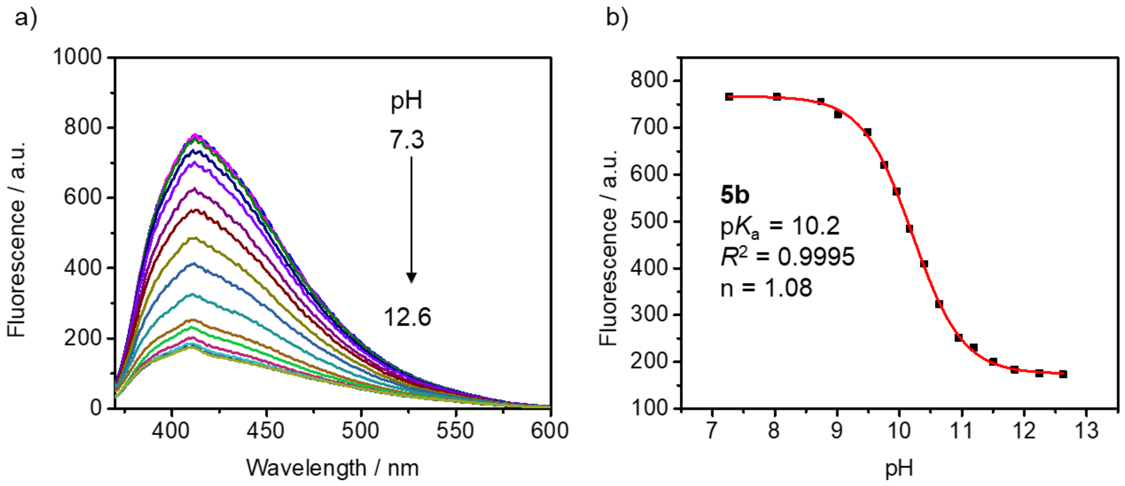

Supplementary Fig. 34 a) Fluorescence intensity dependence on the ionization state of the **5b**. The fluorescence intensity of **5b** quenched with increasing pH. b) Titration curve tracking the change in fluorescence intensity with pH for **5b**, which was fit to the Henderson-Hasselbalch equation (Supplementary Equation 1) to determine pK_a value and Hill coefficient. The pK_a for this trial is 10.2 with a Hill coefficient of 1.08.

Supplementary Table 2 Parameters from fits to pH titrations

	^{1}H NMR			Fluorescence spectra		
	pK_a	n	R^2	pK_a	n	R^2
**5a**	10.1	1.17	0.9986	10.0	1.28	0.9951
**5b**	10.2	1.24	0.9997	10.2	1.08	0.9995

5.3 Critical Aggregation Concentration of 5

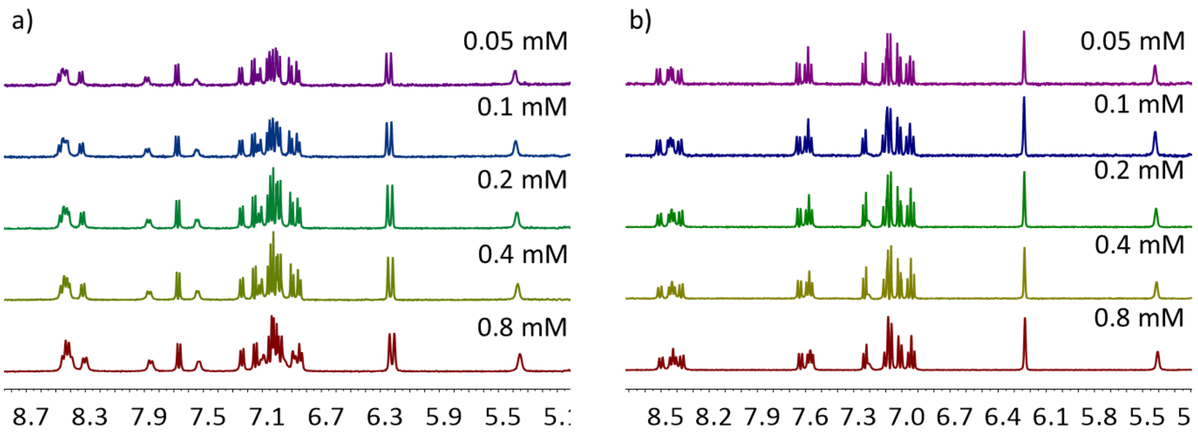

Supplementary Fig. 35 Partial ^{1}H NMR spectra (500 MHz, D_2O/H_2O = 1/9, 298 K) of (a) **5a** and (b) **5b** at different concentrations (0.8 mM – 0.05 mM) at pH 7.4 (phosphate buffer, 50 mM), respectively. The peaks of **5a** and **5b** show sharp and similar at a concentration range of 0.8 mM – 0.05 mM, suggesting the host exists in unimolecular form below 0.8 mM at pH7.4.

5.4 pK_a Values for the Conjugate Acids Guests

Supplementary Table 3 pK_a values for the conjugate acids of guests **G1** – **G4** and **G6 - G7**

Guests	pK_a	Guests	pK_a
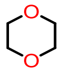 **G1**	-2.9[4]	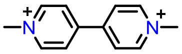 **G5**	-
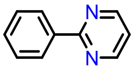 **G2**	1.0[5]	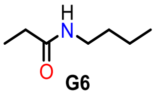 **G6**	-2.0[6]
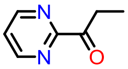 **G3**	-1.1[6]	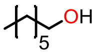 **G7**	-2.3[6]
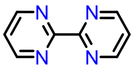 **G4**	-1.2[5]		

6. ^{1}H NMR Spectra of Host-Guest Complexes

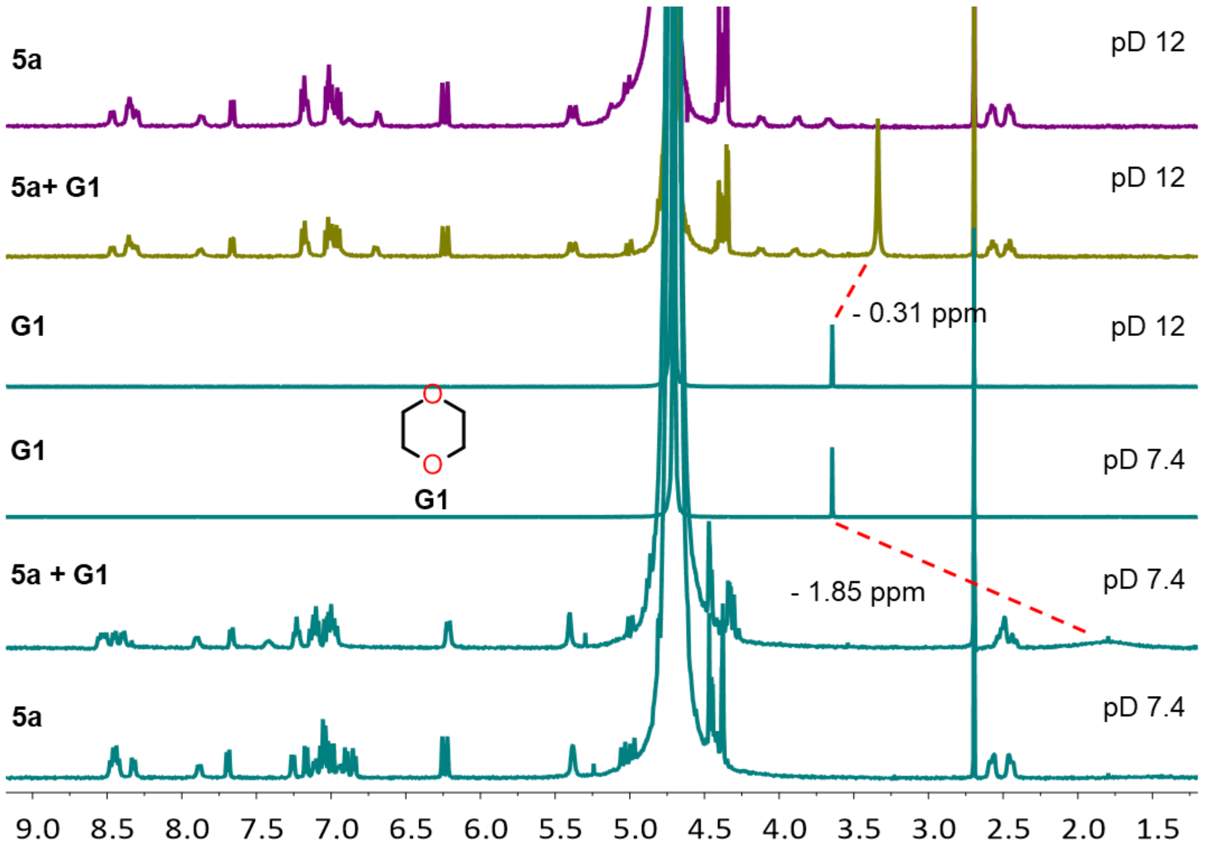

Supplementary Fig. 36 ^{1}H NMR spectra (500 MHz, D_2O, 0.4 mM, 298 K) of **G1**, **5a**, and their equimolar mixture in pD 7.4 and 12 (phosphate buffer, 50 mM), respectively. The protons of **G1** undergo more significant upfield shift in pD 7.4 than that in pD 12, suggesting that the binding behavior between **5a** and **G1** in pD 7.4 phosphate buffer is stronger than that in pD 12.

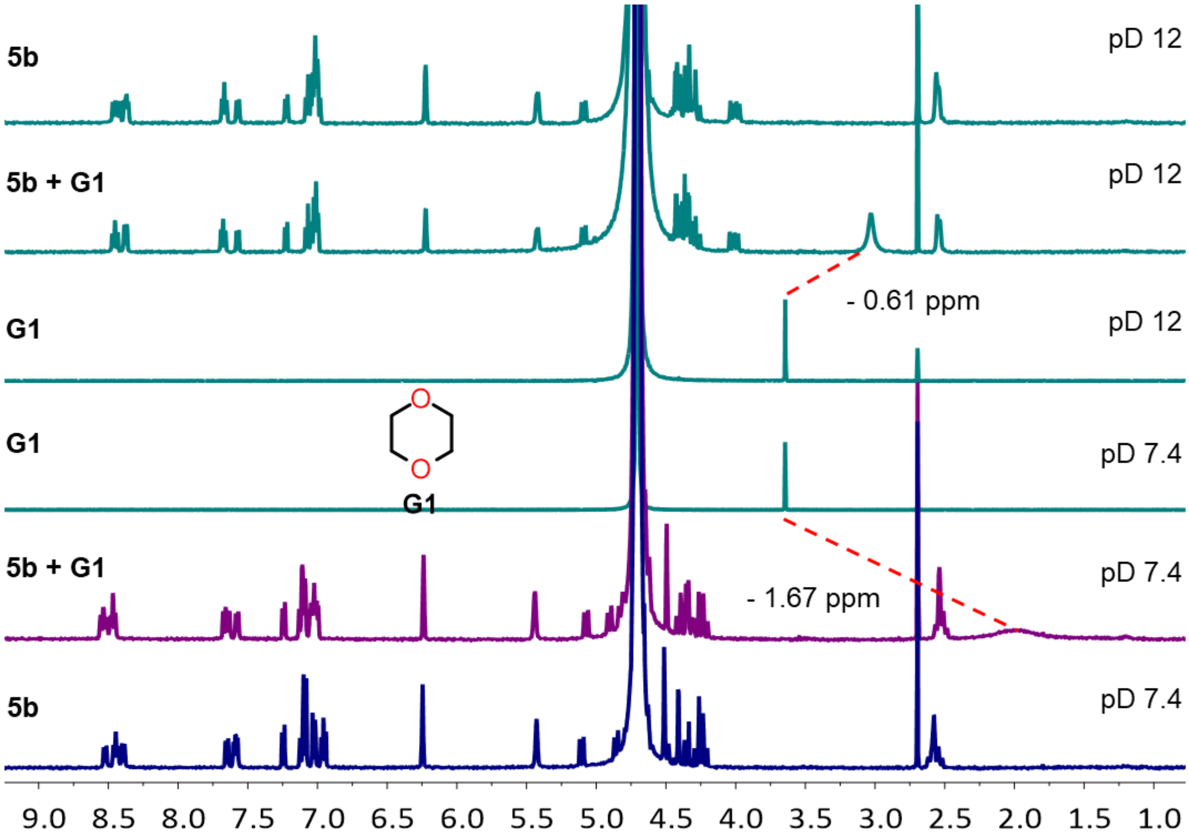

Supplementary Fig. 37 ^{1}H NMR spectra (500 MHz, D_2O, 0.4 mM, 298 K) of **G1**, **5b**, and their equimolar mixture in pD 7.4 and 12 (phosphate buffer, 50 mM), respectively. The protons of **G1** undergo more significant upfield shift in pD 7.4 than that in pD 12, suggesting that the binding behavior between **5b** and **G1** in pD 7.4 phosphate buffer is stronger than that in pD 12.

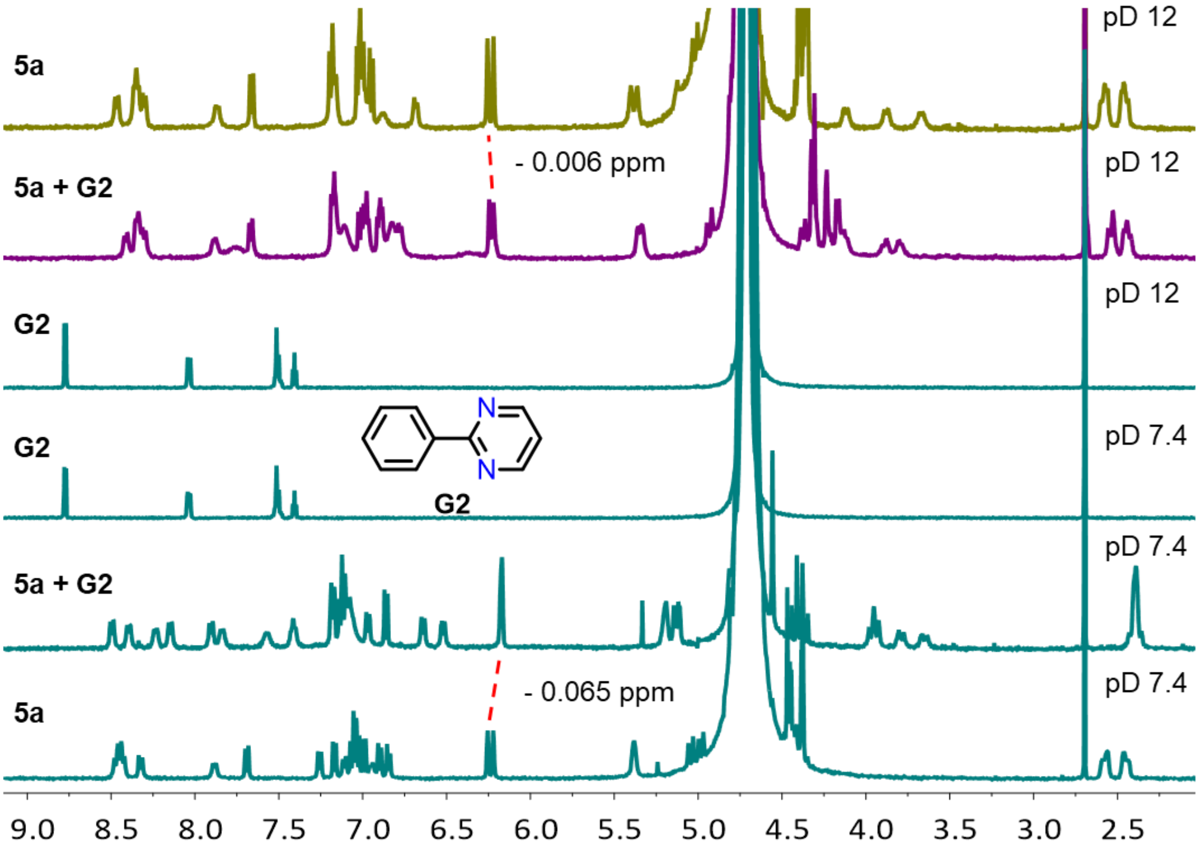

Supplementary Fig. 38 ^{1}H NMR spectra (500 MHz, D_2O, 0.4 mM, 298 K) of **G2**, **5a**, and their equimolar mixture in pD 7.4 and 12 (phosphate buffer, 50 mM), respectively. The protons of **5a** undergo more significant upfield shift in pD 7.4 than that in pD 12, suggesting that the binding behavior between **5a** and **G2** in pD 7.4 phosphate buffer is stronger than that in pD 12.

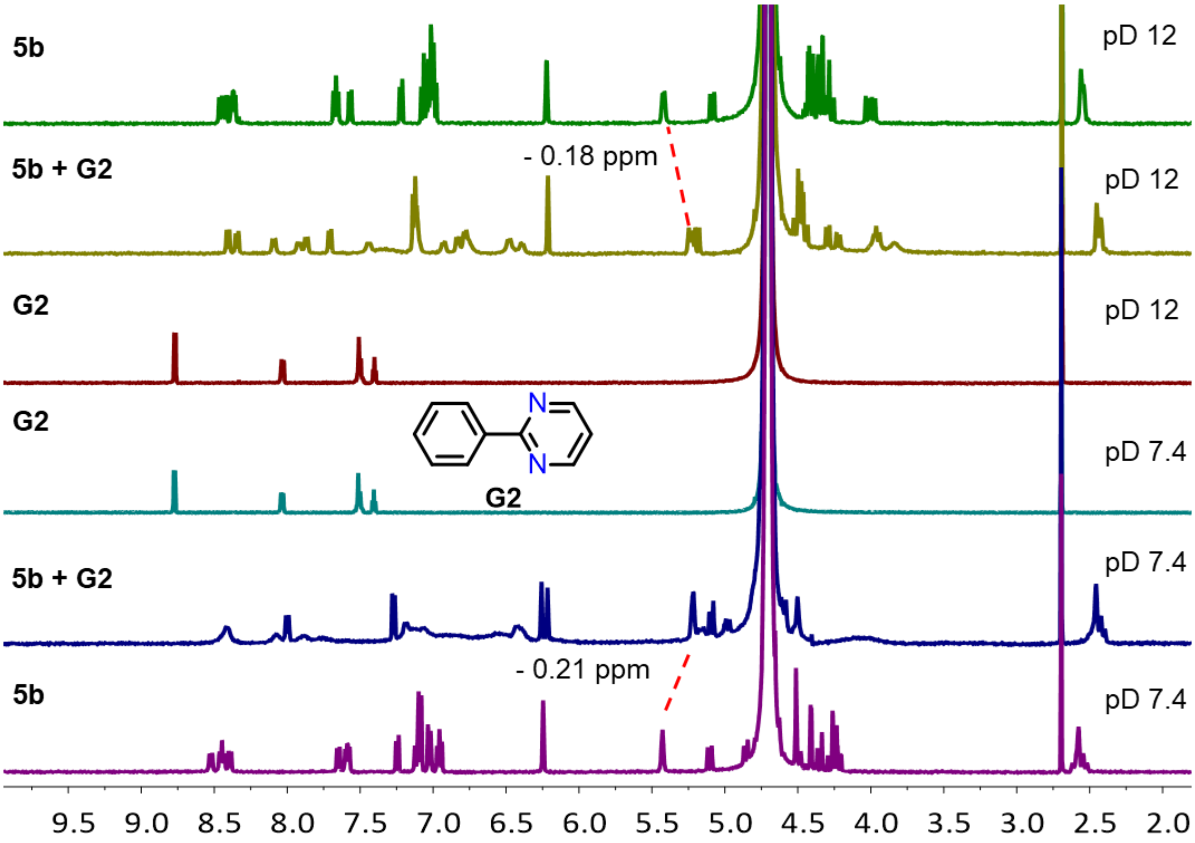

Supplementary Fig. 39 ^{1}H NMR spectra (500 MHz, D_2O, 0.4 mM, 298 K) of **G2**, **5b**, and their equimolar mixture in pD 7.4 and 12 (phosphate buffer, 50 mM), respectively. The protons of **5b** undergo more significant shifts in pD 7.4 than that in pD 12, suggesting that the binding behavior between **5b** and **G2** in pD 7.4 phosphate buffer is stronger than that in pD 12.

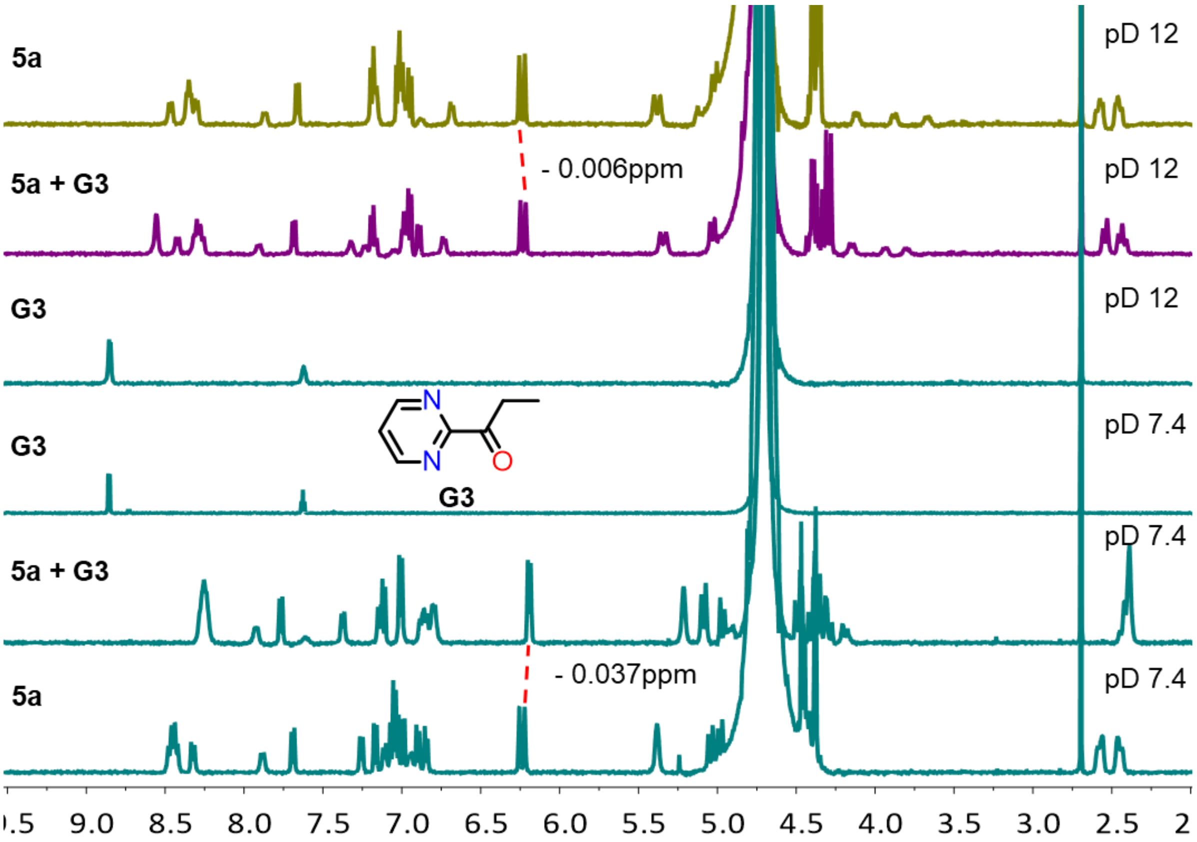

Supplementary Fig. 40 ^{1}H NMR spectra (500 MHz, D_2O, 0.4 mM, 298 K) of **G3**, **5a**, and their equimolar mixture in pD 7.4 and 12 (phosphate buffer, 50 mM), respectively. The protons of **5a** undergo more significant shifts in pD 7.4 than that in pD 12, suggesting that the binding behavior between **5a** and **G3** in pD 7.4 phosphate buffer is stronger than that in pD 12.

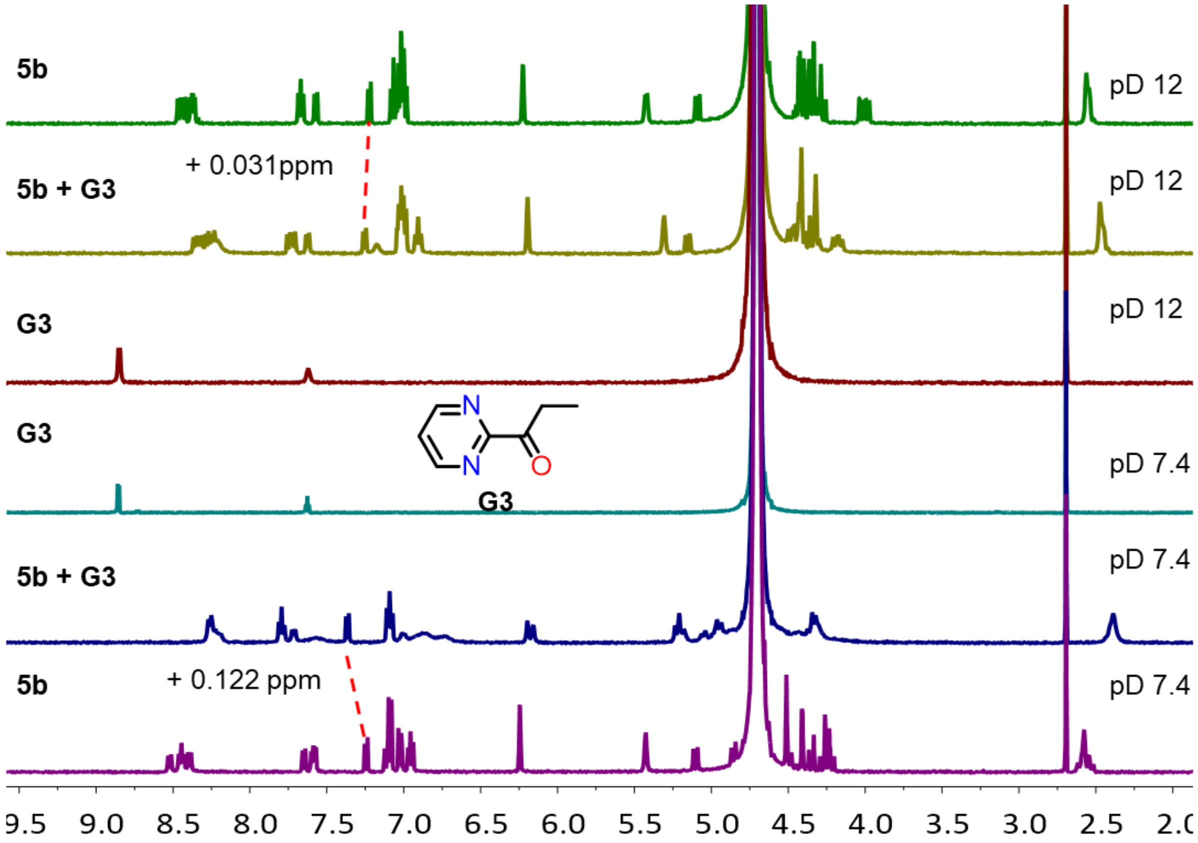

Supplementary Fig. 41 ^{1}H NMR spectra (500 MHz, D_2O, 0.4 mM, 298 K) of **G3**, **5b**, and their equimolar mixture in pD 7.4 and 12 (phosphate buffer, 50 mM), respectively. The protons of **5b** undergo more significant shifts in pD 7.4 than that in pD 12, suggesting that the binding behavior between **5b** and **G3** in pD 7.4 phosphate buffer is stronger than that in pD 12.

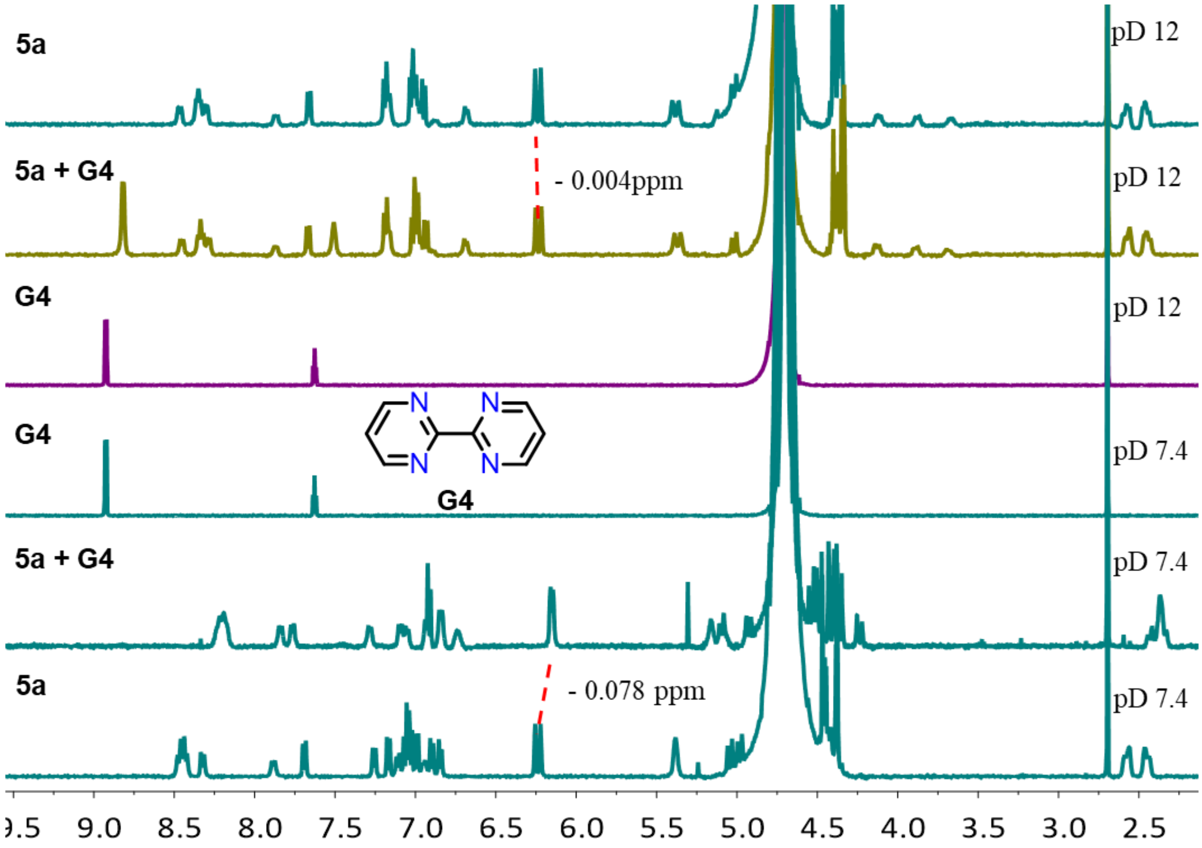

Supplementary Fig. 42 ^{1}H NMR spectra (500 MHz, D_2O, 0.4 mM, 298 K) of **G4**, **5a**, and their equimolar mixture in pD 7.4 and 12 (phosphate buffer, 50 mM), respectively. The protons of **5a** undergo more significant shifts in pD 7.4 than that in pD 12, suggesting that the binding behavior between **5a** and **G4** in pD 7.4 phosphate buffer is stronger than that in pD 12.

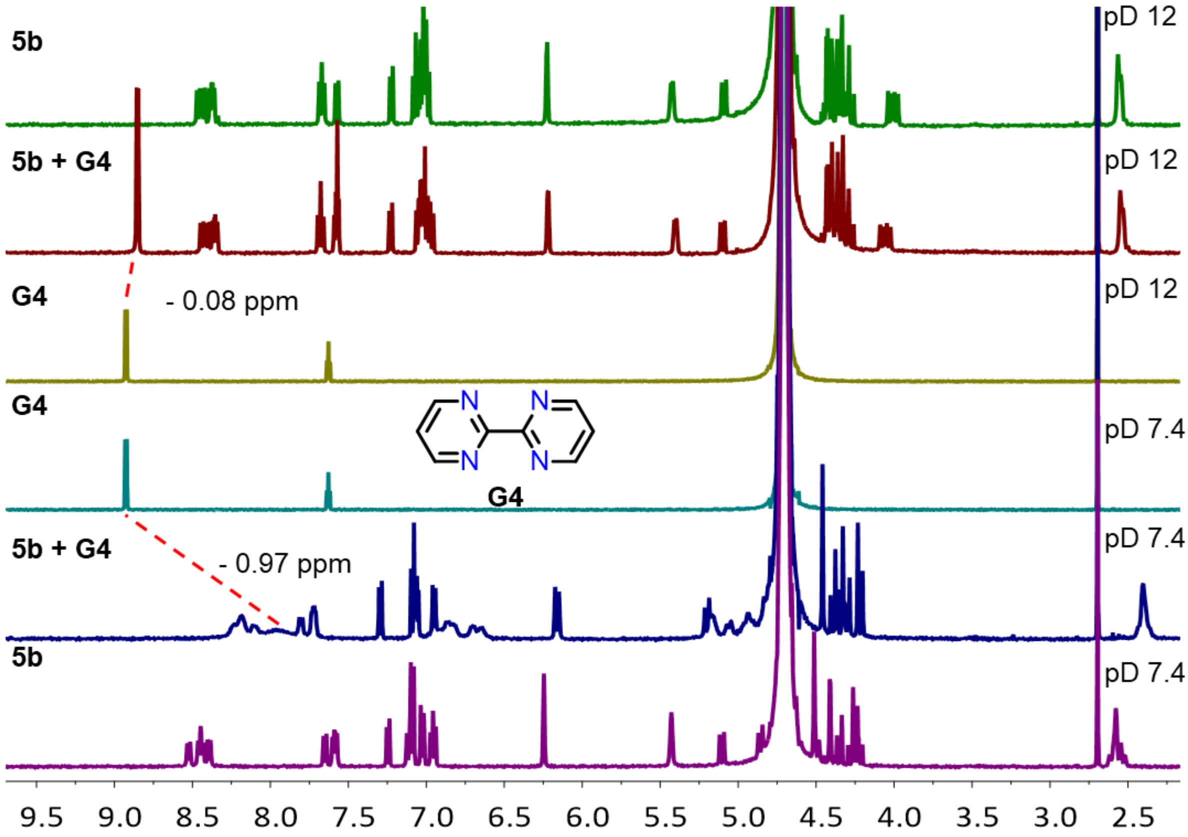

Supplementary Fig. 43 ^{1}H NMR spectra (500 MHz, D_2O, 0.4 mM, 298 K) of **G4**, **5b**, and their equimolar mixture in pD 7.4 and 12 (phosphate buffer, 50 mM), respectively. The protons of **G4** undergo more significant upfield shift in pD 7.4 than that in pD 12, suggesting that the binding behavior between **5b** and **G4** in pD 7.4 phosphate buffer is stronger than that in pD 12.

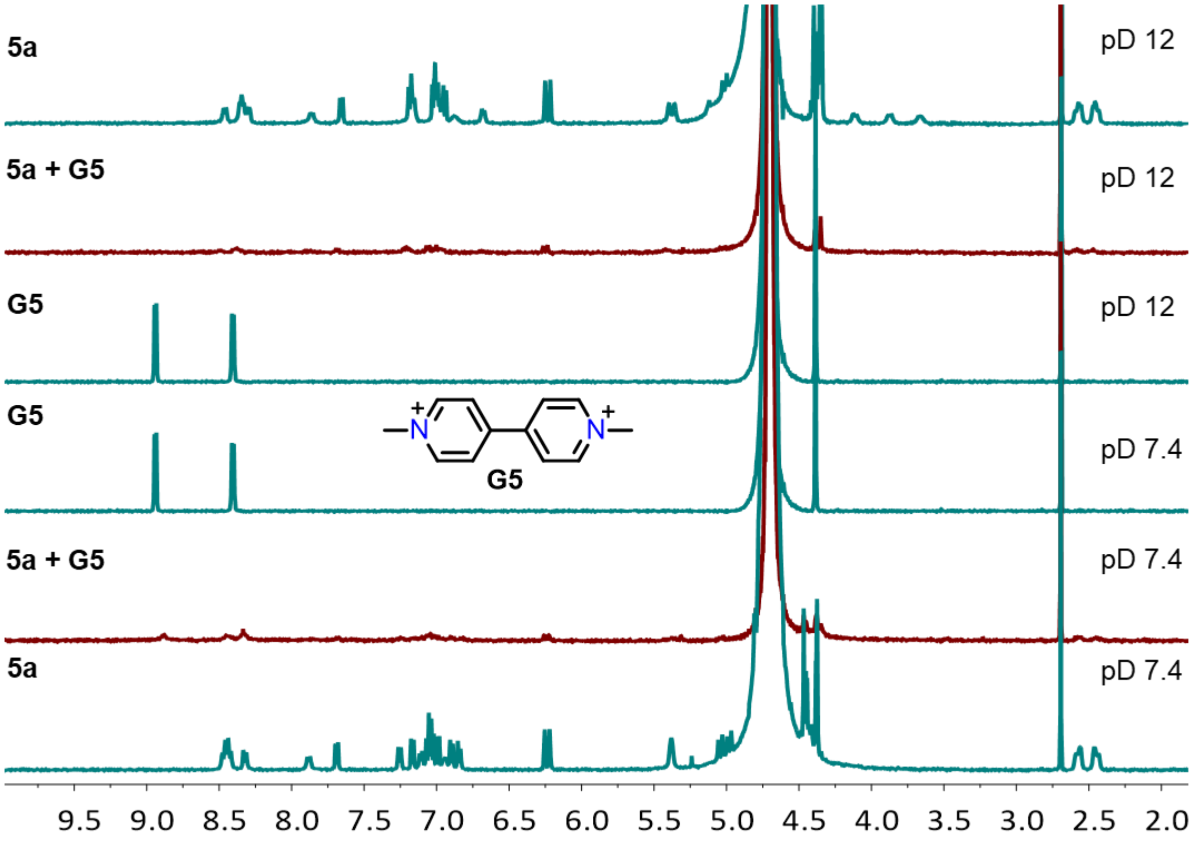

Supplementary Fig. 44 ^{1}H NMR spectra (500 MHz, D_2O, 0.4 mM, 298 K) of **G5**, **5a**, and their equimolar mixture in pD 7.4 and 12 (phosphate buffer, 50 mM), respectively. The peaks of **5a** and **G5** almost disappear into the baseline, which indicates the severe aggregation in the host-guest mixture at pD 7.4 and 12 (phosphate buffer, 50 mM), respectively.

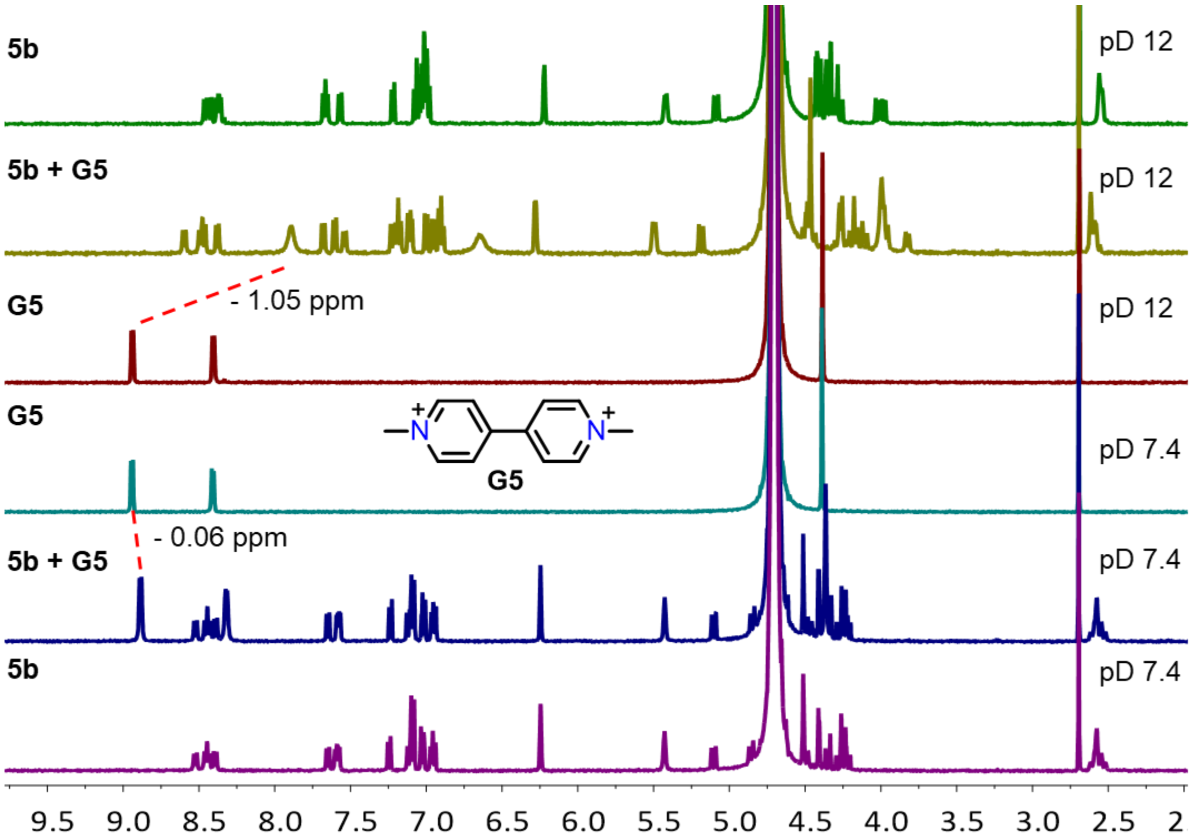

Supplementary Fig. 45 ^{1}H NMR spectra (500 MHz, D_2O, 0.4 mM, 298 K) of **G5**, **5b**, and their equimolar mixture in pD 7.4 and 12 (phosphate buffer, 50 mM), respectively. The protons of **G5** undergo more significant upfield shift in pD 12 than that in pD 7.4, suggesting that the binding behavior between **5b** and **G5** in pD 12 phosphate buffer is stronger than that in pD 7.4.

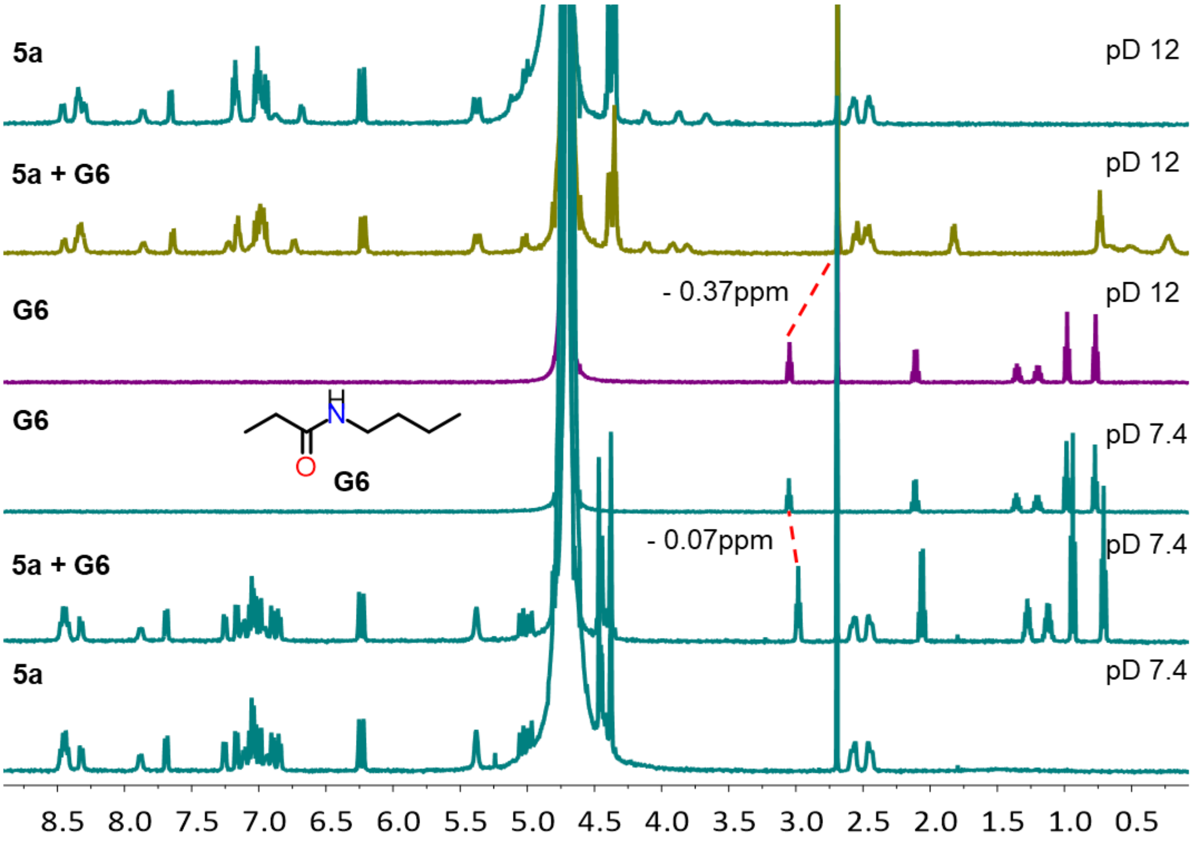

Supplementary Fig. 46 ^{1}H NMR spectra (500 MHz, D_2O, 0.4 mM, 298 K) of **G6**, **5a**, and their equimolar mixture in pD 7.4 and 12 (phosphate buffer, 50 mM), respectively. The protons of **G6** undergo more significant upfield shift in pD 12 than that in pD 7.4, suggesting that the binding behavior between **5a** and **G6** in pD 12 phosphate buffer is stronger than that in pD 7.4.

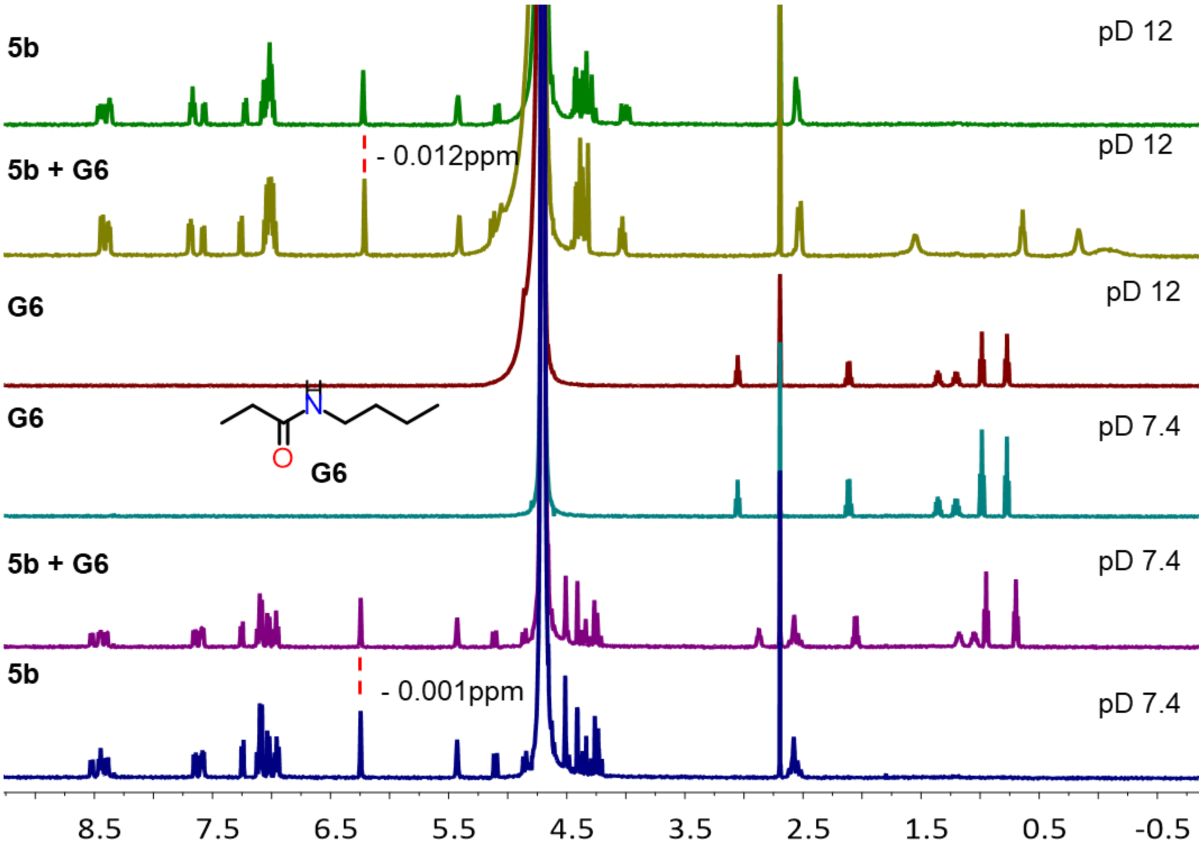

Supplementary Fig. 47 ^{1}H NMR spectra (500 MHz, D_2O, 0.4 mM, 298 K) of **G6**, **5b**, and their equimolar mixture in pD 7.4 and 12 (phosphate buffer, 50 mM), respectively. The protons of **5b** undergo more significant shifts in pD 12 than in pD 7.4, suggesting that there is a stronger binding affinites between **5b** and **G6** in pD 12 phosphate buffer than that in pD 7.4.

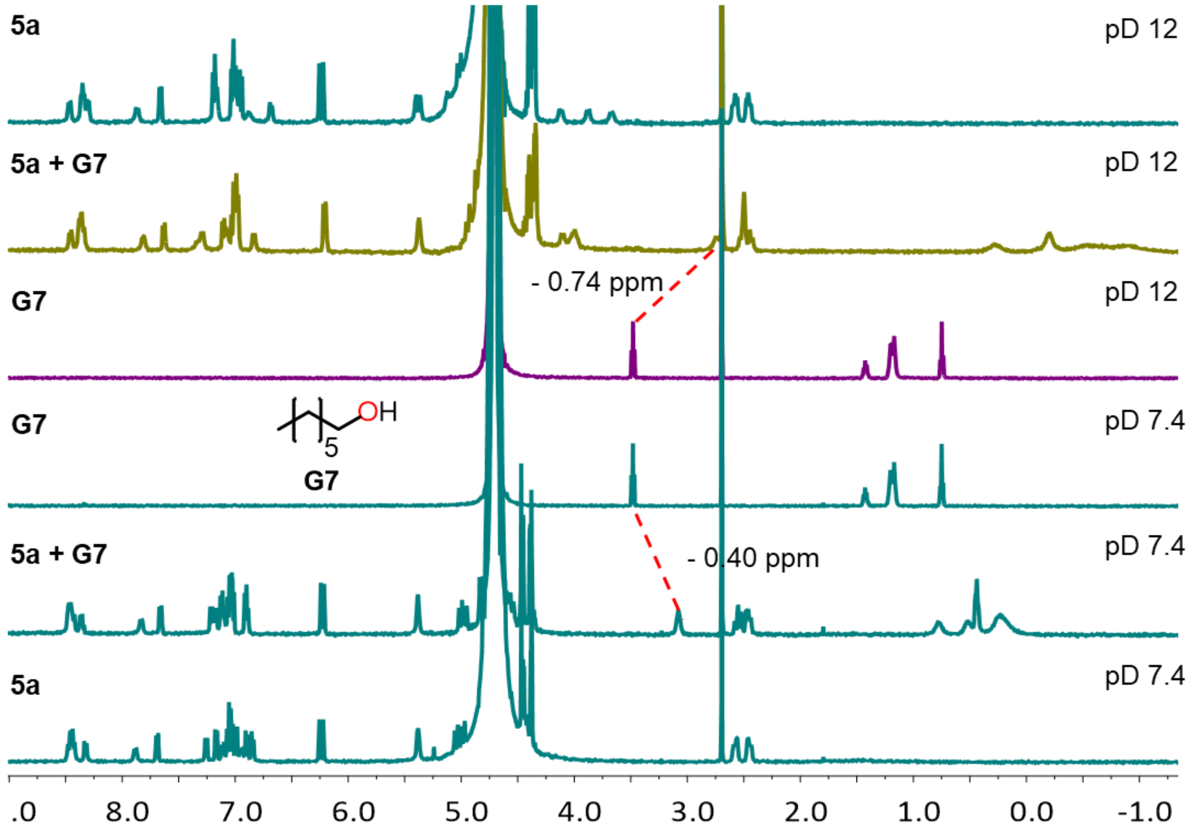

Supplementary Fig. 48 ^{1}H NMR spectra (500 MHz, D_2O, 0.4 mM, 298 K) of **G7**, **5a**, and their equimolar mixture in pD 7.4 and 12 (phosphate buffer, 50 mM), respectively. The protons of **G7** undergo more significant upfield shift in pD 12 than that in pD 7.4, suggesting that the binding behavior between **5a** and **G7** in pD 12 phosphate buffer is stronger than that in pD 7.4.

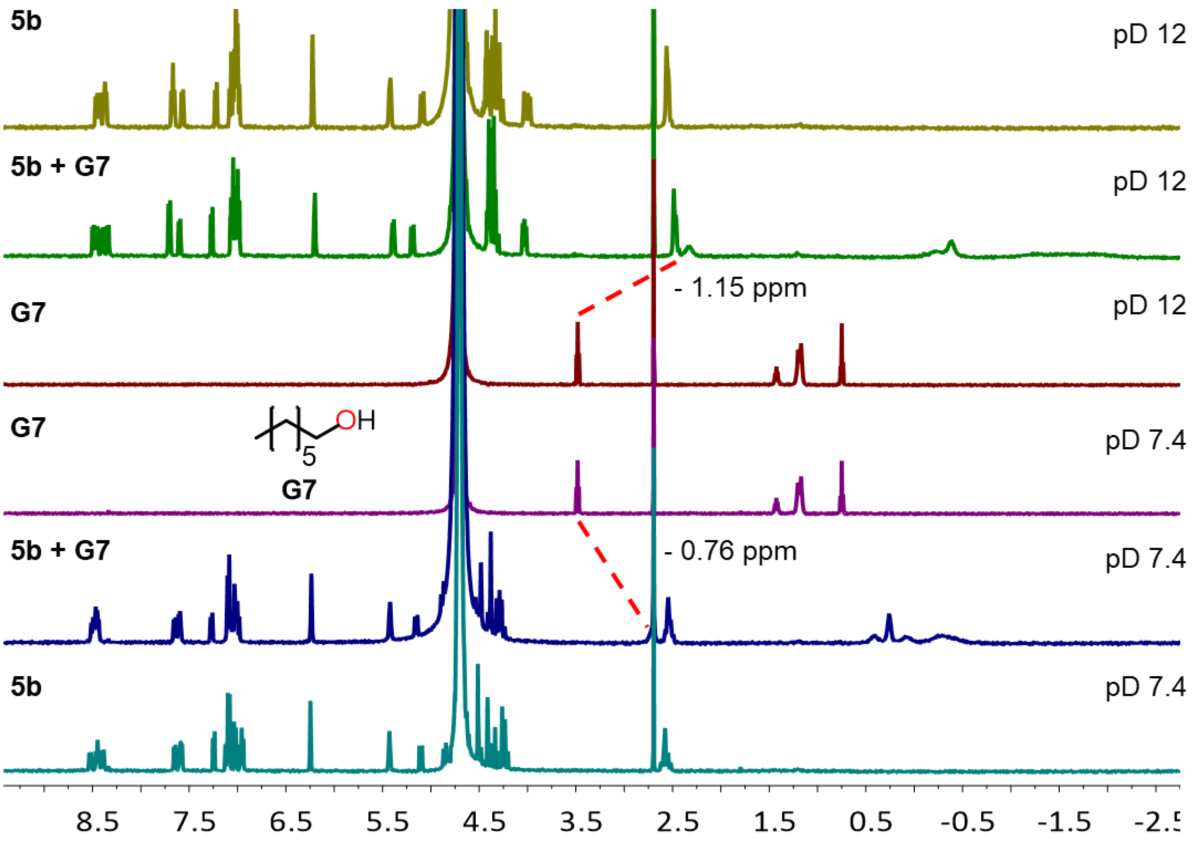

Supplementary Fig. 49 ^{1}H NMR spectra (500 MHz, D_2O, 0.4 mM, 298 K) of **G7**, **5b**, and their equimolar mixture in pD 7.4 and 12 (phosphate buffer, 50 mM), respectively. The protons of **G7** undergo more significant upfield shift in pD 12 than that in pD 7.4, suggesting that the binding behavior between **5b** and **G7** in pD 12 phosphate buffer is stronger than that in pD 7.4.

7. Determination of Association Constants by NMR Titrations

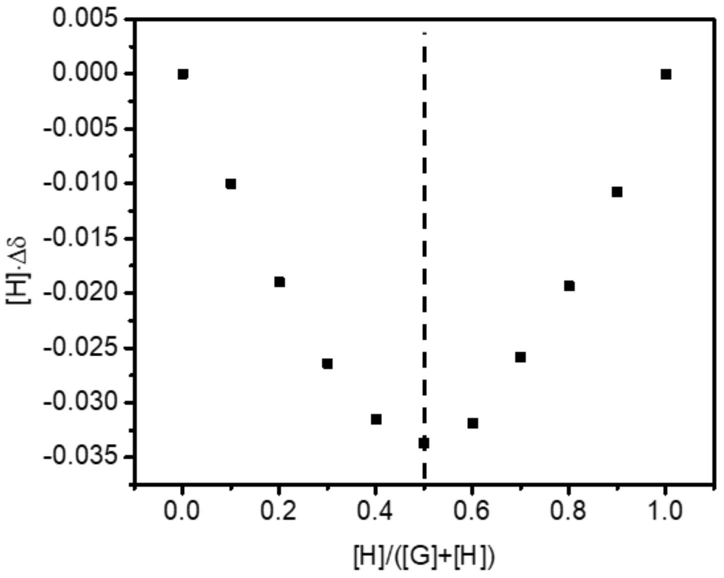

Supplementary Fig. 50 Job plot constructed from the chemical shift change (Δδ) of $C_{5'}$-H of **5a** in ^{1}H NMR spectra by varying the ratio of **G4** and **5a** with a fixed total concentration ([**5a**]+[**G4**] = 0.5 mM). This experiment supports the 1:1 binding stoichiometry between **5a** and **G4** in phosphate buffer (50 mM, pH = 7.4).

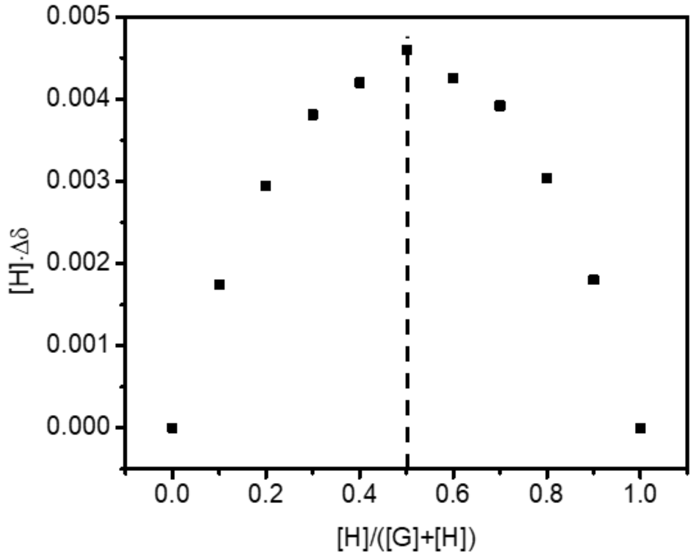

Supplementary Fig. 51 Job plot constructed from the chemical shift change (Δδ) of $C_{2'}$-H of **5a** in ^{1}H NMR spectra by varying the ratio of **G4** and **5a** with a fixed total concentration ([**5a**]+[**G4**] = 0.5 mM). This experiment supports the 1:1 binding stoichiometry between **5a** and **G4** in phosphate buffer (50 mM, pH = 12).

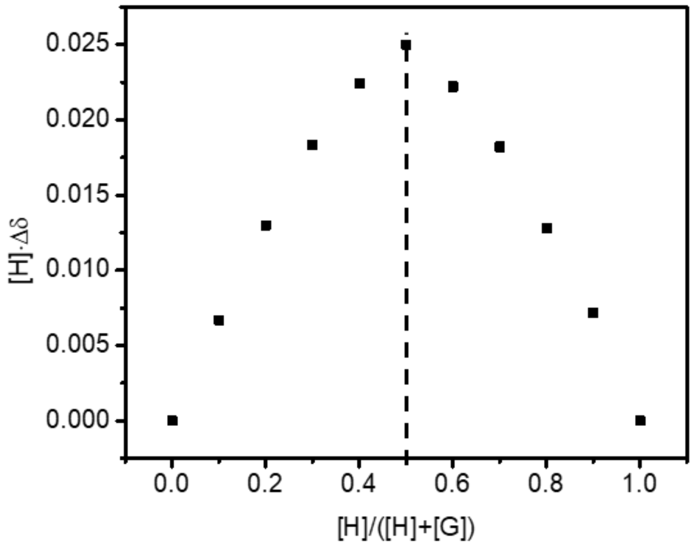

Supplementary Fig. 52 Job plot constructed from the chemical shift change (Δδ) of $C_{2'}$-H of **5b** in ^{1}H NMR spectra by varying the ratio of **G4** and **5b** with a fixed total concentration ([**5b**]+[**G4**] = 0.5 mM). This experiment supports the 1:1 binding stoichiometry between **5b** and **G4** in phosphate buffer (50 mM, pH = 7.4).

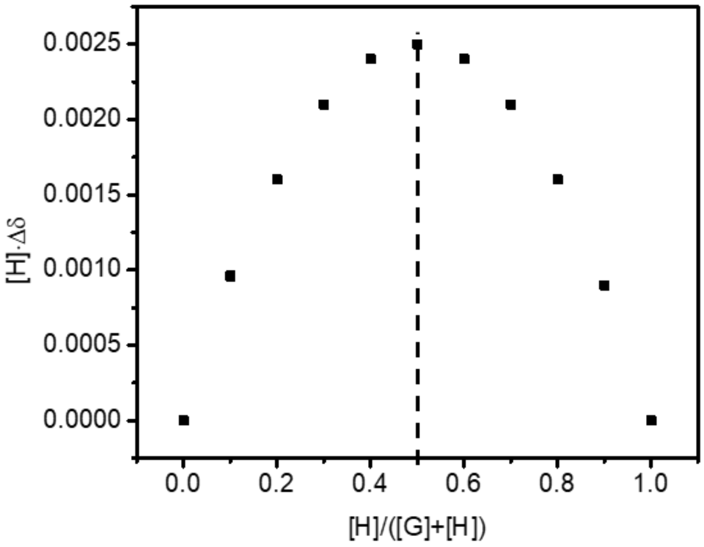

Supplementary Fig. 53 Job plot constructed from the chemical shift change (Δδ) of $C_{2'}$-H of **5b** in ^{1}H NMR spectra by varying the ratio of **G4** and **5b** with a fixed total concentration ([**5b**]+[**G4**] = 0.5 mM). This experiment supports the 1:1 binding stoichiometry between **5b** and **G4** in phosphate buffer (50 mM, pH = 12).

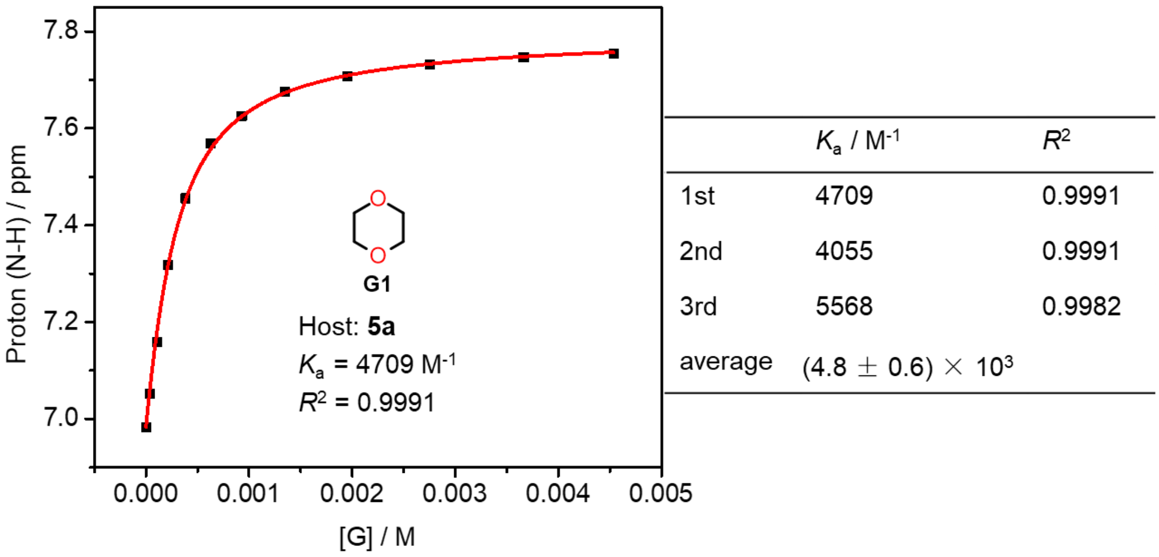

	K_a / M^{-1}	R^2
1st	4709	0.9991
2nd	4055	0.9991
3rd	5568	0.9982
average	$(4.8 \pm 0.6) \times 10^3$	

Supplementary Fig. 54 Non-linear curve-fitting for the complexation between **5a** and **G1** in phosphate buffer (50 mM, pH = 7.4, D_2O/H_2O = 1:9) at 298 K. K_a was given by averaging the values obtained from three independent titrations.

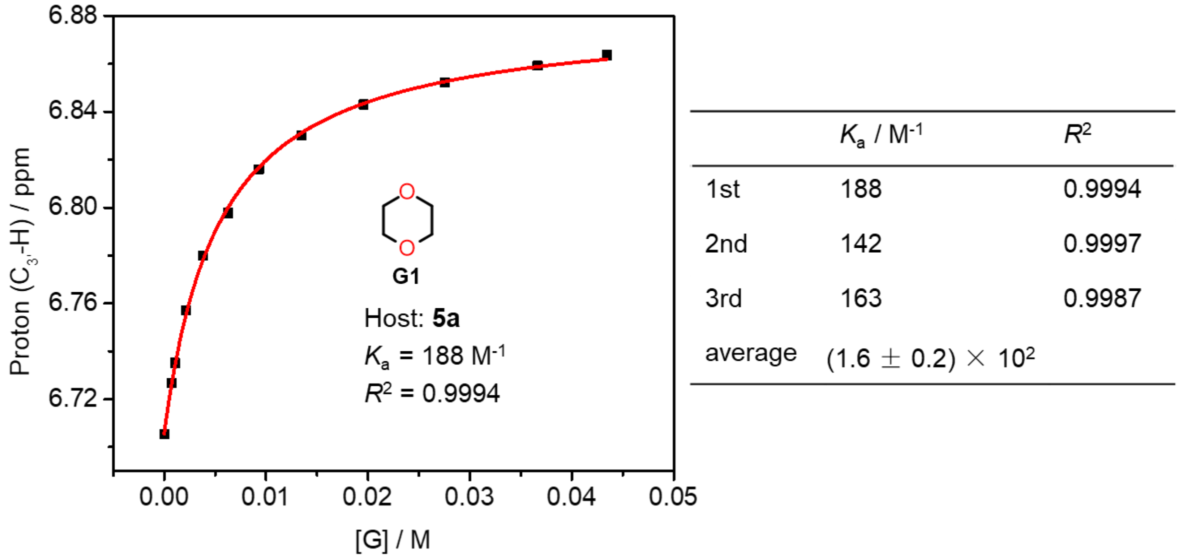

	K_a / M^{-1}	R^2
1st	188	0.9994
2nd	142	0.9997
3rd	163	0.9987
average	$(1.6 \pm 0.2) \times 10^2$	

Supplementary Fig. 55 Non-linear curve-fitting for the complexation between **5a** and **G1** in phosphate buffer (50 mM, pH = 12, D_2O/H_2O = 1:9) at 298 K. K_a was given by averaging the values obtained from three independent titrations.

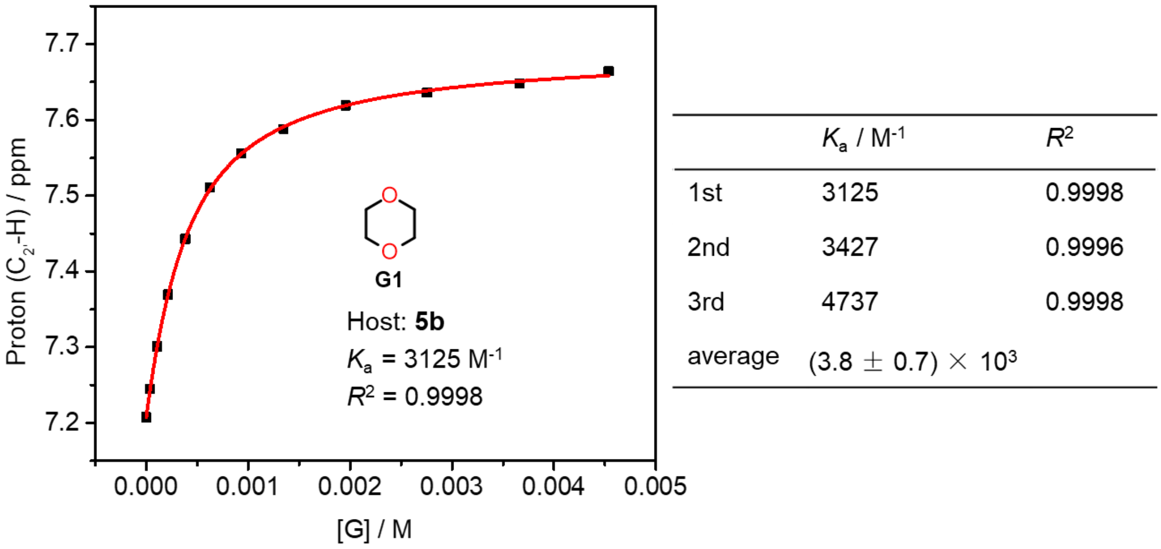

	K_a / M^{-1}	R^2
1st	3125	0.9998
2nd	3427	0.9996
3rd	4737	0.9998
average	$(3.8 \pm 0.7) \times 10^3$	

Supplementary Fig. 56 Non-linear curve-fitting for the complexation between **5b** and **G1** in phosphate buffer (50 mM, pH = 7.4, D_2O/H_2O = 1:9) at 298 K. K_a was given by averaging the values obtained from three independent titrations.

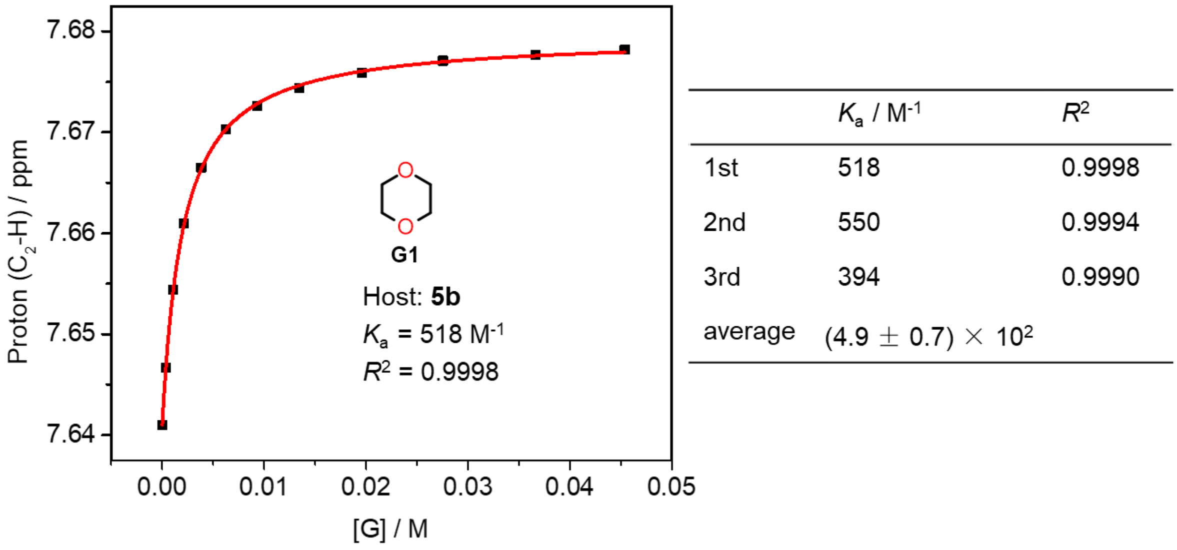

	K_a / M^{-1}	R^2
1st	518	0.9998
2nd	550	0.9994
3rd	394	0.9990
average	$(4.9 \pm 0.7) \times 10^2$	

Supplementary Fig. 57 Non-linear curve-fitting for the complexation between **5b** and **G1** in phosphate buffer (50 mM, pH = 12, D_2O/H_2O = 1:9) at 298 K. K_a was given by averaging the values obtained from three independent titrations.

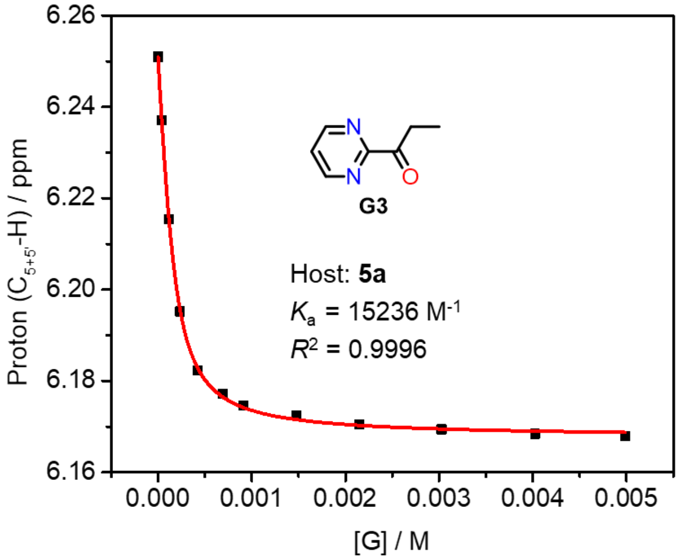

	K_a / M^{-1}	R^2
1st	15326	0.9996
2nd	19615	0.9997
3rd	21392	0.9995
average	$(1.9 \pm 0.3) \times 10^4$	

Supplementary Fig. 58 Non-linear curve-fitting for the complexation between **5a** and **G3** in phosphate buffer (50 mM, pH = 7.4, D_2O/H_2O = 1:9) at 298 K. K_a was given by averaging the values obtained from three independent titrations.

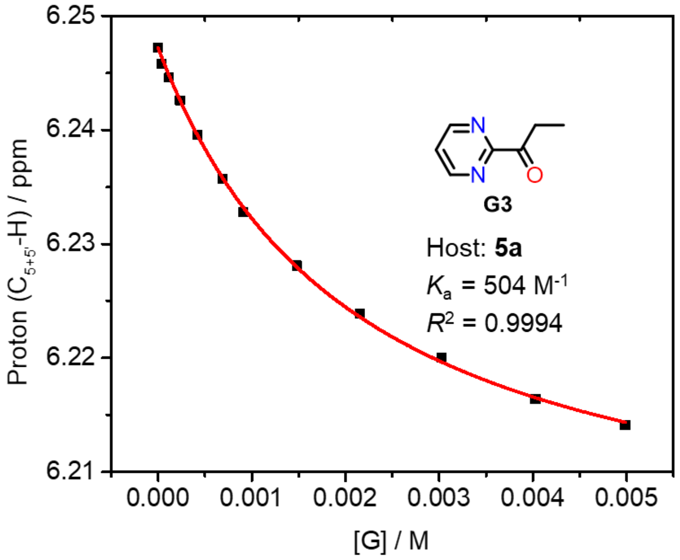

	K_a / M^{-1}	R^2
1st	504	0.9994
2nd	523	0.9978
3rd	720	0.9976
average	$(6 \pm 1) \times 10^2$	

Supplementary Fig. 59 Non-linear curve-fitting for the complexation between **5a** and **G3** in phosphate buffer (50 mM, pH = 12, D_2O/H_2O = 1:9) at 298 K. K_a was given by averaging the values obtained from three independent titrations.

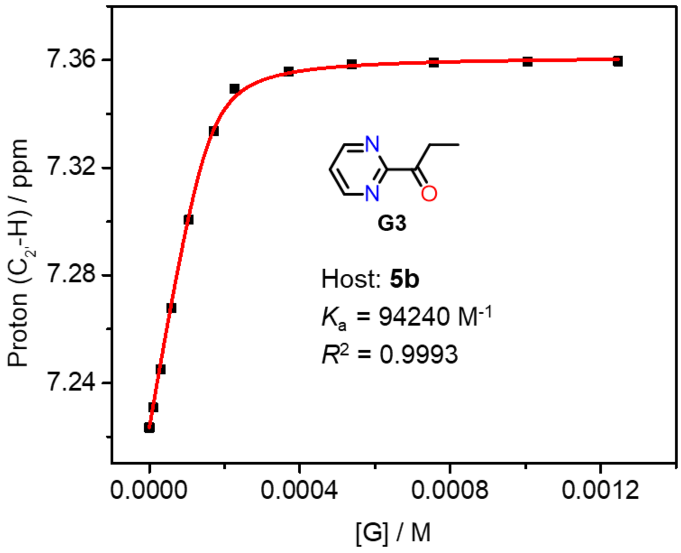

	K_a / M^{-1}	R^2
1st	94240	0.9993
2nd	107480	0.9992
3rd	116002	0.9992
average	$(1.1 \pm 0.1) \times 10^5$	

Supplementary Fig. 60 Non-linear curve-fitting for the complexation between **5b** and **G3** in phosphate buffer (50 mM, pH = 7.4, D_2O/H_2O = 1:9) at 298 K. K_a was given by averaging the values obtained from three independent titrations.

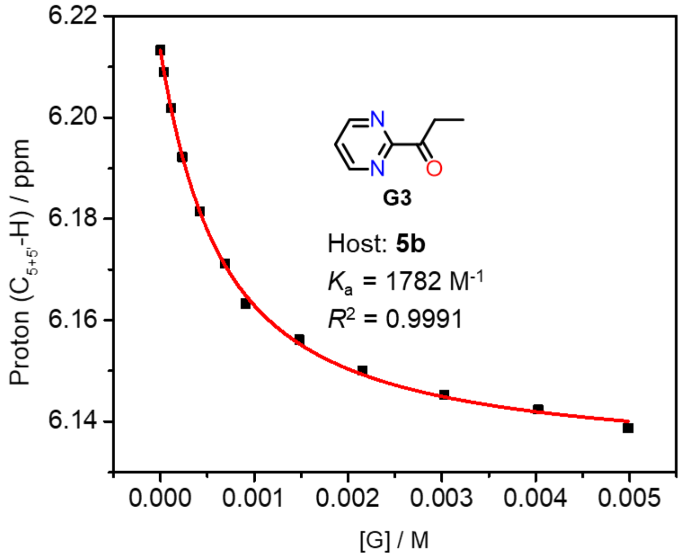

	K_a / M^{-1}	R^2
1st	1782	0.9991
2nd	1929	0.9995
3rd	1703	0.9989
average	$(1.8 \pm 0.1) \times 10^3$	

Supplementary Fig. 61 Non-linear curve-fitting for the complexation between **5b** and **G3** in phosphate buffer (50 mM, pH = 12, D_2O/H_2O = 1:9) at 298 K. K_a was given by averaging the values obtained from three independent titrations.

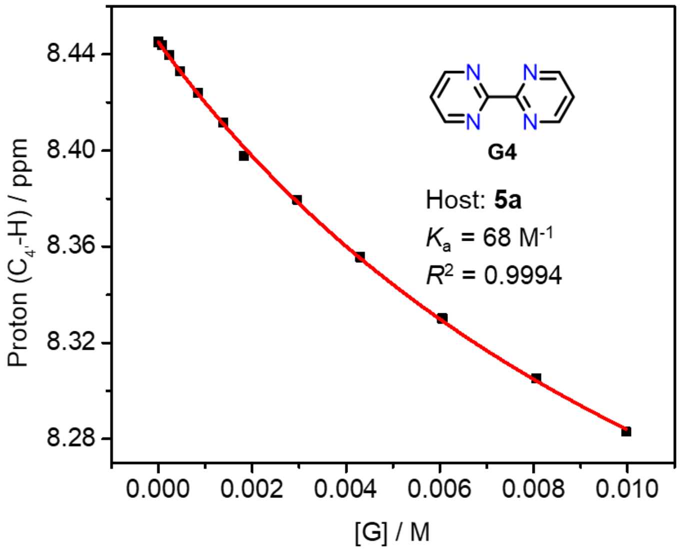

	K_a / M^{-1}	R^2
1st	68	0.9994
2nd	40	0.9993
3rd	53	0.9994
average	$(5 \pm 1) \times 10^1$	

Supplementary Fig. 62 Non-linear curve-fitting for the complexation between **5a** and **G4** in phosphate buffer (50 mM, pH = 12, D_2O/H_2O = 1:9) at 298 K. K_a was given by averaging the values obtained from three independent titrations.

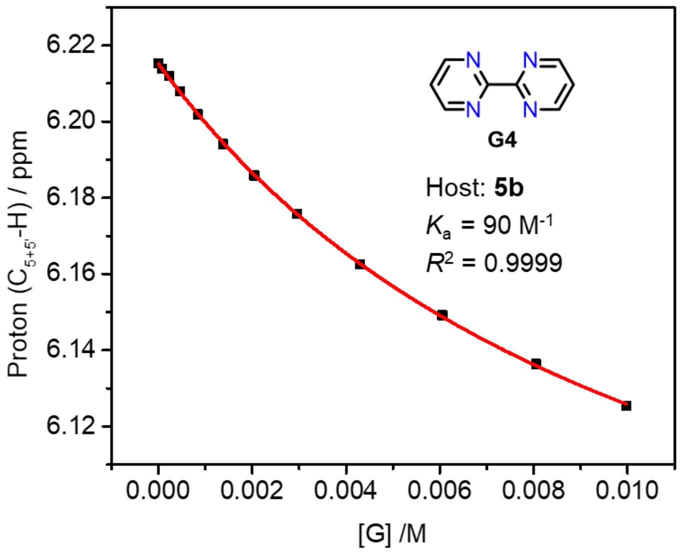

	K_a / M^{-1}	R^2
1st	90	0.9999
2nd	70	0.9994
3rd	106	0.9997
average	$(9 \pm 2) \times 10^1$	

Supplementary Fig. 63 Non-linear curve-fitting for the complexation between **5b** and **G4** in phosphate buffer (50 mM, pH = 12, D_2O/H_2O = 1:9) at 298 K. K_a was given by averaging the values obtained from three independent titrations.

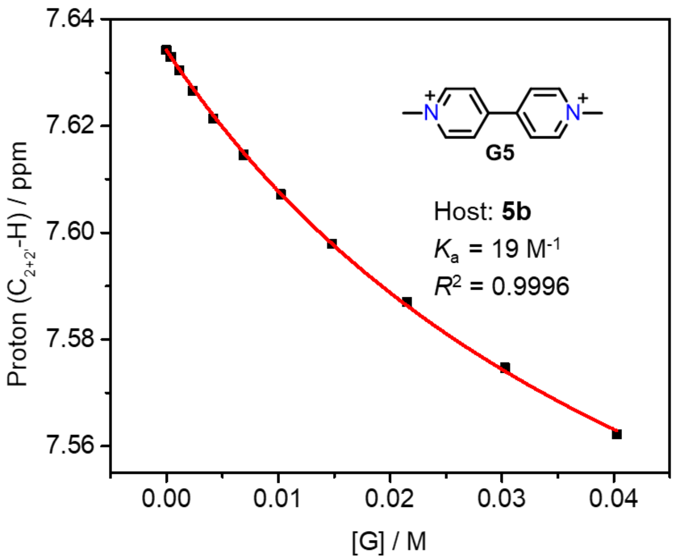

	K_a / M^{-1}	R^2
1st	19	0.9996
2nd	16	0.9988
3rd	26	0.9982
average	$(2.0 \pm 0.4) \times 10^1$	

Supplementary Fig. 64 Non-linear curve-fitting for the complexation between **5b** and **G5** in phosphate buffer (50 mM, pH = 7.4, D_2O/H_2O = 1:9) at 298 K. K_a was given by averaging the values obtained from three independent titrations.

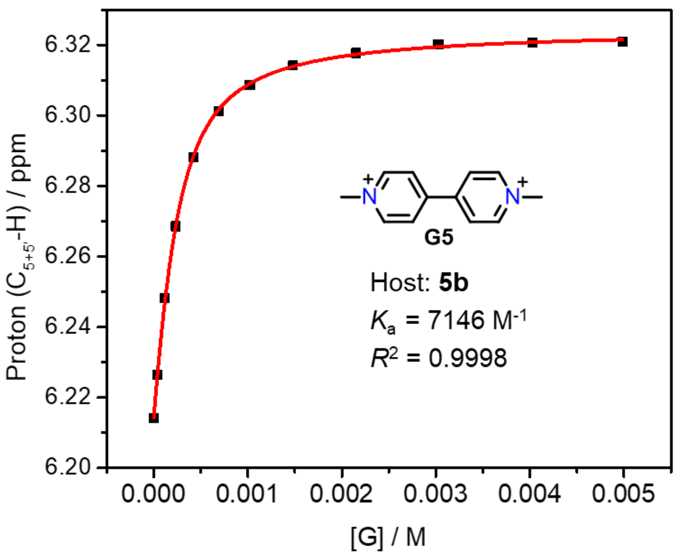

	K_a / M^{-1}	R^2
1st	7146	0.9998
2nd	5414	0.9999
3rd	5219	0.9998
average	$(5.9 \pm 0.9) \times 10^3$	

Supplementary Fig. 65 Non-linear curve-fitting for the complexation between **5b** and **G5** in phosphate buffer (50 mM, pH = 12, D_2O/H_2O = 1:9) at 298 K. K_a was given by averaging the values obtained from three independent titrations.

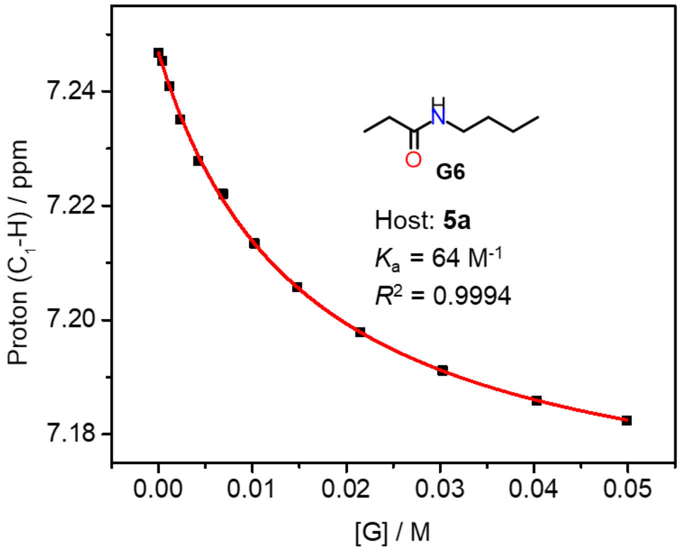

	K_a / M^{-1}	R^2
1st	64	0.9994
2nd	88	0.9990
3rd	66	0.9997
average	$(7 \pm 1) \times 10^1$	

Supplementary Fig. 66 Non-linear curve-fitting for the complexation between **5a** and **G6** in phosphate buffer (50 mM, pH = 7.4, D_2O/H_2O = 1:9) at 298 K. K_a was given by averaging the values obtained from three independent titrations.

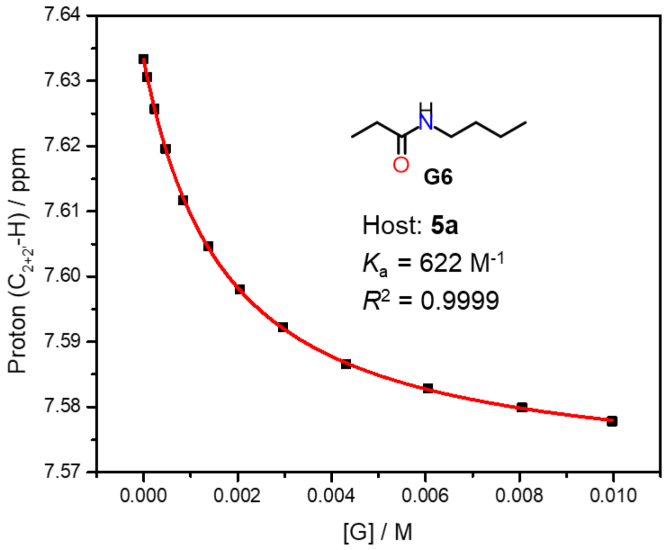

	K_a / M^{-1}	R^2
1st	622	0.9999
2nd	741	0.9997
3rd	747	0.9981
average	$(7.0 \pm 0.6) \times 10^2$	

Supplementary Fig. 67 Non-linear curve-fitting for the complexation between **5a** and **G6** in phosphate buffer (50 mM, pH = 12, D_2O/H_2O = 1:9) at 298 K. K_a was given by averaging the values obtained from three independent titrations.

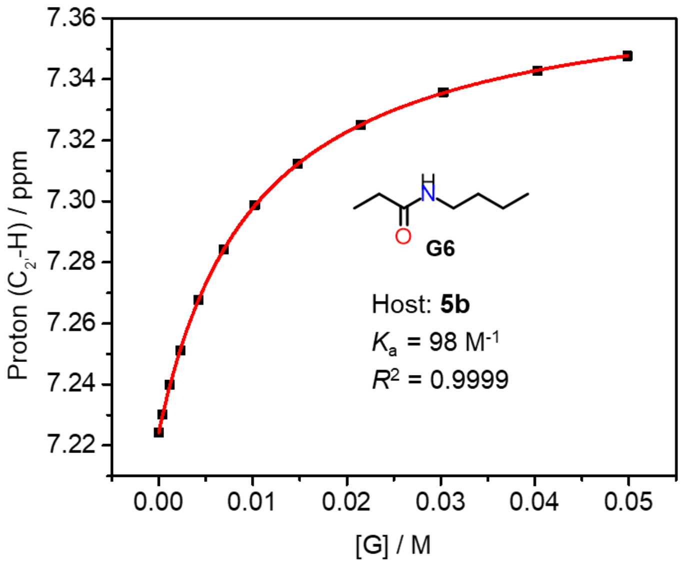

	K_a / M^{-1}	R^2
1st	98	0.9999
2nd	107	0.9997
3rd	96	0.9999
average	$(1.0 \pm 0.1) \times 10^2$	

Supplementary Fig. 68 Non-linear curve-fitting for the complexation between **5b** and **G6** in phosphate buffer (50 mM, pH = 7.4, D_2O/H_2O = 1:9) at 298 K. K_a was given by averaging the values obtained from three independent titrations.

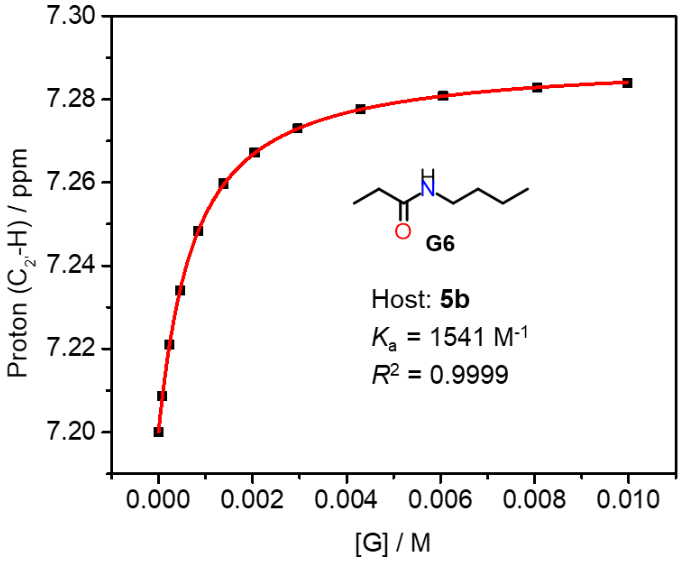

	K_a / M^{-1}	R^2
1st	1541	0.9999
2nd	1689	0.9999
3rd	1725	0.9995
average	$(1.6 \pm 0.1) \times 10^3$	

Supplementary Fig. 69 Non-linear curve-fitting for the complexation between **5b** and **G6** in phosphate buffer (50 mM, pH = 12, D_2O/H_2O = 1:9) at 298 K. K_a was given by averaging the values obtained from three independent titrations.

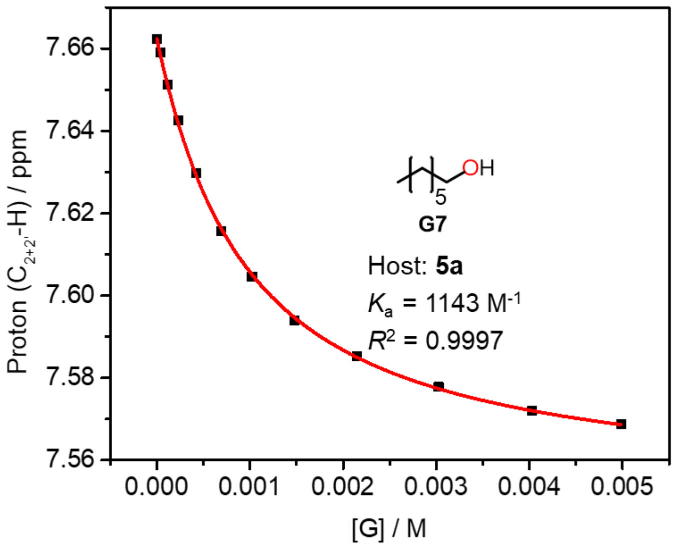

	K_a / M^{-1}	R^2
1st	1143	0.9997
2nd	902	0.9999
3rd	891	0.9998
average	$(1.0 \pm 0.1) \times 10^3$	

Supplementary Fig. 70 Non-linear curve-fitting for the complexation between **5a** and **G7** in phosphate buffer (50 mM, pH = 7.4, D_2O/H_2O = 1:9) at 298 K. K_a was given by averaging the values obtained from three independent titrations.

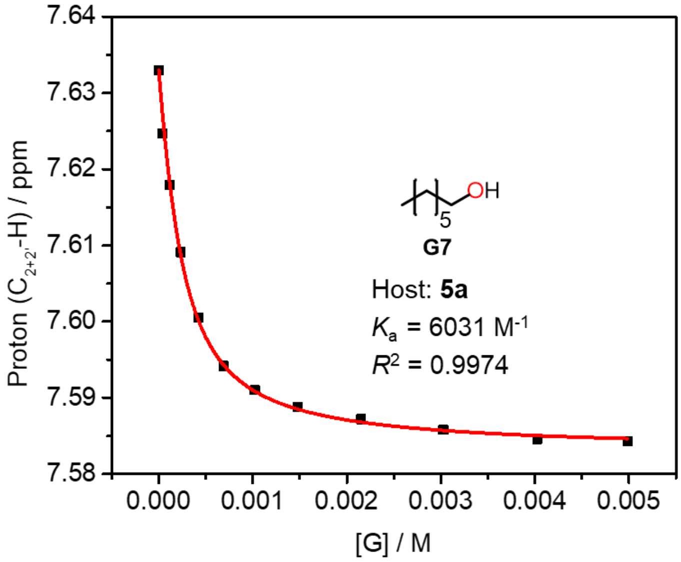

	K_a / M^{-1}	R^2
1st	6031	0.9974
2nd	6236	0.9994
3rd	4583	0.9972
average	$(5.6 \pm 0.7) \times 10^3$	

Supplementary Fig. 71 Non-linear curve-fitting for the complexation between **5a** and **G7** in phosphate buffer (50 mM, pH = 12, D_2O/H_2O = 1:9) at 298 K. K_a was given by averaging the values obtained from three independent titrations.

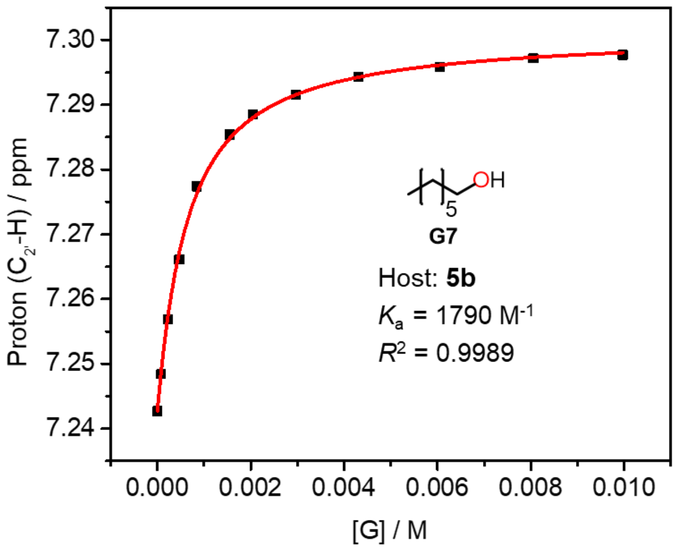

	K_a / M^{-1}	R^2
1st	1790	0.9989
2nd	2637	0.9998
3rd	1669	0.9998
average	$(2.0 \pm 0.4) \times 10^3$	

Supplementary Fig. 72 Non-linear curve-fitting for the complexation between **5b** and **G7** in phosphate buffer (50 mM, pH = 7.4, D_2O/H_2O = 1:9) at 298 K. K_a was given by averaging the values obtained from three independent titrations.

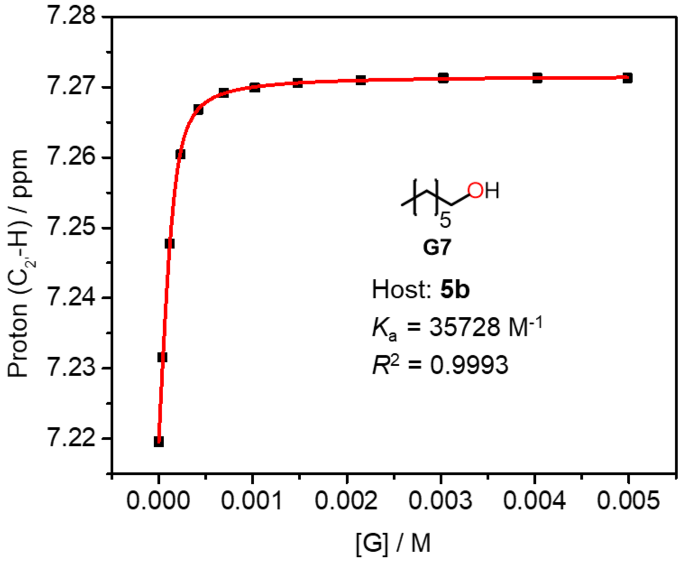

	K_a / M^{-1}	R^2
1st	35728	0.9993
2nd	45076	0.9994
3rd	39229	0.9996
average	$(4.0 \pm 0.4) \times 10^4$	

Supplementary Fig. 73 Non-linear curve-fitting for the complexation between **5b** and **G7** in phosphate buffer (50 mM, pH = 12, D_2O/H_2O = 1:9) at 298 K. K_a was given by averaging the values obtained from three independent titrations.

8. Determination of Association Constants by ITC Titrations

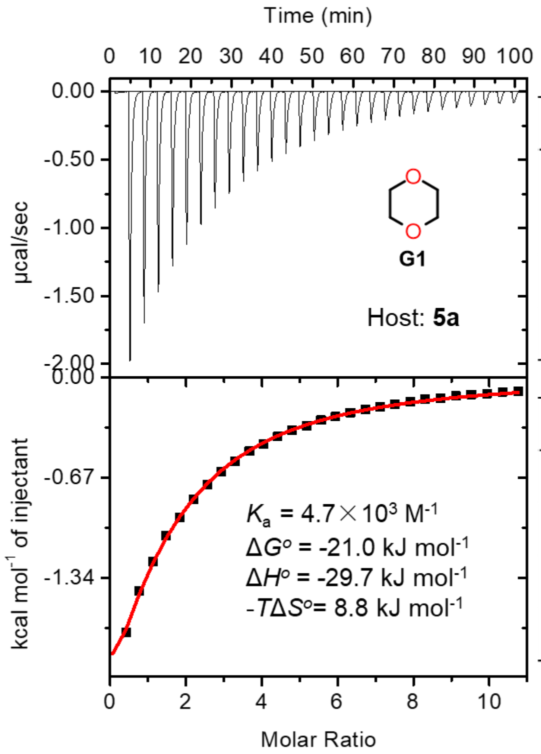

	K_a / M^{-1}	ΔG^o/(kJ mol^{-1})
1st	4.7×10^3	-21.0
2nd	4.4×10^3	-20.8
3rd	4.3×10^3	-20.7
average	$(4.5 \pm 0.2) \times 10^3$	-20.8 ± 0.1

	ΔH^o/(kJ mol^{-1})	$-T\Delta S^o$/(kJ mol^{-1})
1st	-29.7	8.8
2nd	-38.6	17.8
3rd	-37.4	16.7
average	-35 ± 4	14 ± 4

Supplementary Fig. 74 ITC titration of **5a** (0.08 mM, pH = 7.4) with **G1** at 298 K.

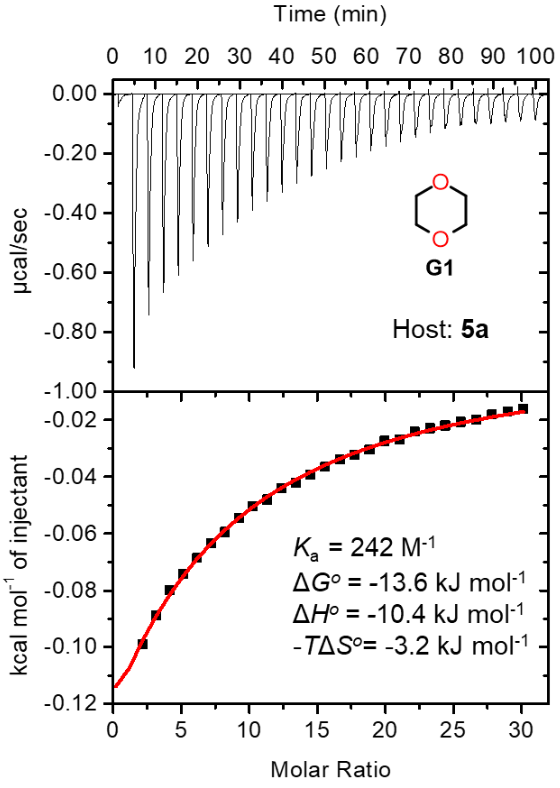

	K_a / M^{-1}	ΔG^o/(kJ mol^{-1})
1st	242	-13.6
2nd	283	-14.0
3rd	229	-13.4
average	$(2.5 \pm 0.2) \times 10^2$	-13.7 ± 0.2

	ΔH^o/(kJ mol^{-1})	$-T\Delta S^o$/(kJ mol^{-1})
1st	-10.4	-3.2
2nd	-10.4	-3.6
3rd	-10.2	-3.2
average	-10.3 ± 0.1	-3.3 ± 0.2

Supplementary Fig. 75 ITC titration of **5a** (0.16 mM, pH = 12) with **G1** at 298 K.

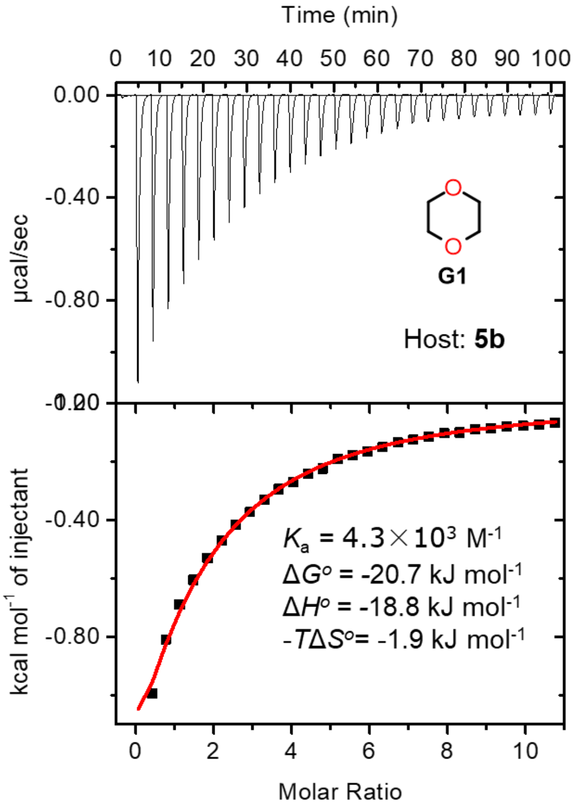

	K_a / M^{-1}	ΔG^o / (kJ mol^{-1})
1st	4.3×10^3	-20.7
2nd	4.5×10^3	-20.9
3rd	4.4×10^3	-20.7
average	$(4.4 \pm 0.1) \times 10^3$	-20.8 ± 0.1

	ΔH^o / (kJ mol^{-1})	$-T\Delta S^o$ / (kJ mol^{-1})
1st	-18.8	-1.9
2nd	-17.0	-3.9
3rd	-19.5	-1.2
average	-18 ± 1	-2 ± 1

Supplementary Fig. 76 ITC titration of **5b** (0.08 mM, pH = 7.4) with **G1** at 298 K.

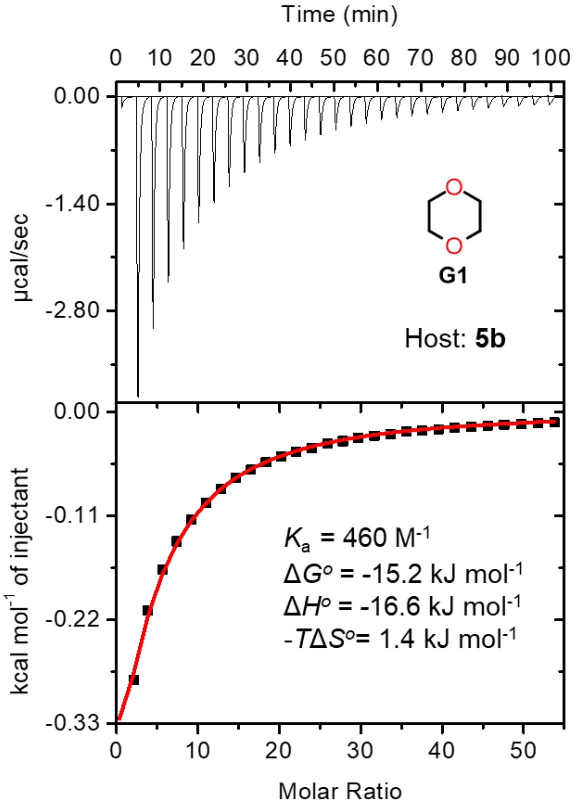

	K_a / M^{-1}	ΔG^o / (kJ mol^{-1})
1st	460	-15.2
2nd	516	-15.4
3rd	514	-15.5
average	$(5.0 \pm 0.3) \times 10^2$	-15.4 ± 0.1

	ΔH^o / (kJ mol^{-1})	$-T\Delta S^o$ / (kJ mol^{-1})
1st	-16.6	1.4
2nd	-12.2	-3.2
3rd	-15.1	-0.4
average	-15 ± 2	-1 ± 2

Supplementary Fig. 77 ITC titration of **5b** (0.16 mM, pH = 12) with **G1** at 298 K.

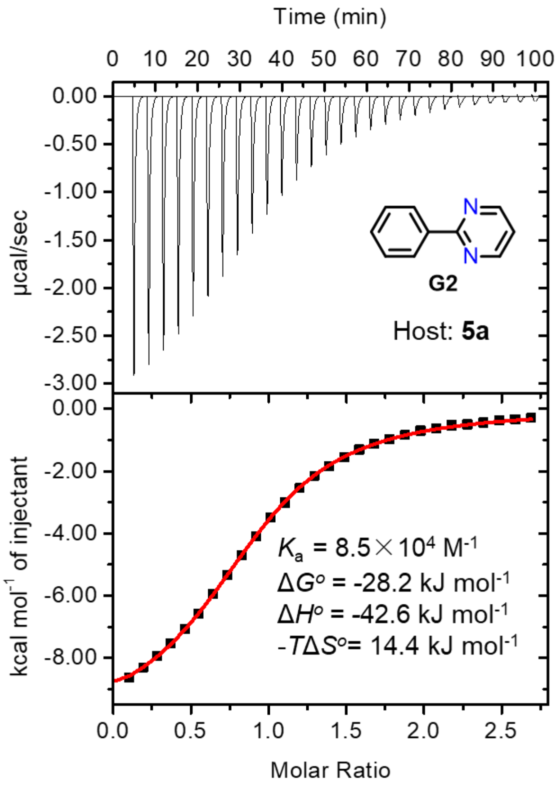

	K_a / M^{-1}	ΔG^o/(kJ mol^{-1})
1st	8.5×10^4	-28.2
2nd	8.2×10^4	-28.1
3rd	8.4×10^4	-28.1
average	$(8.4 \pm 0.1) \times 10^4$	-28.1 ± 0.1

	ΔH^o/(kJ mol^{-1})	$-T\Delta S^o$/(kJ mol^{-1})
1st	-42.6	14.4
2nd	-43.0	14.9
3rd	-41.7	13.6
average	-42.4 ± 0.5	14.3 ± 0.5

Supplementary Fig. 78 ITC titration of **5a** (0.08 mM, pH = 7.4) with **G2** at 298 K.

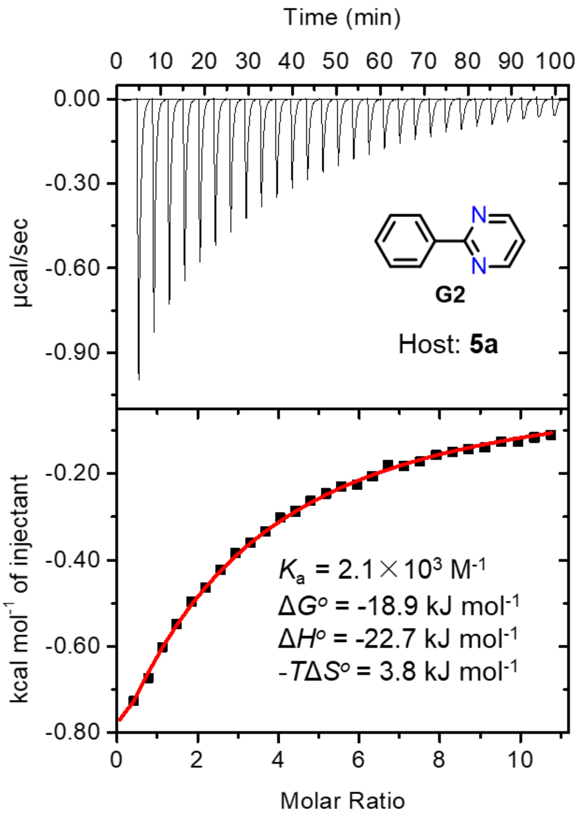

	K_a / M^{-1}	ΔG^o/(kJ mol^{-1})
1st	2.1×10^3	-18.9
2nd	2.9×10^3	-19.7
3rd	2.1×10^3	-19.0
average	$(2.4 \pm 0.4) \times 10^3$	-19.2 ± 0.4

	ΔH^o/(kJ mol^{-1})	$-T\Delta S^o$/(kJ mol^{-1})
1st	-22.7	3.8
2nd	-19.4	-0.3
3rd	-22.8	3.8
average	-22 ± 2	2 ± 2

Supplementary Fig. 79 ITC titration of **5a** (0.08 mM, pH =12) with **G2** at 298 K.

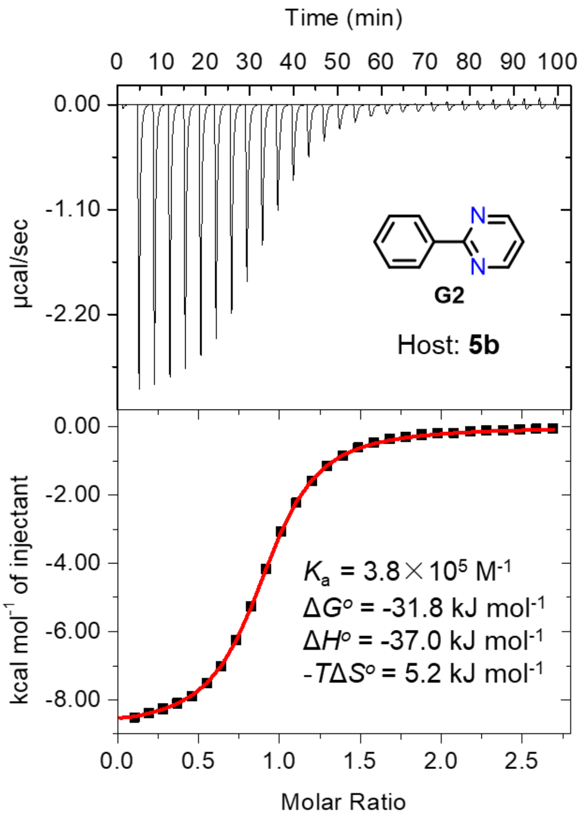

	K_a / M^{-1}	ΔG^o/(kJ mol^{-1})
1st	3.8 × 10^5	-31.8
2nd	3.7 × 10^5	-31.7
3rd	3.7 × 10^5	-31.8
average	(3.7 ± 0.1) × 10^5	-31.8 ± 0.1

	ΔH^o/(kJ mol^{-1})	-$T\Delta S^o$/(kJ mol^{-1})
1st	-37.0	5.2
2nd	-34.7	3.0
3rd	-36.3	4.5
average	-36 ± 1	4.2± 0.9

Supplementary Fig. 80 ITC titration of **5b** (0.08 mM, pH =7.4) with **G2** at 298 K.

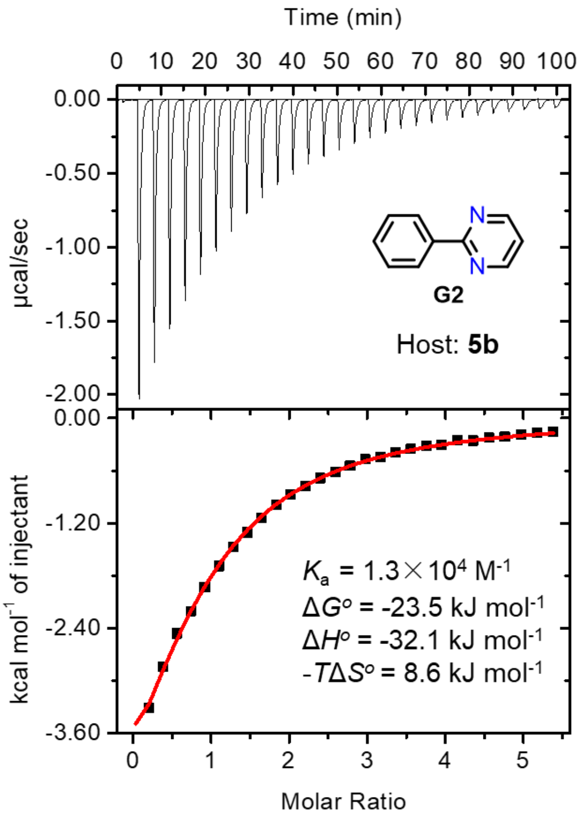

	K_a / M^{-1}	ΔG^o/(kJ mol^{-1})
1st	1.3 × 10^4	-23.5
2nd	1.5 × 10^4	-23.8
3rd	1.7 × 10^4	-24.0
average	(1.5 ± 0.2) × 10^4	-23.8 ± 0.2

	ΔH^o/(kJ mol^{-1})	-$T\Delta S^o$/(kJ mol^{-1})
1st	-32.1	8.6
2nd	-32.1	8.3
3rd	-26.8	2.8
average	-30 ± 3	7± 3

Supplementary Fig. 81 ITC titration of **5b** (0.08 mM, pH =12) with **G2** at 298 K.

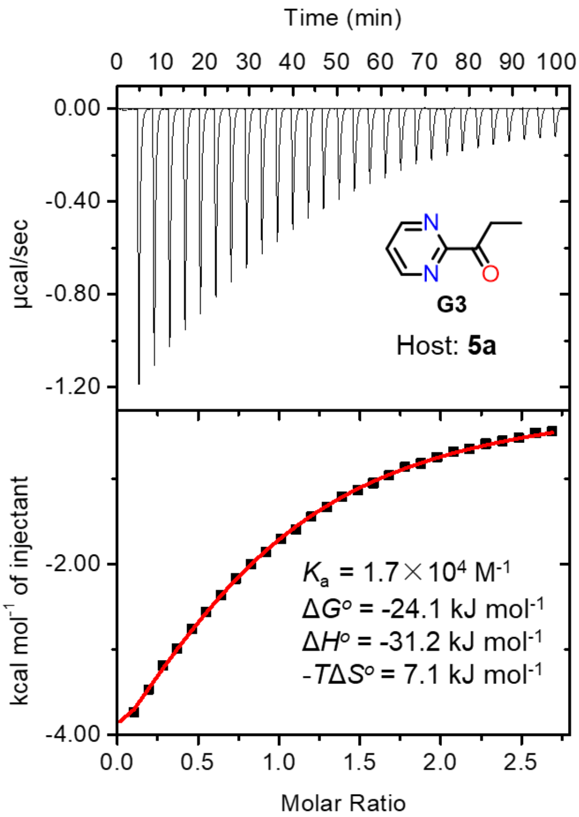

	K_a / M^{-1}	ΔG^o / (kJ mol^{-1})
1st	1.7 × 10^4	-24.1
2nd	2.0 × 10^4	-24.6
3rd	2.3 × 10^4	-25.0
average	(2.0 ± 0.2) × 10^4	-24.6 ± 0.4

	ΔH^o / (kJ mol^{-1})	$-T\Delta S^o$ / (kJ mol^{-1})
1st	-31.2	7.1
2nd	-27.9	3.3
3rd	-28.2	3.2
average	-29 ± 2	5 ± 2

Supplementary Fig. 82 ITC titration of **5a** (0.08 mM, pH = 7.4) with **G3** at 298 K.

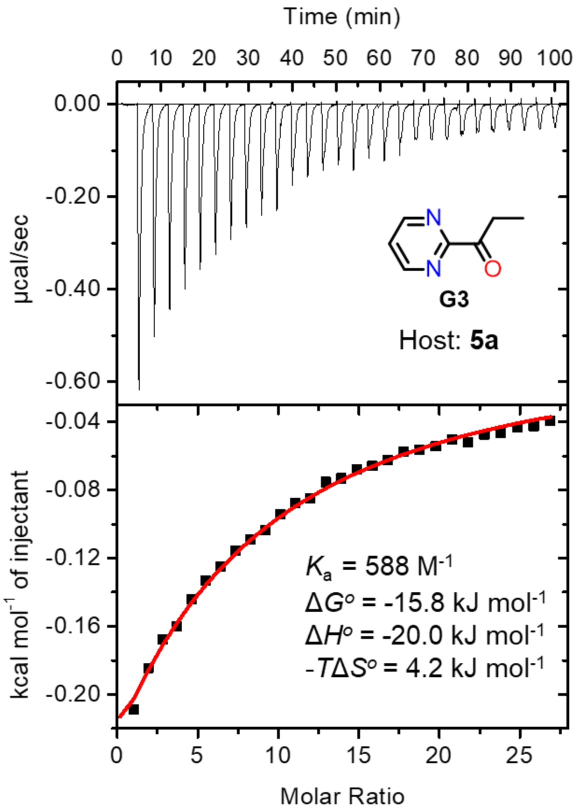

	K_a / M^{-1}	ΔG^o / (kJ mol^{-1})
1st	588	-15.8
2nd	542	-15.6
3rd	617	-15.9
average	(5.8 ± 0.3) × 10^2	-15.8 ± 0.1

	ΔH^o / (kJ mol^{-1})	$-T\Delta S^o$ / (kJ mol^{-1})
1st	-20.0	4.2
2nd	-23.0	7.4
3rd	-18.3	2.4
average	-20 ± 2	5 ± 2

Supplementary Fig. 83 ITC titration of **5a** (0.08 mM, pH = 12) with **G3** at 298 K.

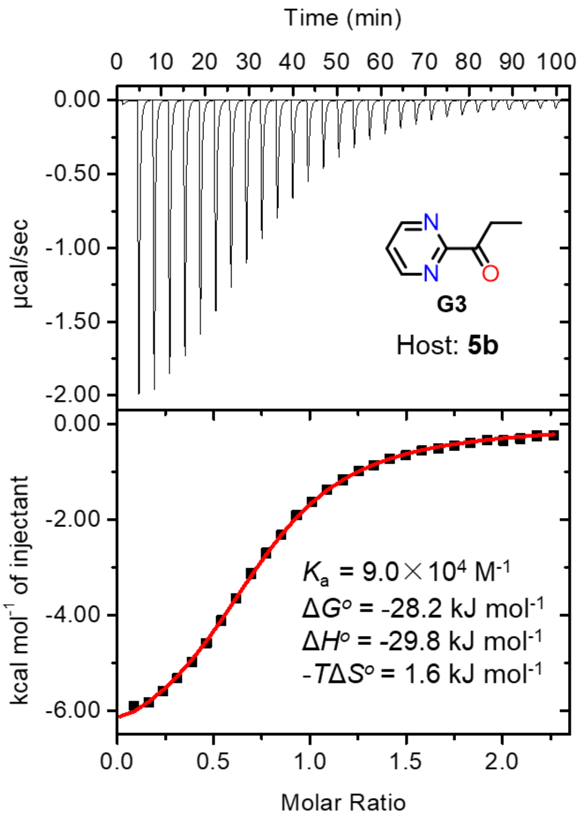

	K_a / M^{-1}	ΔG^o /(kJ mol^{-1})
1st	9.0×10^4	-28.2
2nd	9.5×10^4	-28.3
3rd	9.8×10^4	-28.4
average	$(9.4 \pm 0.3) \times 10^4$	-28.3 ± 0.1

	ΔH^o /(kJ mol^{-1})	$-T\Delta S^o$ /(kJ mol^{-1})
1st	-29.8	1.6
2nd	-26.7	-1.6
3rd	-26.4	-2.0
average	-28 ± 2	-1 ± 2

Supplementary Fig. 84 ITC titration of **5b** (0.08 mM, pH = 7.4) with **G3** at 298 K.

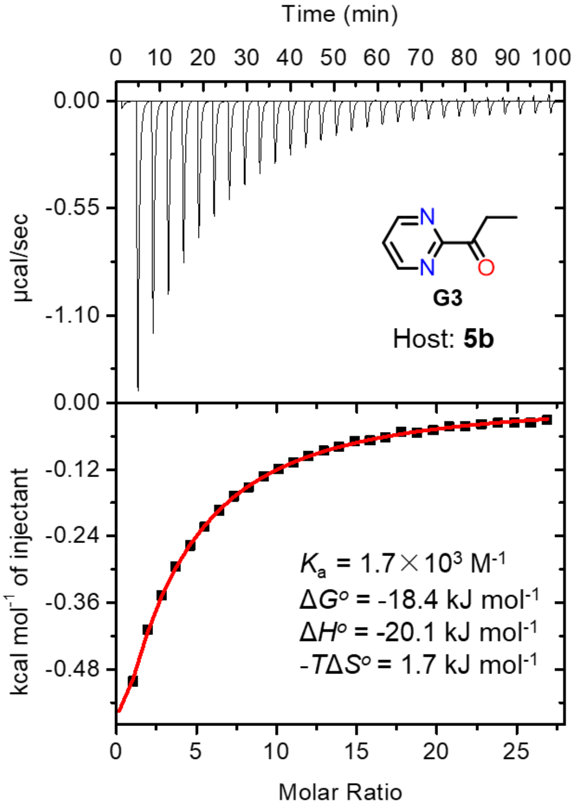

	K_a / M^{-1}	ΔG^o /(kJ mol^{-1})
1st	1.7×10^3	-18.4
2nd	$1,6 \times 10^3$	-18.2
3rd	$1,7 \times 10^3$	-18.5
average	$(1.7 \pm 0.1) \times 10^3$	-18.4 ± 0.1

	ΔH^o /(kJ mol^{-1})	$-T\Delta S^o$ /(kJ mol^{-1})
1st	-20.1	1.7
2nd	-17.1	-1.1
3rd	-19.3	0.8
average	-19 ± 1	1 ± 1

Supplementary Fig. 85 ITC titration of **5b** (0.08 mM, pH = 12) with **G3** at 298 K.

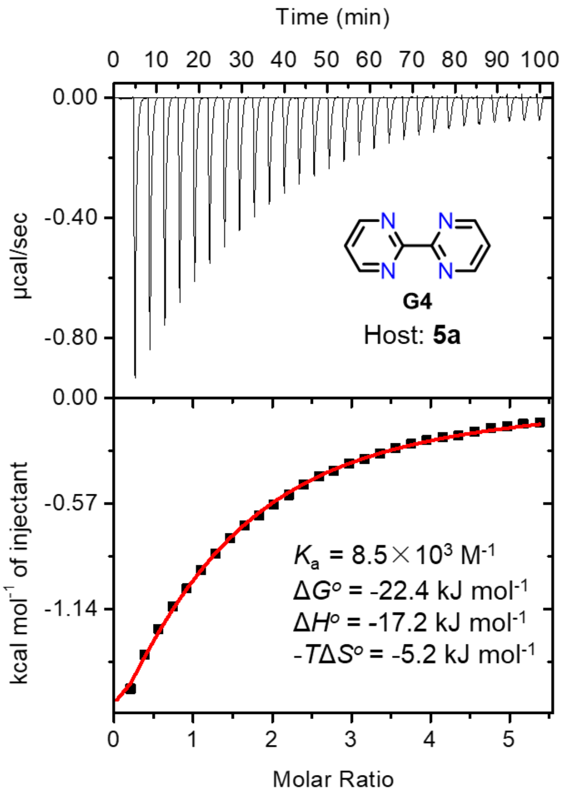

	K_a / M^{-1}	ΔG^o / (kJ mol^{-1})
1st	8.5×10^3	-22.4
2nd	8.9×10^3	-22.5
3rd	8.5×10^3	-22.4
average	$(8.6 \pm 0.2) \times 10^3$	-22.4 ± 0.1

	ΔH^o / (kJ mol^{-1})	$-T\Delta S^o$ / (kJ mol^{-1})
1st	-17.2	-5.2
2nd	-17.1	-5.4
3rd	-18.9	-3.5
average	-17.7 ± 0.8	-4.7 ± 0.9

Supplementary Fig. 86 ITC titration of **5a** (0.08 mM, pH = 7.4) with **G4** at 298 K.

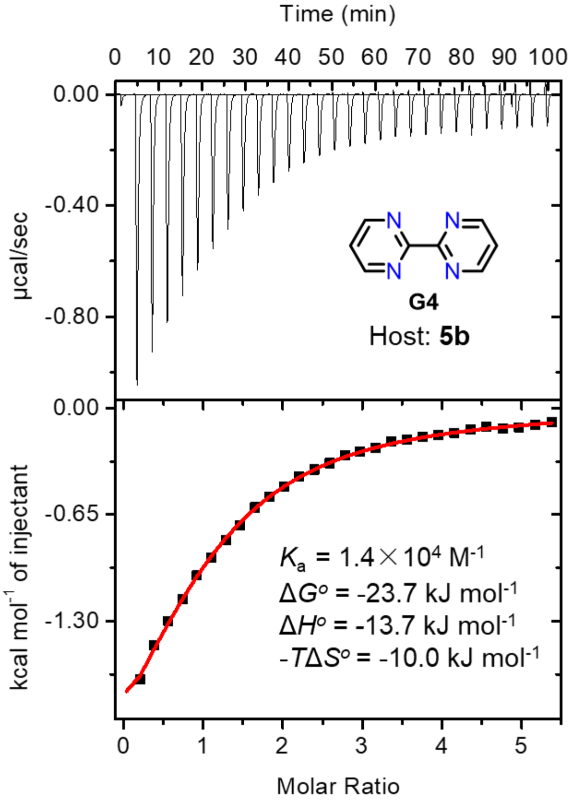

	K_a / M^{-1}	ΔG^o / (kJ mol^{-1})
1st	1.4×10^4	-23.7
2nd	1.7×10^4	-24.0
3rd	1.9×10^4	-24.4
average	$(1.7 \pm 0.2) \times 10^4$	-24.0 ± 0.3

	ΔH^o / (kJ mol^{-1})	$-T\Delta S^o$ / (kJ mol^{-1})
1st	-13.7	-10.0
2nd	-12.8	-11.2
3rd	-12.0	-12.4
average	-12.8 ± 0.7	-11 ± 1

Supplementary Fig. 87 ITC titration of **5b** (0.08 mM, pH = 7.4) with **G4** at 298 K

9. Association Constants of 5 with Tetramethylammonium

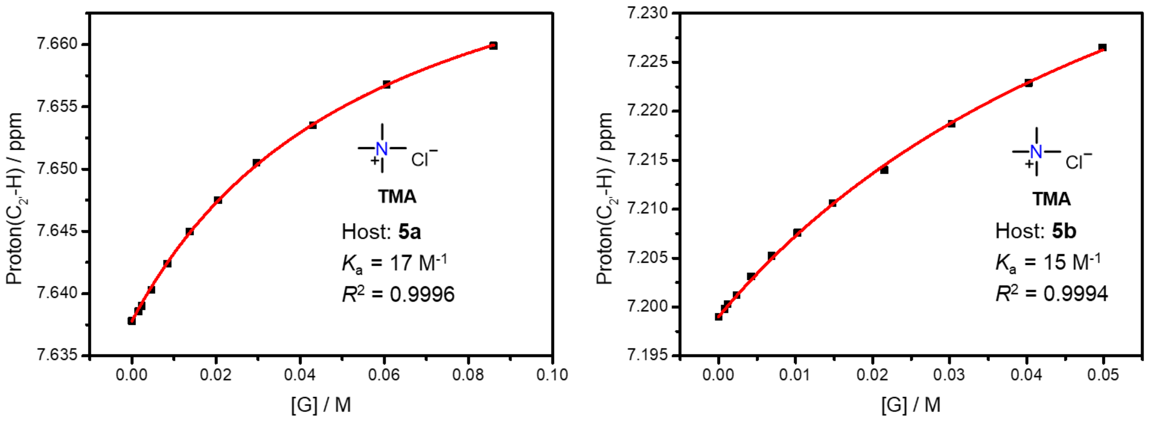

Supplementary Fig. 88 Non-linear curve-fitting for the complexation between **5** and tetramethylammonium (**TMA**) in phosphate buffer (50 mM, pH = 12, D_2O/H_2O = 1:9) at 298 K. **TMA** shows very weak binding to **5**, which may be due to unmatched shape and size, as these guests are spherical and the naphthotubes are tubular and narrow in size.

10. Data for Switchable Bifunctional Molecular Recognition

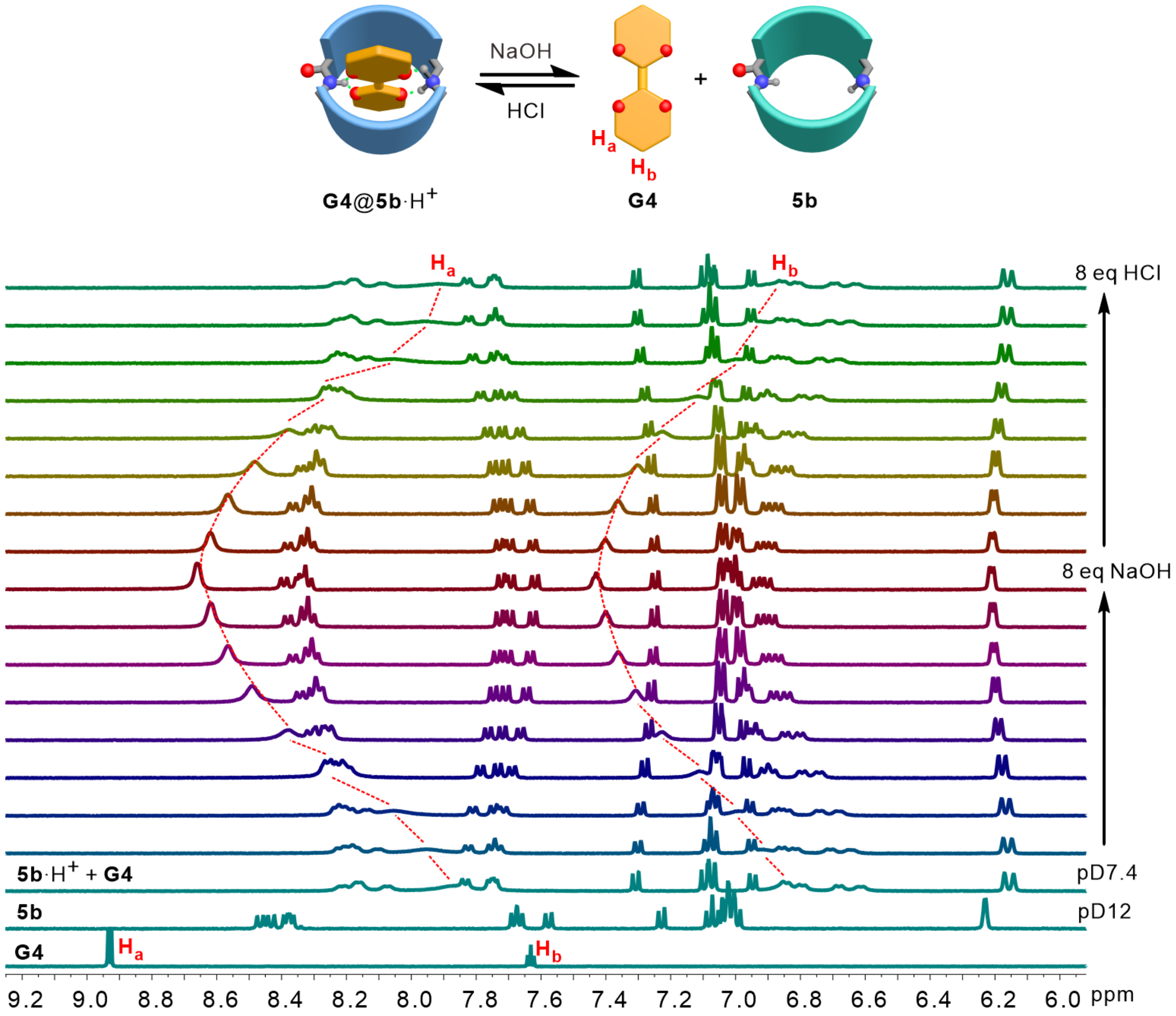

Supplementary Fig. 89 Cartoon representation of switchable molecular recognition and partial ^{1}H NMR spectra (500 MHz, D_2O) of the equimolar mixture of **5b** and **G4** in *non*-buffered D_2O at pD 7.4 after gradually adding 8 equivalents of NaOH and then 8 equivalents of HCl. As shown in the Figure, protons H_a and H_b of **G4** undergoes downfield shift closed to the position of free **G4** throught gradually adding 8 eq of NaOH. At this point in time, the ^{1}H NMR spectra is the superposition of free **G4** and free but deprotonated **5b** at pD 12. This suggests that **G4** is released from the cavity of **5b** when the cavity is deprotonated after adding NaOH. The ^{1}H NMR spectra can be restored after adding 8 equivalents of HCl. This indicates that **G4** can be uptaken again into the cavity of **5b** after protonation.

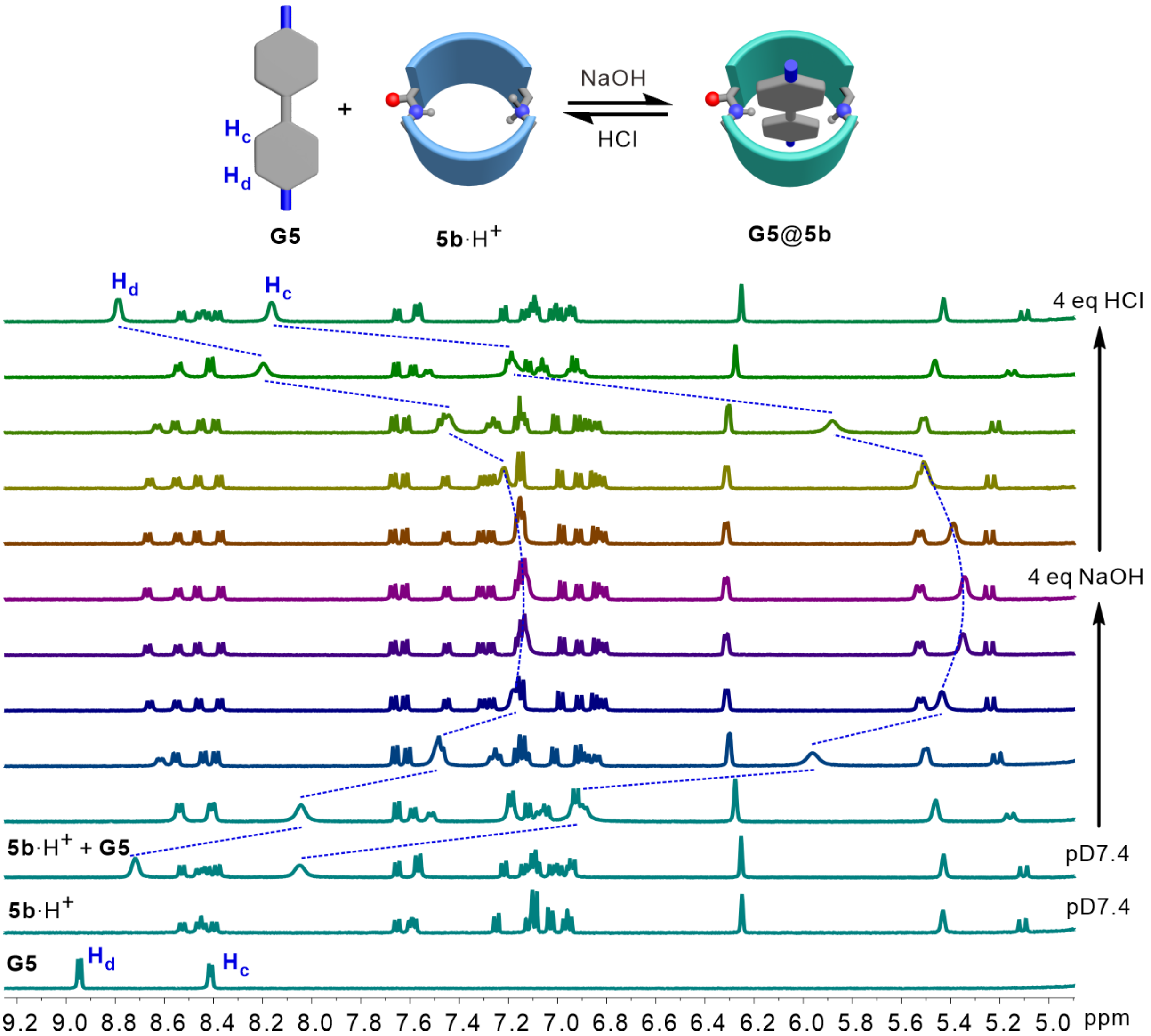

Supplementary Fig. 90 Cartoon representation of switchable molecular recognition and partial ^{1}H NMR spectra (500 MHz, D_2O) of the equimolar mixture of **5b** and **G5** in *non*-buffered D_2O at pD 7.4 after gradually adding 4 equivalents of NaOH and then 4 equivalents of HCl. As shown in the Figure, the ^{1}H NMR spectra is the superposition of free **G5** and free but protonated **5b** at pD 7.4. Protons H_c and H_d of **G5** undergoes upfield shift from close position of free **G5** througth gradually adding 4 eq of NaOH. This suggests that **G5** is uptaken by the cavity of **5b** from solution when the cavity is deprotonated after adding NaOH. The ^{1}H NMR spectra can be restored after adding 4 equivalents of HCl. This indicates that **G5** can be released again from the cavity of **5b** after protonation into solution.

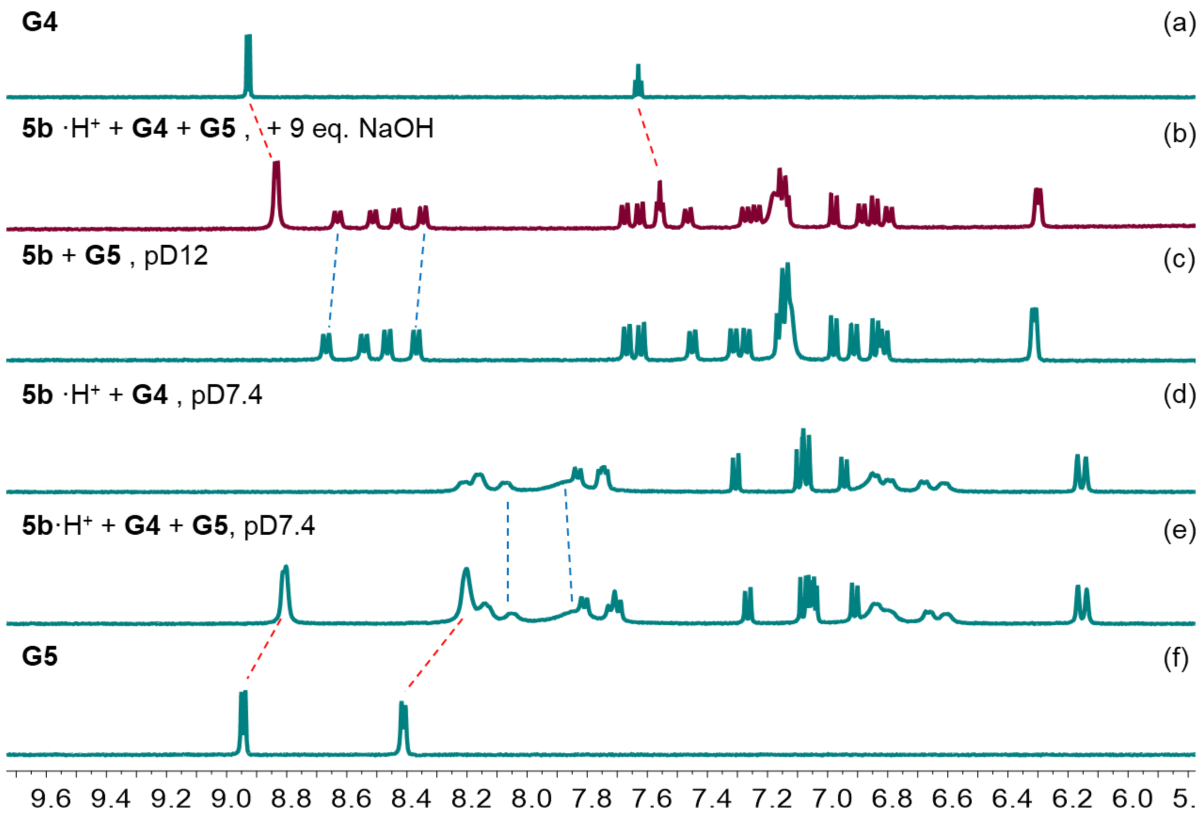

Supplementary Fig. 91 Partial ^{1}H NMR spectra (500 MHz, D_2O, 298 K) of free guests (a) **G4**, (f) **G5**, (c) the equimolar mixture of **5b**, and **G5** in *non*-buffered D_2O at pD 12; (d) the equimolar mixture of **5b**, and **G4** in *non*-buffered D_2O at pD 7.4; (e) the equimolar mixture of **5b**, **G4** and **G5** in *non*-buffered D_2O at pD 7.4 and (b) after gradually adding 9 equivalents of NaOH. As shown in the Figure, in the ternary mixture of **5b**, **G4** and **G5,** the ^{1}H NMR spectra is the superposition of free **G5** and the complex of protonated **5b** and **G4** at pD 7.4. After gradually 9 equivalents of NaOH, the ^{1}H NMR spectra is the superposition of the spectra of free **G4** and the complex of **5b** and **G5**. This suggests **5b** is able to perform switchable bifunctional molecular recognition in the ternary mixture of **5b**, **G4** and **G5** through adjusting the pH value of the solution.

11. X-Ray Crystallography

Suitable single crystals of **4a**•H^+•3.5CH_3OH•PF_6^- for structural determination were grown through slow diffusion of CH_3OH to the CH_2Cl_2 solution of **4a** H^+•PF_6^- in a sample bottle for a week.

Suitable single crystals of **4b**•H^+•PF_6^- for structural determination were obtained by slow volatilization of its CH_2Cl_2 solution in a sample bottle for 4 days.

Suitable single crystals of **G4@4b**•H^+•PF_6^- for structural determination were obtained by slow volatilization of their CH_2Cl_2 solution in a sample bottle for a week.

All single crystal X-ray data were collected on a Bruker D8 VENTURE with Cu Kα radiation (λ = 1.54178 Å) at 100 K. The structures were solved by intrinsic phasing methods (SHELXT[7]) and refined by full-matrix least squares on F^2 using SHELXL[8] in the OLEX2 program package.[9] All non-hydrogen atoms were refined with anisotropic thermal parameters and the hydrogen atoms carbon atoms were fixed at calculated positions and refined by a riding mode. All hydrogen atoms attached to oxygen and nitrogen are located from the electron density maps except the hydrogen atoms on N1 in **G4@4b**•H^+•PF_6^- (NH and NH2 disordered in the same position and refinement based on electron density map is unstable). SQUEEZE routine implemented on PLATON[10] was used to remove electron densities corresponding to disordered solvent molecules in the crystal data. In **4a**•H^+•3.5CH_3OH•PF6$^-$, part of the OCH_2CH_3 group is replaced by OCH_3 group, which is attributed to the reaction of ester with methanol. The structure of **G4@4b**•H^+•PF6$^-$ does not perfectly fits the *C2/c* space group due to the O4 atom unambiguously located on one side of the molecule, however, the coordinations and the thermal parameters of other atoms are highly symmetry-ralated. Therefore, we think it is more reasonable to analyze the current structure in *C2/c* space group. The occupancy of O4 was set to 0.5.

Supplementary Table 4 Crystal data and structure refinement for **4a**•H^+•3.5CH_3OH•PF_6^- and **4b**•H^+•PF_6^-

entry	**4a**•H^+•3.5CH_3OH•PF_6^-	**4b**•H^+•PF_6^-
Moiety formula	$C_{64.5}H_{58.5}N_2O_{17}$, 3.5($CH_4O$), HPF_6	$C_{66}H_{60}N_2O_{17}$, HPF_6
Empirical formula	$C_{69.15}H_{77.5}F_6N_2O_{21.5}P$	$C_{66}H_{61}N_2O_{17}F_6P$
Formula weight	1425.60	1299.13
Temperature/K	100	100
Crystal system	monoclinic	monoclinic
Space group	$P\ 2_1/n$	$P\ 2_1/n$
a/Å	16.0903(5)	15.8157(19)
b/Å	16.8570(5)	23.535(3)
c/Å	25.6383(8)	17.464(2)
α/°	90	90
β/°	107.806(2)	92.917(8)
γ/°	90	90
Volume/Å^3	6620.9(4)	6492.1(13)
Z	4	4
ρ_{calc}g/cm^3	1.430	1.329
μ/mm^{-1}	1.209	1.131
F(000)	2990.0	2704.0
Reflections collected	75728	26729
Independent reflections	11666 [R_{int} = 0.0545, R_{sigma} = 0.0322]	8675[R_{int} = 0.0915, R_{sigma} = 0.0968]
Data/restraints/parameters	11666/313/986	8675/43/850
Goodness-of-fit on F^2	1.025	1.040
Final R indexes [I>=2σ (I)]	R_1 = 0.0961, wR_2 = 0.2815	R_1 = 0.0797, wR_2 = 0.2040
Final R indexes [all data]	R_1 = 0.1148, wR_2 = 0.3009	R_1 = 0.1262, wR_2 = 0.2324
CCDC number	2106751	2106752

Validation Reply From: response to alert in the structure solution of **4b**•H^+•PF_6^-

Problem: THETM01_ALERT_3_A

The value of sine(theta_max)/wavelength is less than 0.550

Calculated sin(theta_max)/wavelength = 0.5434

Response: The intensity of reflections at high 2theta angle is too weak to be observed, and the data was cut off at a resolution of 0.92 angstrom.

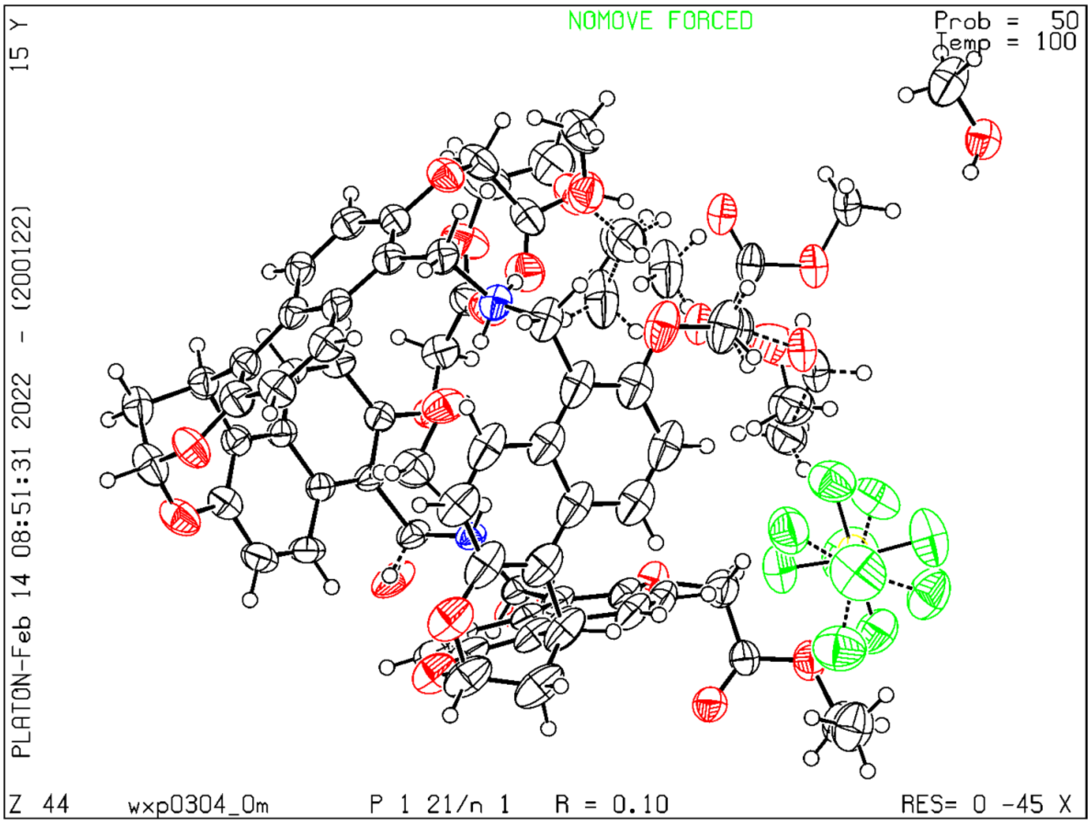

Supplementary Fig. 92 Crystal structure of **4a**•H$^+$•3.5CH_3OH•PF_6^-

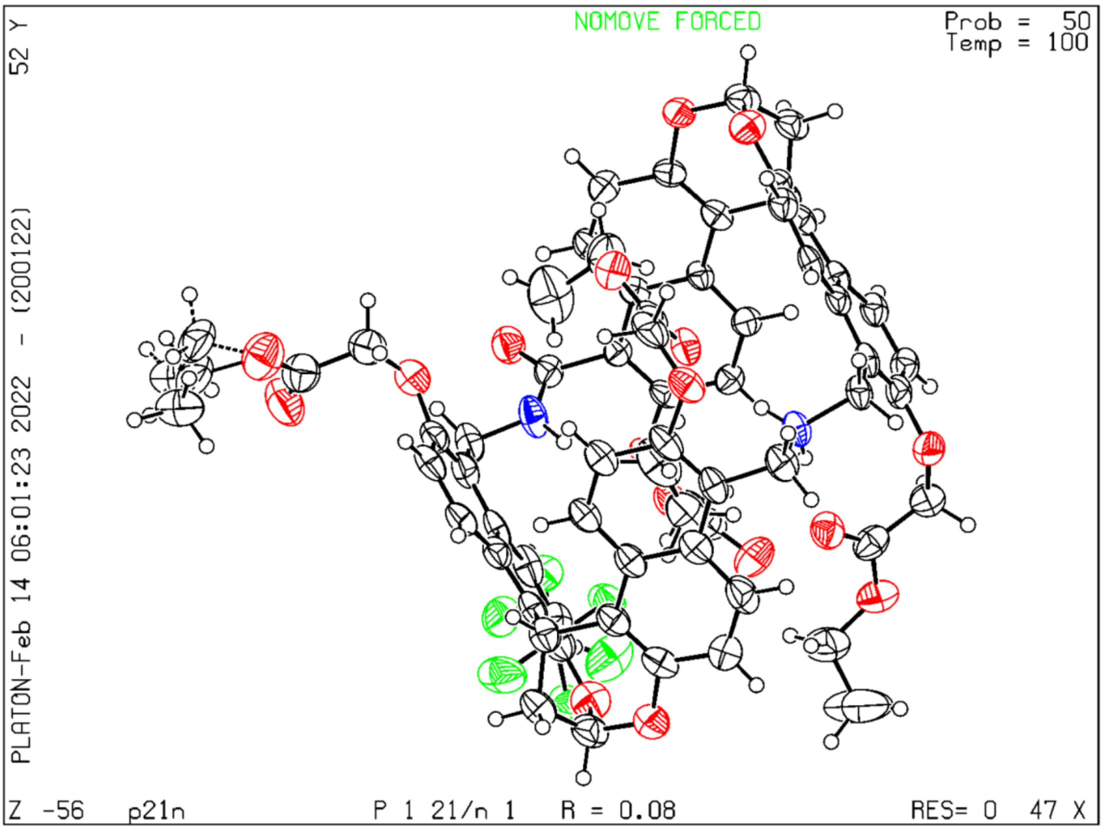

Supplementary Fig. 93 Crystal structure of **4b**•H$^+$•PF_6^-

Supplementary Table 5 Crystal data and structure refinement for **G4@4b**•H$^+$•PF$_6^-$

entry	**G4@4b**•H$^+$•PF$_6^-$
Moiety formula	$C_{66}H_{60}N_2O_{17}$, $C_8H_6N_4$, HPF_6
Empirical formula	$C_{74}H_{67}F_6N_6O_{17}P$
Formula weight	1455.29
Temperature/K	100
Crystal system	monoclinic
Space group	*C2/c*
a/Å	24.5159(8)
b/Å	21.9058(7)
c/Å	13.4984(4)
α/°	90
β/°	100.035(2)
γ/°	90
Volume/Å^3	7138.3(4)
Z	4
ρ_{calc}g/cm^3	1.354
μ/mm^{-1}	1.108
F(000)	1408
Reflections collected	3024.0
Independent reflections	6395 [R_{int} = 0.0526, R_{sigma} = 0.0329]
Data/restraints/parameters	6395/372/587
Goodness-of-fit on F^2	1.000
Final R indexes [I>=2σ (I)]	R_1 = 0.1036, wR_2 = 0.2780
Final R indexes [all data]	R_1 = 0.1186, wR_2 = 0.2908
CCDC number	2106753

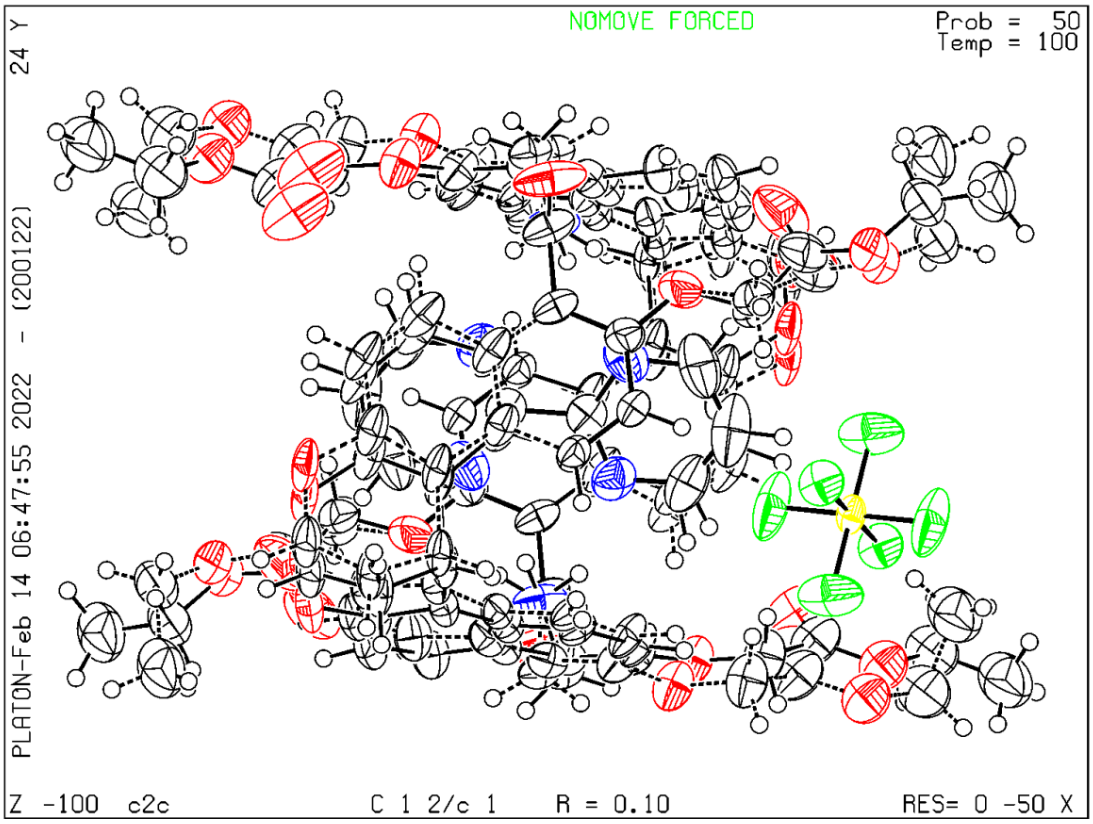

Supplementary Fig. 94 Crystal structure of **G4@4b**•H$^+$•PF$_6^-$

12 Synthetic Procedures

12.1 Synthesis of 3a and 3b

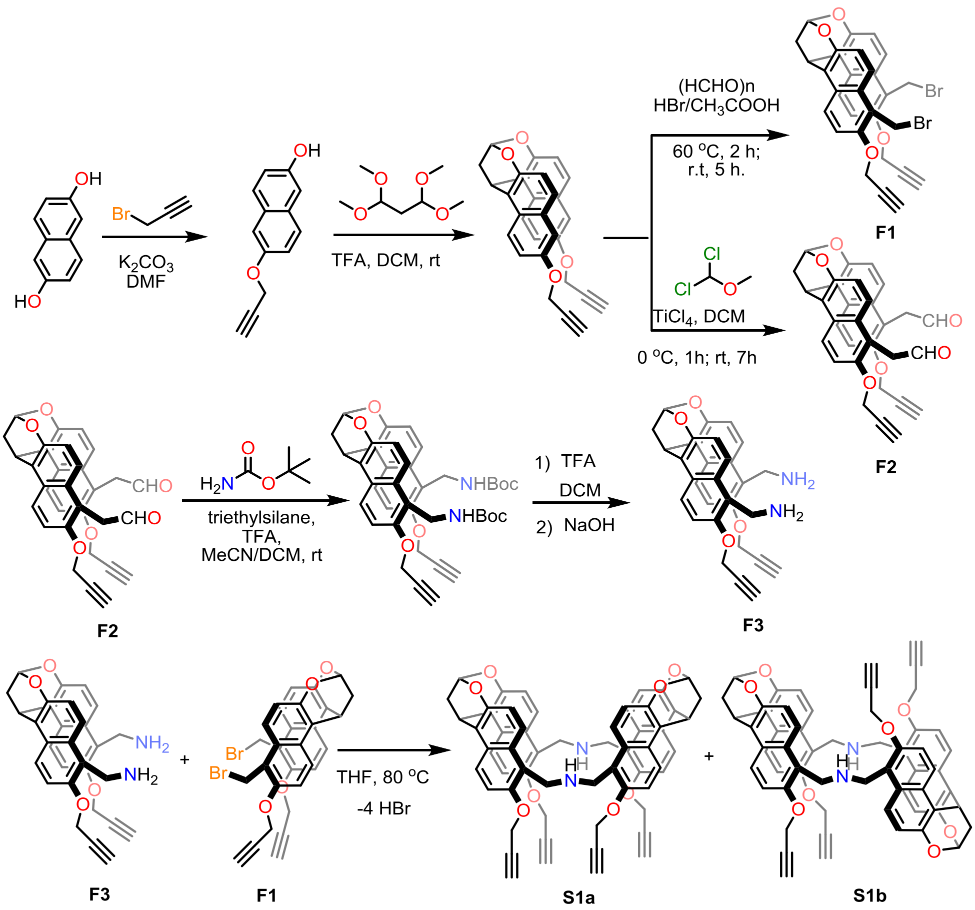

Supplementary Fig. 95 Synthetic procedures of **S1a** and **S1b**.[1]

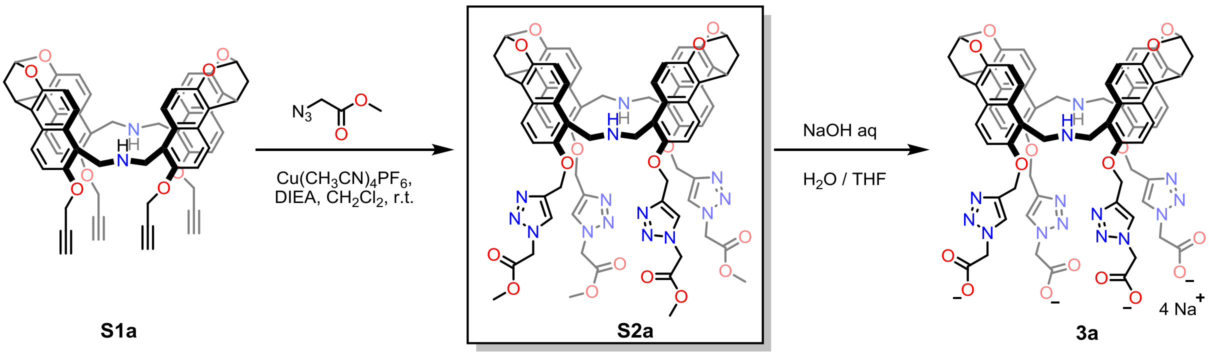

Supplementary Fig. 96 Synthetic procedures of **3a**

Compound 3a. **S1a** (95 mg, 0.1 mmol), Methyl 2-azidoacetate (184 mg, 16 eq.), tetrakis(acetonitrile)copper(I) hexafluorophosphate (20 mg) and ethyldiisopropylamine (200 μL) were added to the solvent CH_2Cl_2 (300 mL) in a 500 mL two-necked flask at room

temperature. Then the resulting mixture was stirred for another 24 h at 45 °C. All volatiles were removed under reduced pressure and the residue was poured into 20% sodium edetate water solution (50 mL), stirred for 30 min, and then filtered. The filter cake was washed by water to afford the crude product **S2a** which was used directly in the next step.

Crude product **S2a** was obtained from the above reaction, was loaded into a 250 mL one-neck flask charged with a magnetic stirring bar. MeOH (100 mL), H_2O (20 mL) and THF (100 mL) were added into the flask. Then the NaOH (80 mg, 2 mmol) was added. The mixture was stirred at room temperature. After 12 h, all volatiles were removed under reduced pressure. The residue was dissolved in H_2O (10 mL), and 3 N HCl were added dropwise to the solution to adjust the pH value to be 2-3. The mixture was filtered and washed by water extensively to get off-white solid (110 mg). The solid (110 mg) was loaded into a 250 mL one-neck flask, MeOH (10 mL) and H_2O (1 mL) was added into the flask. NaOH (48 mg, 1.2 mmol) was then added. The precipitate was then collected through filtration and dried to afford pure **3a** as a white solid (80 mg, yield 60% based on **S1a**).

3a, White solid, m.p. > 200 °C (decomposed); ^{1}H NMR (500 MHz, D_2O/MeOD = 1/4, 298 K) δ [ppm] = 8.44 (d, *J* = 9.5 Hz, 4H), 8.00 (s, 4H), 7.63 (d, *J* = 9.3 Hz, 4H), 7.37 (d, *J* = 9.5 Hz, 4H), 7.05 (d, *J* = 9.3 Hz, 4H), 6.27 (s, 2H), 5.47 (s, 2H), 5.29 – 5.21 (m, 8H), 5.06 – 4.93 (m, 8H), 4.22 (d, *J* = 11.5 Hz, 4H), 4.03 (d, *J* = 11.5 Hz, 4H), 2.61 (s, 4H); ^{13}C NMR (126 MHz, D_2O/MeOD = 1/4, 298 K) δ [ppm] = 171.8, 160.1, 151.4, 148.7, 143.5, 128.5, 126.8, 125.7, 124.3, 122.3, 122.1, 120.1, 118.7, 115.4, 91.5, 62.8, 53.2, 42.3, 25.5, 22.2; ESI-TOF-HRMS: m/z calcd for $[M-4Na+3H]^-$ $C_{66}H_{61}N_2O_{17}$, 1349.4082; found 1349.4055 (error = - 2.0 ppm).

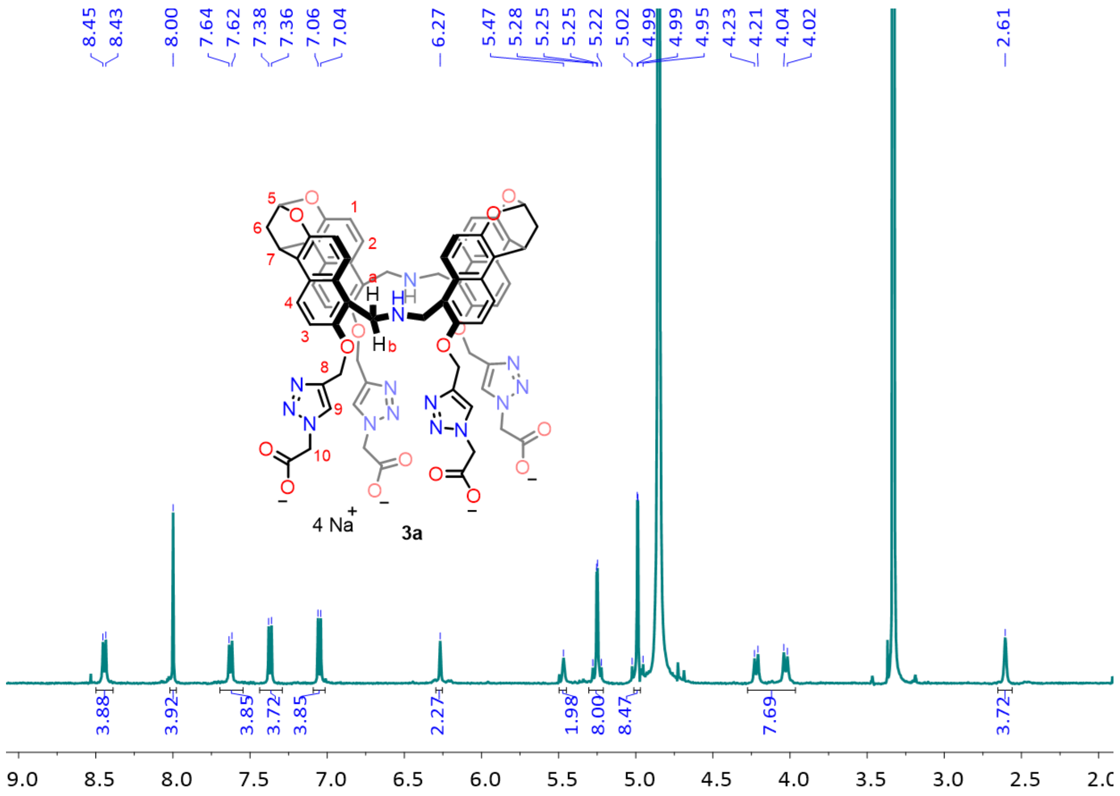

Supplementary Fig. 97 ^{1}H NMR spectrum (500 MHz, D_2O/MeOD = 1/4, 298 K) of compound **3a**.

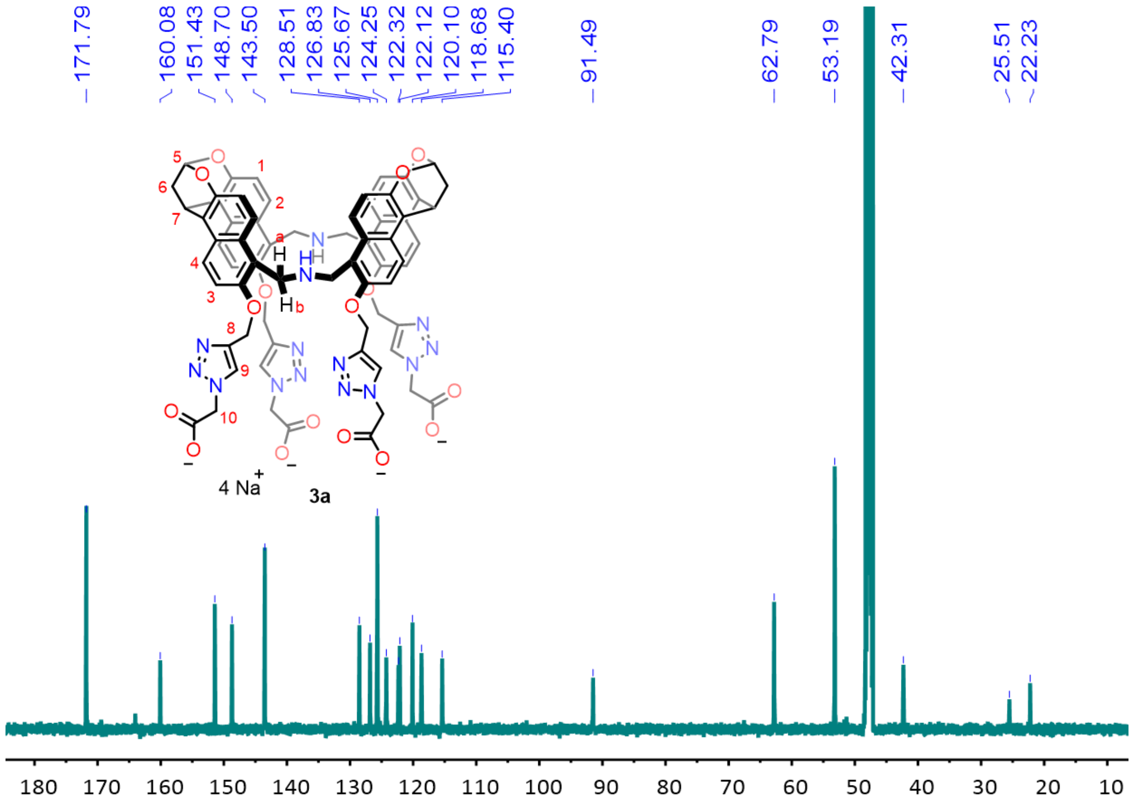

Supplementary Fig. 98 ^{13}C NMR spectrum (126 MHz, D_2O/MeOD = 1/4, 298 K) of compound **3a**.

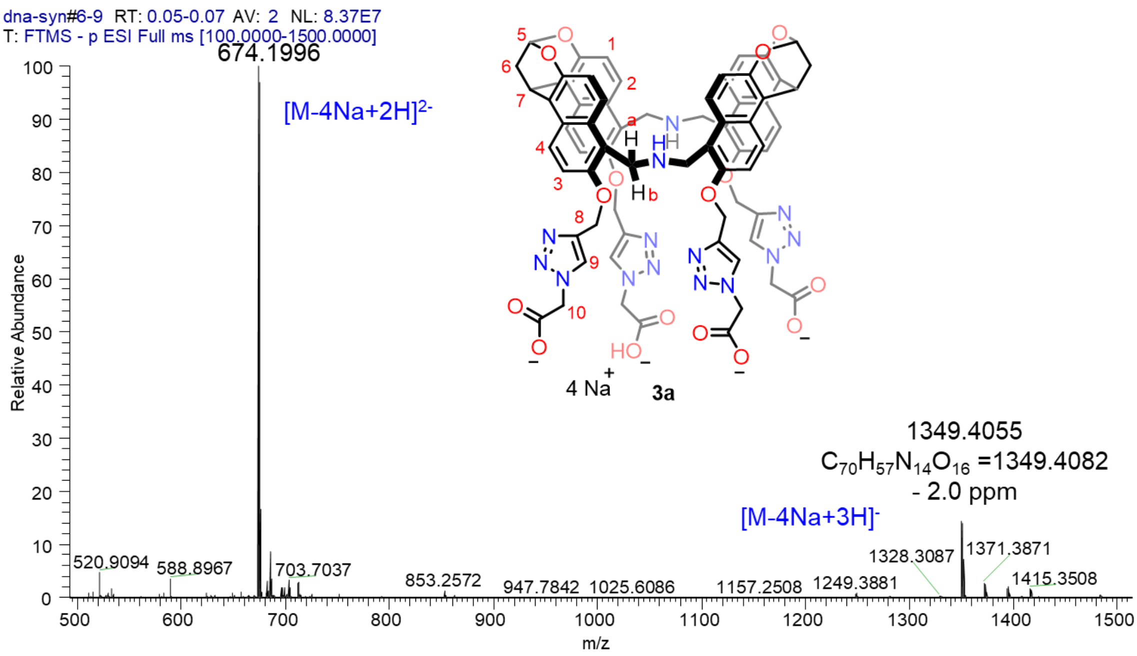

Supplementary Fig. 99 ESI mass spectrum of compound **3a**.

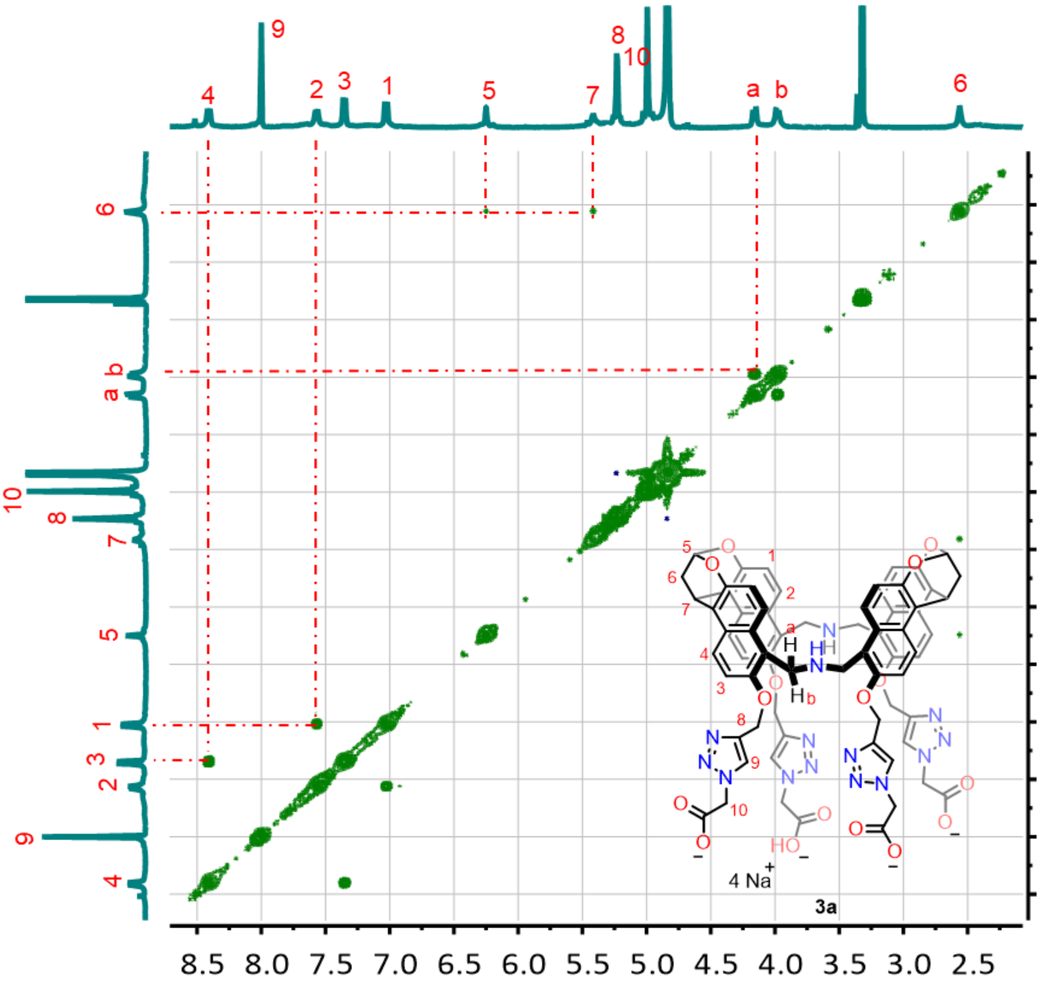

Supplementary Fig. 100 ^{1}H, ^{1}H-COSY NMR spectrum of **3a** (500 MHz, D_2O/MeOD = 1/4, 298 K).

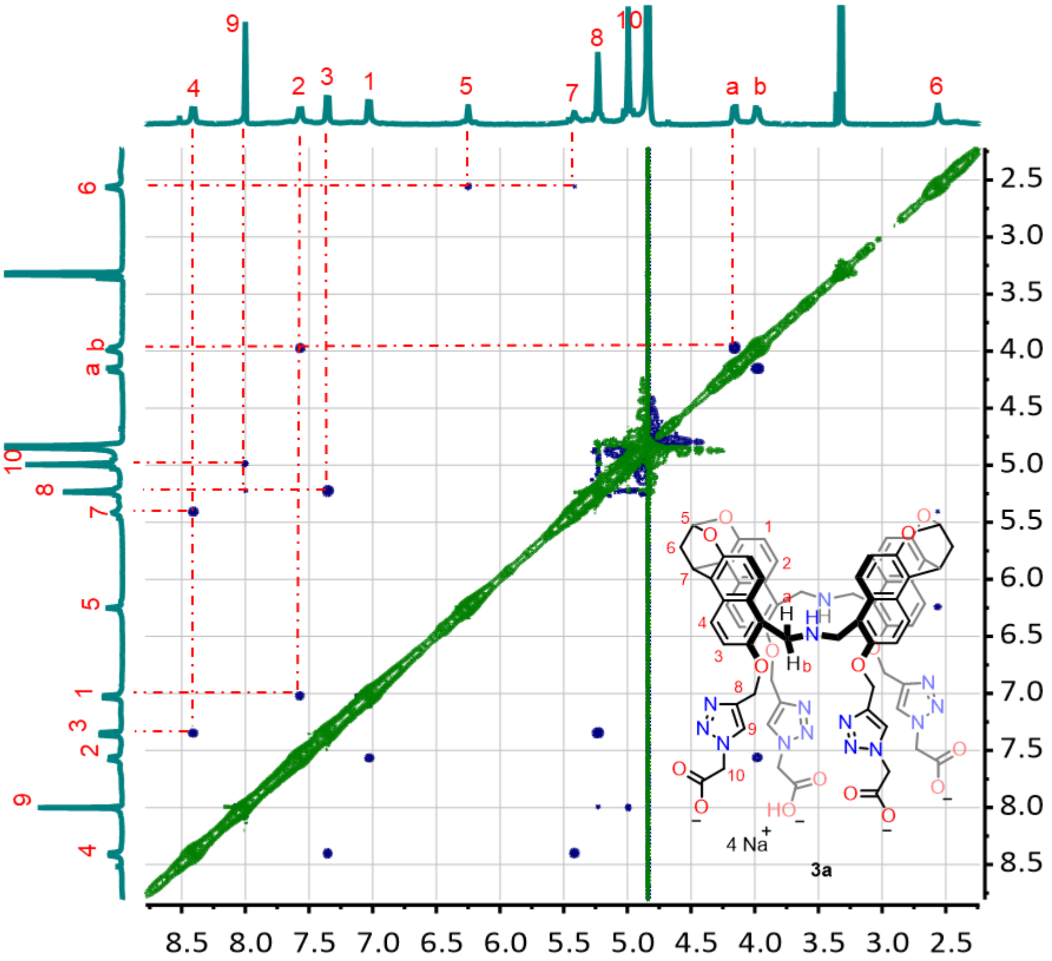

Supplementary Fig. 101 ^{1}H, ^{1}H-ROESY NMR spectrum of **3a** (500 MHz, D_2O/MeOD = 1/4, 298 K). No NOE effect was detected between the protons (9 and 10) and aromatic protons (1 and 2). This structure was assigned to the *syn* isomer, which was confirmed by the crystal structure of its precursor.[1]

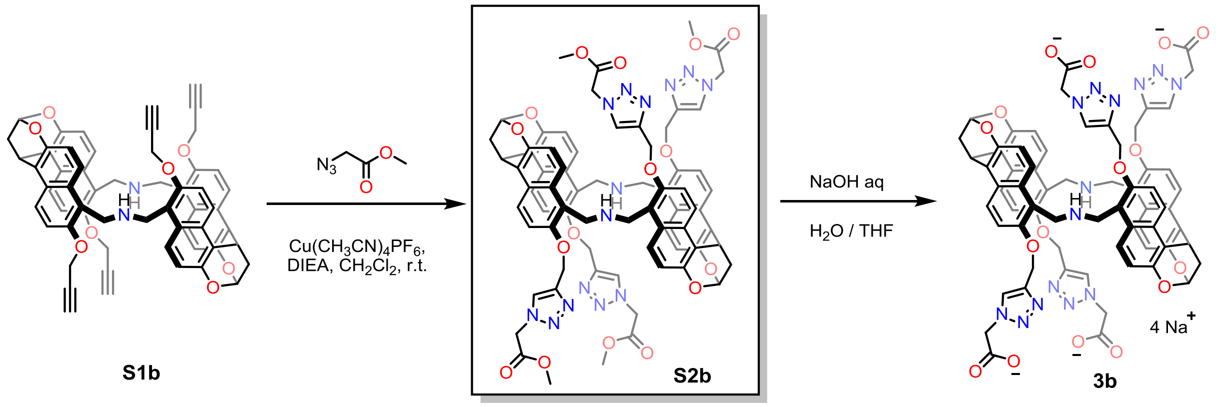

Supplementary Fig. 102 Synthetic procedures of **3b**

Compounds 3b. **S1b** (95 mg, 0.1 mmol), Methyl 2-azidoacetate (184 mg, 16 eq.), tetrakis(acetonitrile)copper(I) hexafluorophosphate (20 mg) and ethyldiisopropylamine (200

μL) were added to the solvent CH_2Cl_2 (300 mL) in a 500 mL two-necked flask at room temperature. Then the resulting mixture was stirred for another 24 h at 45 ^oC. All volatiles were removed under reduced pressure and the residue was poured into 20% sodium edetate water solution (50 mL), stirred for 30 min, and then filtered. The filter cake was washed by water to afford the crude product **S2b** which was used directly in the next step.

Crude product **S2b** was obtained from the above reaction, was loaded into a 250 mL one-neck flask charged with a magnetic stirring bar. MeOH (100 mL), H_2O (20 mL) and THF (100 mL) were added into the flask. Then the NaOH (80 mg, 2 mmol) was added. The mixture was stirred at room temperature. After 12 h, all volatiles were removed under reduced pressure. The residue was dissolved in H_2O (10 mL), and 3 N HCl were added dropwise to the solution to adjust the pH value to be 2-3. The mixture was filtered and washed by water extensively to get off-white solid (115 mg). The solid (115 mg) was loaded into a 250 mL one-neck flask, MeOH (10 mL) and H_2O (1 mL) was added into the flask. NaOH (48 mg, 1.2 mmol) was then added. The precipitate was then collected through filtration and dried to afford pure **3b** as a white solid (90 mg, yield 63% based on **S1b**).

3b, White solid, m.p. > 200 ^oC (decomposed); ^1H NMR (500 MHz, D_2O, 298 K) δ [ppm] = 8.40 (d, *J* = 9.6 Hz, 4H), 7.74 (s, 4H), 7.46 (d, *J* = 9.3 Hz, 4H), 7.20 (d, *J* = 9.3 Hz, 4H), 6.88 (d, *J* = 9.2 Hz, 4H), 6.25 (s, 2H), 5.40 (s, 2H), 4.87 (s, 8H), 4.68 – 4.49 (m, 8H), 4.19 (d, *J* = 12.9 Hz, 4H), 3.81 (d, *J* = 12.9 Hz, 4H), 2.57 (s, 4H); ^{13}C NMR (126 MHz, D_2O, 298 K) δ [ppm] = 172.9, 152.3, 148.6, 143.3, 128.1, 126.4, 125.6, 122.4, 120.3, 119.0, 115.4, 92.1, 62.7, 53.0, 41.6, 25.0, 22.3; ESI-TOF-HRMS: m/z calcd for $[M-4Na+3H]^-$ $C_{66}H_{61}N_2O_{17}$, 1349.4082; found 1349.4050 (error = - 2.4 ppm).

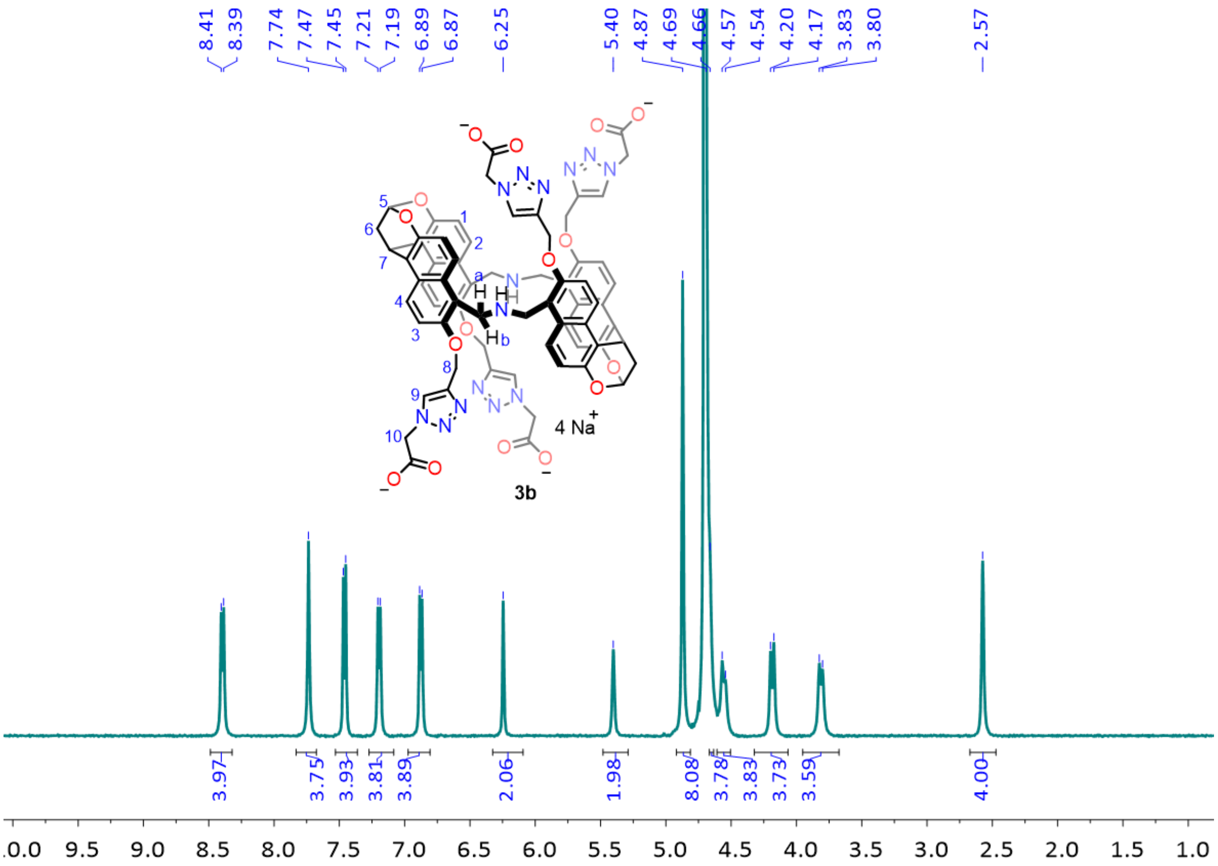

Supplementary Fig. 103 ^{1}H NMR spectrum (500 MHz, D_2O, 298 K) of compound **3b**.

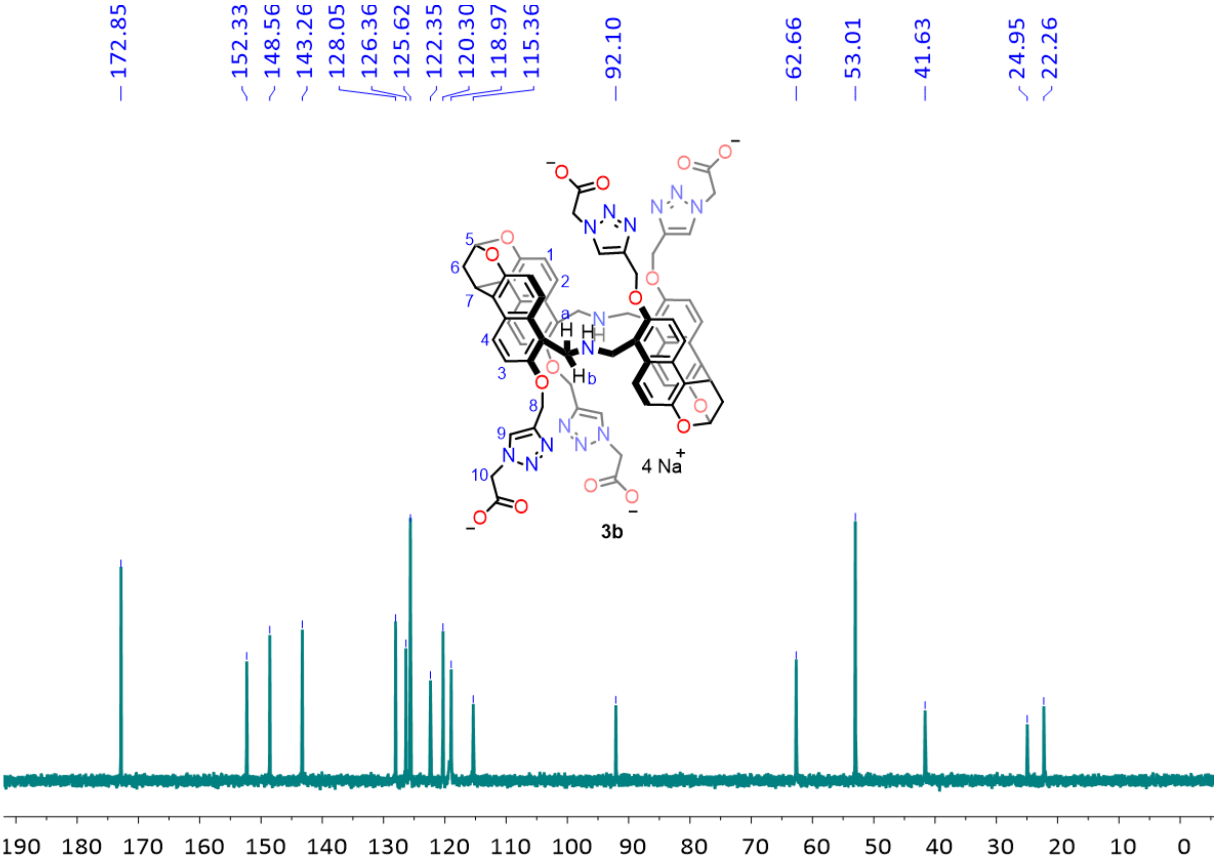

Supplementary Fig. 104 ^{13}C NMR spectrum (126 MHz, D_2O, 298 K) of compound **3b**.

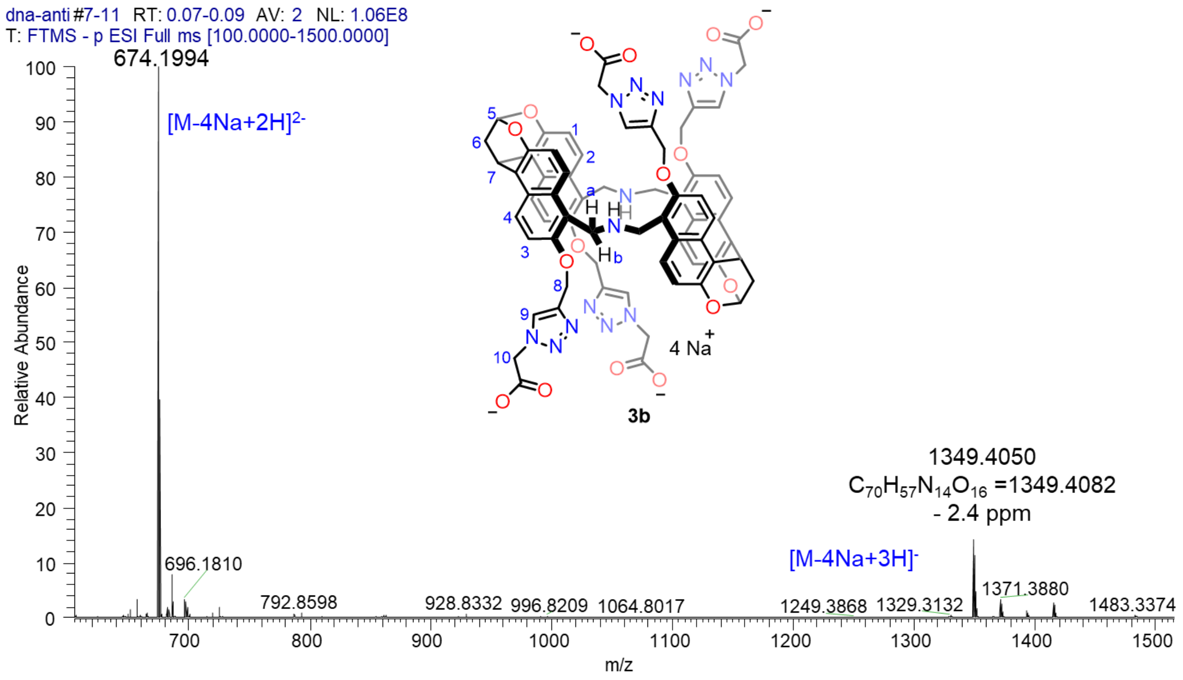

Supplementary Fig. 105 ESI mass spectrum of compound **3b**.

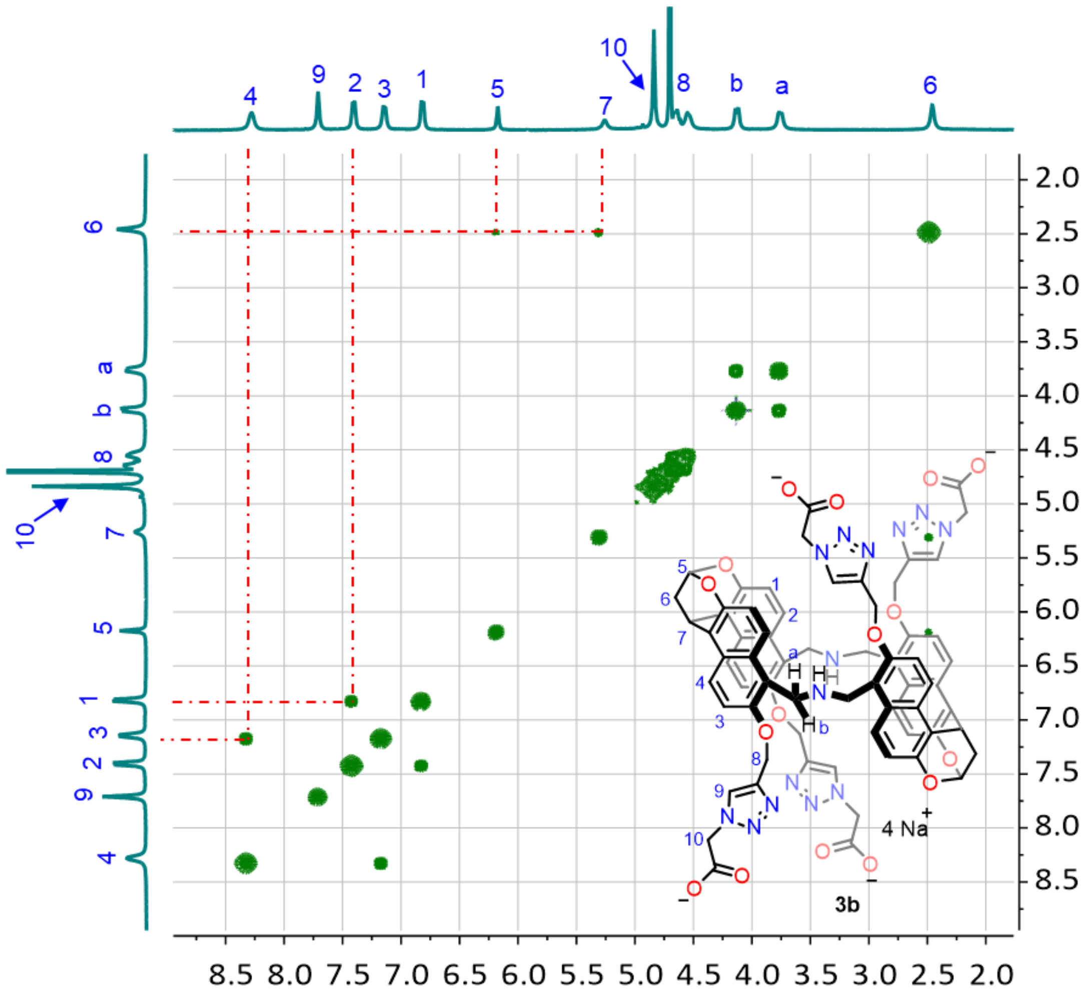

Supplementary Fig. 106 ^{1}H, ^{1}H-COSY NMR spectrum of **3b** (500 MHz, D_2O, 298 K).

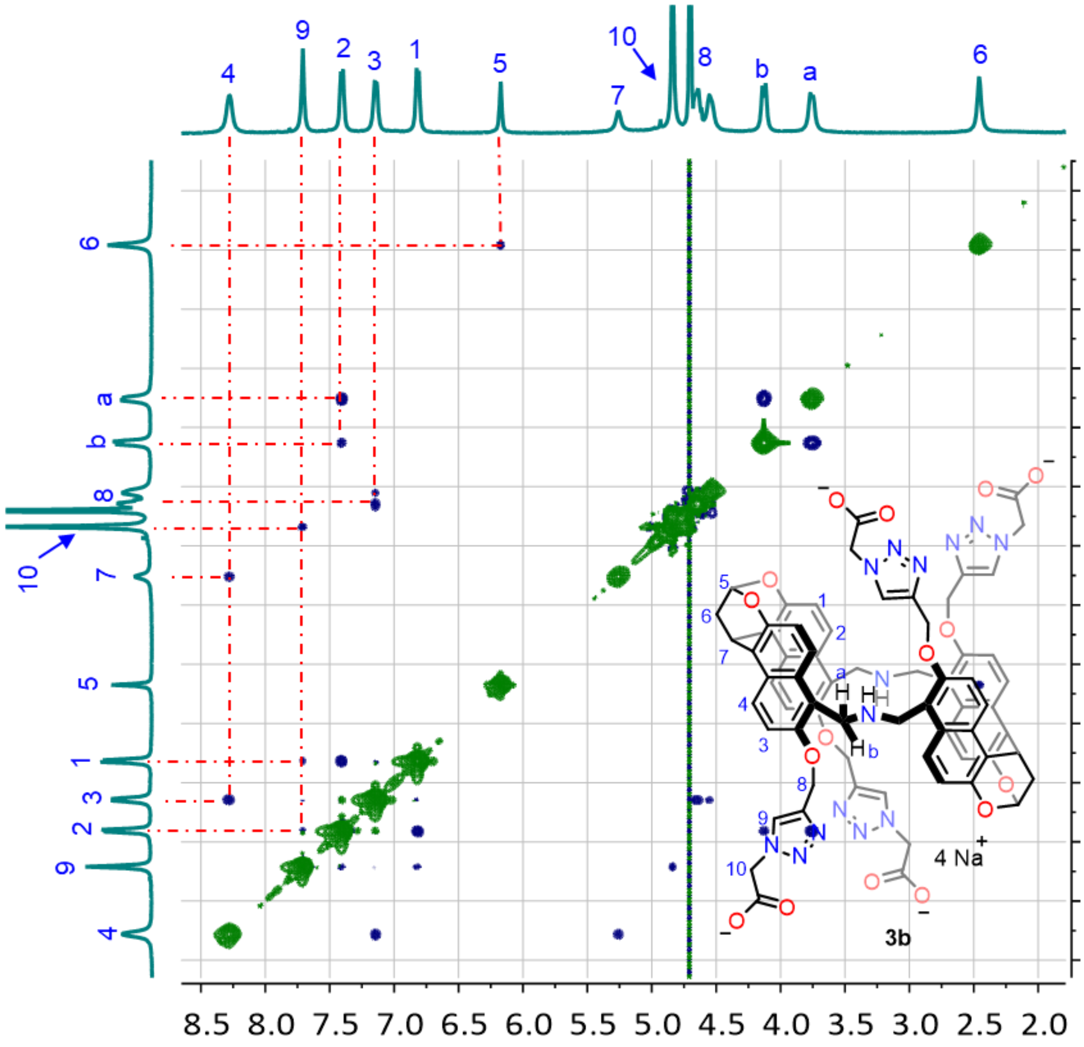

Supplementary Fig. 107 ^{1}H, ^{1}H-ROESY NMR spectrum of **3a** (500 MHz, D_2O, 298 K). NOE effect was detected between the proton 9 and aromatic protons (1 and 2). This structure was assigned to the *anti* isomer, which was confirmed by the crystal structure of its precursor.[1]

12.2 Synthesis of 4a and 4b

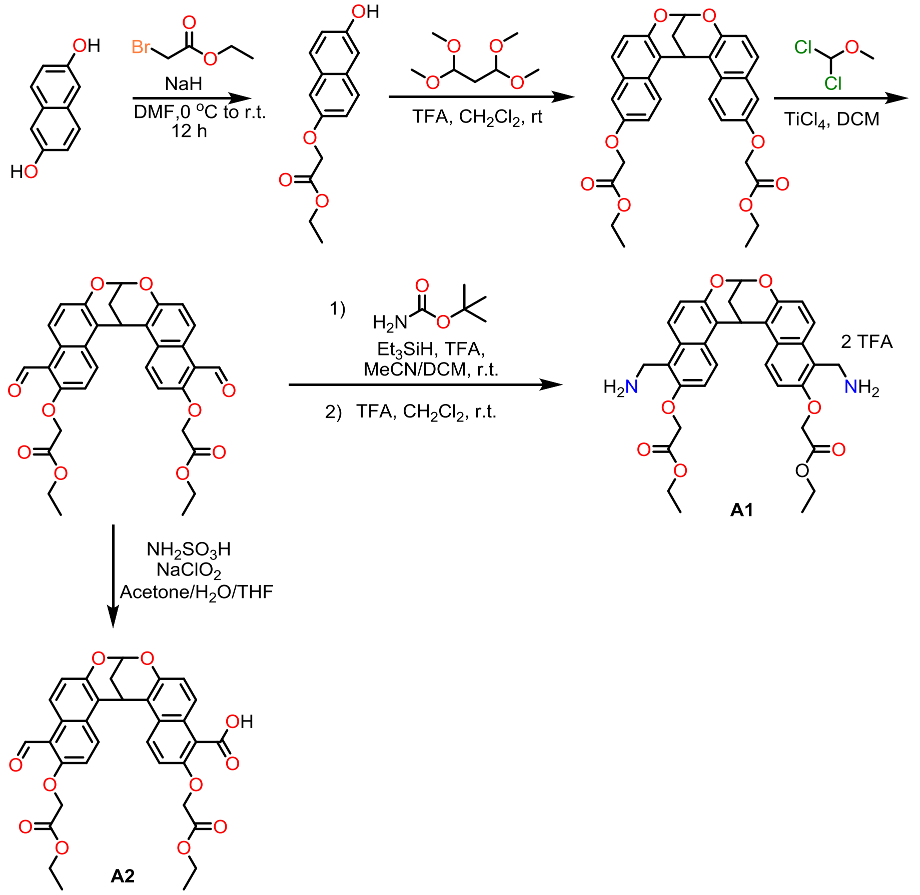

Supplementary Fig. 108 Synthetic procedures of **A1**[2] and **A2**[3].

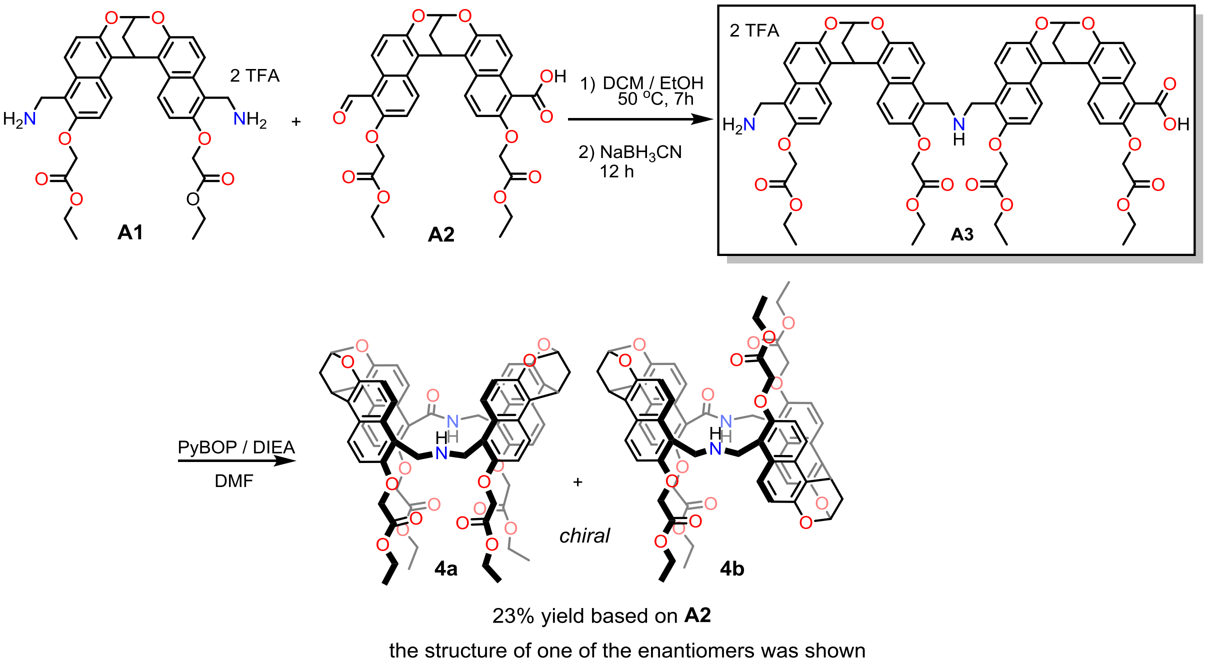

Supplementary Fig. 109 Synthetic procedures of **4a** and **4b**.

Compounds 4a and 4b. **A1** (4.07 g, 5.0 mmol) was added to the solvent mixture of ethanol (200 mL) and CH_2Cl_2 (300 mL) in a 1000 mL two-necked flask at room temperature. Then **A2** (3.0 g, 5.0 mmol) was added slowly via a syringe pump over 16 h at 45 ^oC. The mixture was stirred for another 6 h at 45 ^oC, and then $NaBH_3CN$ (0.95 g, 15.0 mmol) was added. Then the resulting mixture was stirred for another 12 h at 45 ^oC. All volatiles were removed under reduced pressure and the residue was poured into water (300 mL), stirred for 30 min, and then filtered. The filter cake was washed by water to afford the crude product **A3** which was used directly in the next step.

PyBOP (5.2 g, 10.0 mmol) and DIEA (10 mL) were added to DMF (500 mL) in a 1000 mL two-necked flask at room temperature. The solution of **A3** in DMF (40 mL), which was obtained from the above reaction, was added dropwise to the flask via a syringe pump over 16 h under Ar atmosphere. The resulting mixture was stirred for 24 h. Most of the solvent was removed under reduced pressure, and the residue was poured into water (4 L). The precipitate was collected through filtration, and suspended in MeOH. After sonicating for 30 min, the solid was collected through filtration and dried, which was further subjected to column chromatography (SiO_2, MeOH/CH_2Cl_2 = 2/500) to afford the mixture of **4a**·$H^+PF_6^-$ and **4b**·$H^+PF_6^-$ as a white solid (1.5 g, yield 23% based on **A2**).

The mixture of **4a**·$H^+PF_6^-$ and **4b**·$H^+PF_6^-$ (1.5 g) were added to the solvent mixture (THF/CH_2Cl_2 = 50 mL/50 mL). The resulting suspension was sonicated for 30 min, and the solid was collected through filtration and dried to afford pure **4a**·$H^+PF_6^-$ as a white solid (550 mg, yield 8% based on **A2**). The solvent in the filtrate was removed under reduced pressure, and the residue was added in the solvent mixture (Acetone/CH_2Cl_2 = 50 mL/50 mL). The resulting mixture was sonicated for 1 h. The precipitate was then collected through filtration and dried to afford pure **4b**·$H^+PF_6^-$ as a white solid (760 mg, yield 11% based on **A2**).

4a·H^+ PF_6^-, White solid, m.p. > 200 ^oC (decomposed); ^1H NMR (500 MHz, acetone-d_6, 298 K) δ [ppm] = 8.68 (d, *J* = 9.4 Hz, 1H), 8.63 (d, *J* = 9.4 Hz, 1H), 8.57 (d, *J* = 9.4 Hz, 1H), 8.54 (d, *J* = 9.4 Hz, 1H), 8.12 (d, *J* = 9.2 Hz, 1H), 8.09 (d, *J* = 9.2 Hz, 1H), 7.93 (d, *J* = 9.2 Hz, 1H), 7.92 (d, *J* = 9.2 Hz, 1H), 7.81 (s, 1H), 7.65 (s, 1H), 7.35 – 7.27 (m, 3H), 7.20 (d, *J* = 9.2 Hz, 1H), 7.19 (d, *J* = 9.2 Hz, 1H), 7.15 (d, *J* = 9.4 Hz, 1H), 7.09 (d, *J* = 9.2 Hz, 2H), 6.62 (d, *J* = 9.8 Hz, 1H), 6.28 (s, 1H), 6.27 (s, 1H), 5.54 – 5.48 (m, 2H), 5.41 (dd, *J* = 13.9, 10.0 Hz, 1H), 5.30 – 5.13 (m, 4H), 5.04 (d, *J* = 16.9 Hz, 1H), 5.01 – 4.89 (m, 2H), 4.89 – 4.71 (m, 6H), 4.30 – 3.89 (m, 8H), 2.67 – 2.43 (m, 4H), 1.28 – 1.20 (m, 6H), 1.17 (t, *J* = 7.1 Hz, 3H), 1.06 (t, *J* = 7.1 Hz, 3H); ^{13}C NMR (126 MHz, acetone-d_6, 298 K) δ [ppm] = 170.5, 170.4, 169.9, 165.7, 153.7, 153.5, 151.3, 150.9, 150.5, 150.1, 149.9, 129.6, 129.4, 129.4, 128.3, 128.2, 128.1,

128.0, 127.9, 127.3, 127.2, 125.9, 125.8, 124.6, 123.6, 123.5, 122.7, 122.5, 121.3, 121.0, 120.9, 120.6, 120.5, 120.3, 120.2, 115.8, 115.7, 115.5, 114.4, 114.3, 114.0, 92.3, 92.2, 67.6, 66.9, 66.8, 66.4, 62.3, 62.2, 62.1, 61.8, 44.2, 44.1, 44.0, 33.7, 26.5, 26.4, 23.1, 23.0, 14.61, 14.59, 14.5, 14.4; ^{19}F NMR (376 MHz, acetone-d_6) δ [ppm] = -72.54 (d, J = 707.8 Hz); ^{31}P NMR (162 MHz, acetone-d_6) δ [ppm] = -142.08 (hept, J = 707.8 Hz); ESI-TOF-HRMS: m/z calcd for [M-PF$_6$]$^+$ $C_{66}H_{61}N_2O_{17}$, 1153.3965; found 1153.3981 (error = + 1.4 ppm).

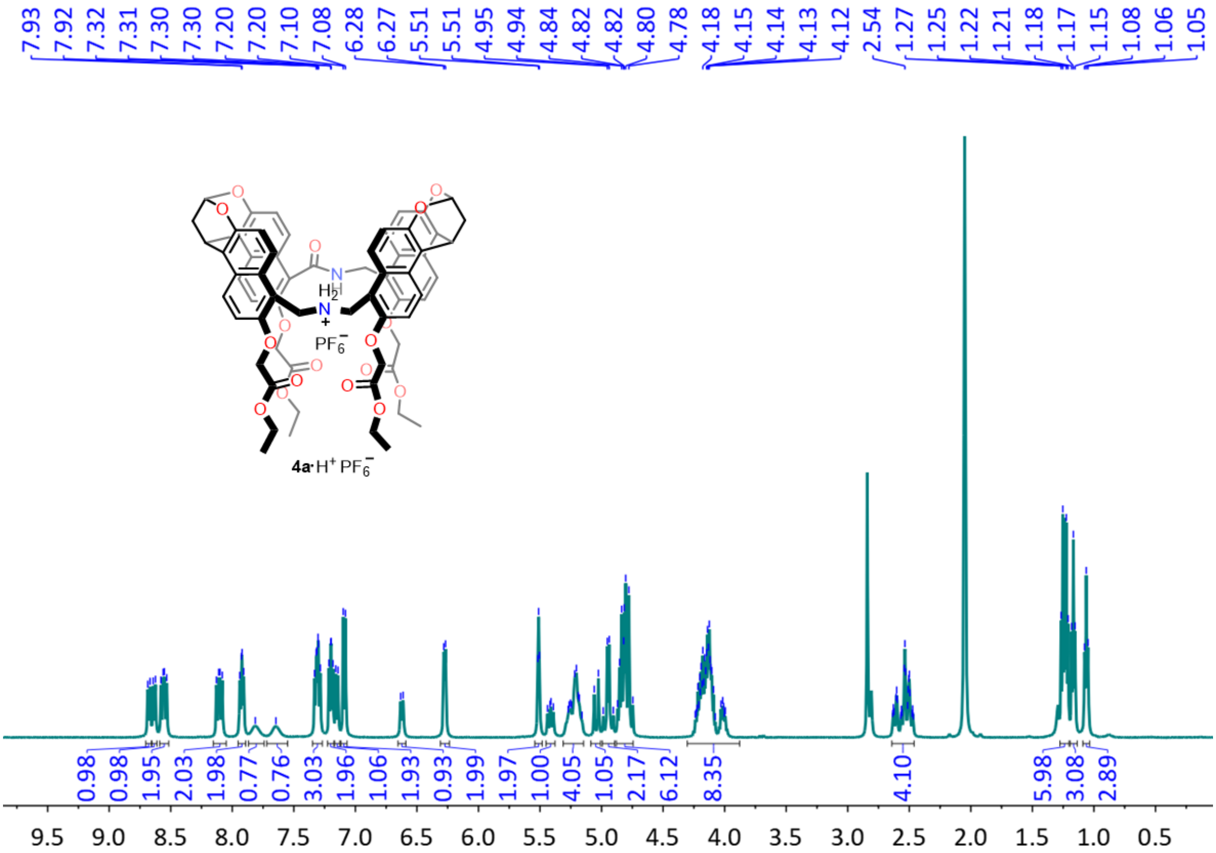

Supplementary Fig. 110 ^{1}H NMR spectrum (500 MHz, acetone-d_6, 298 K) of compound **4a**·H$^+$ PF$_6^-$.

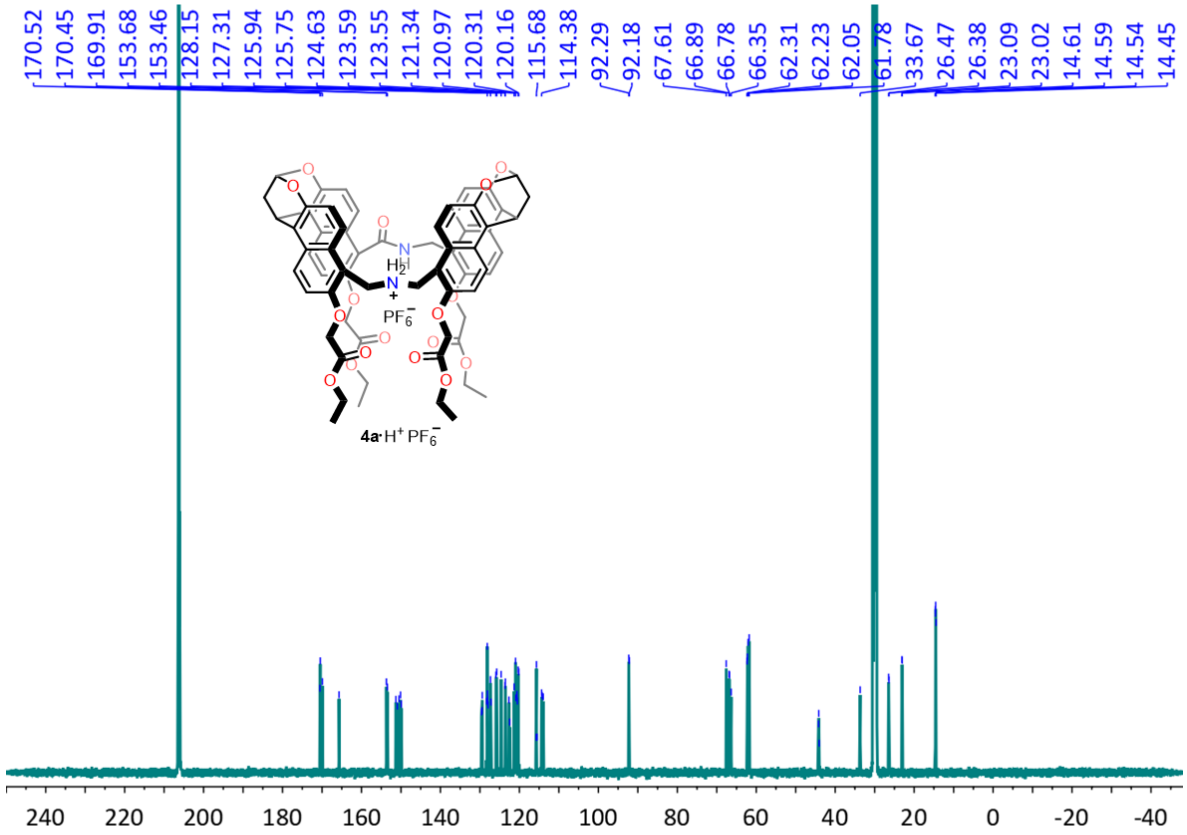

Supplementary Fig. 111 ^{13}C NMR spectrum (126 MHz, acetone-d_6, 298 K) of compound **4a**·H$^+$ PF_6^-.

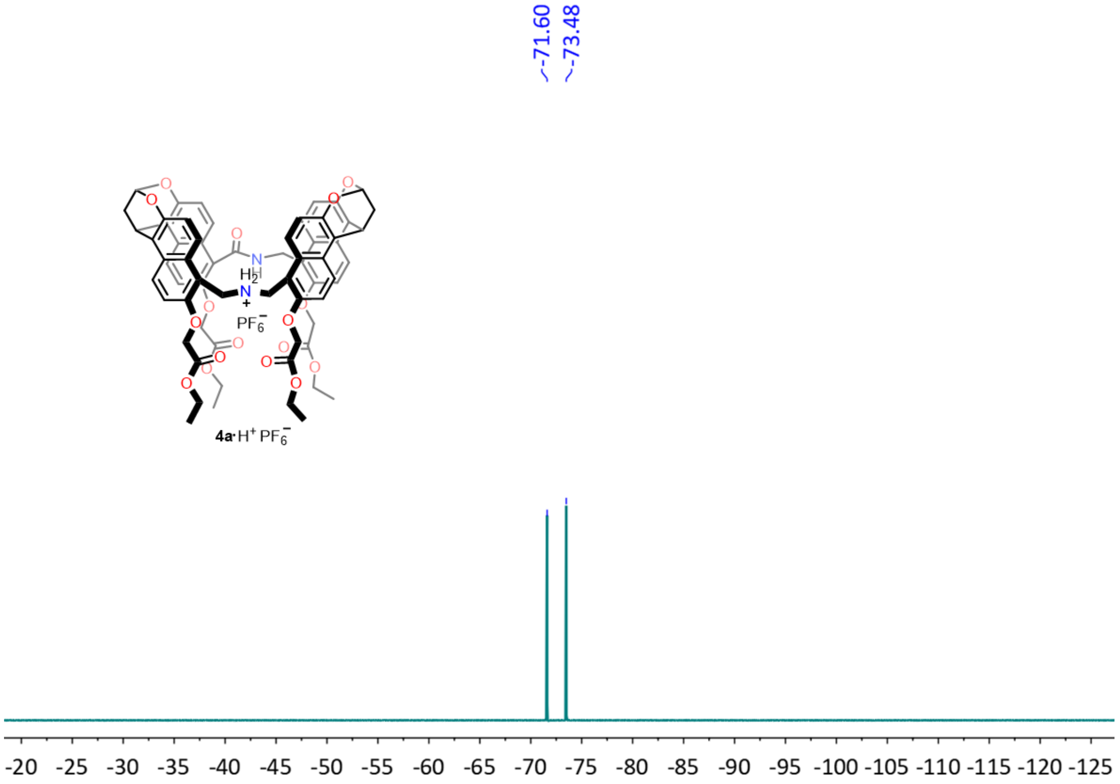

Supplementary Fig. 112 ^{19}F NMR spectrum (376 MHz, acetone-d_6, 298 K) of compound **4a**·H$^+$ PF_6^-.

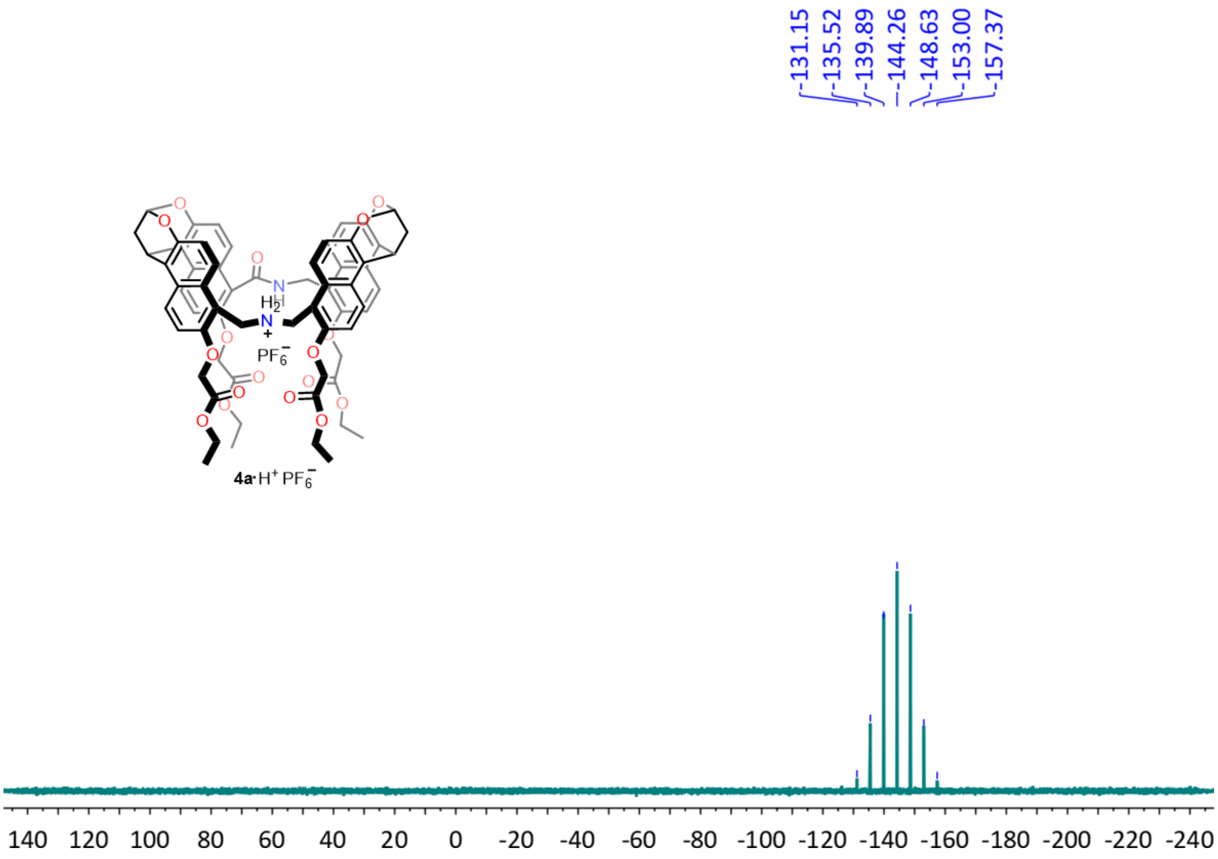

Supplementary Fig. 113 ^{31}P NMR spectrum (162 MHz, acetone-d_6, 298 K) of compound **4a**·H$^+$ PF_6^-.

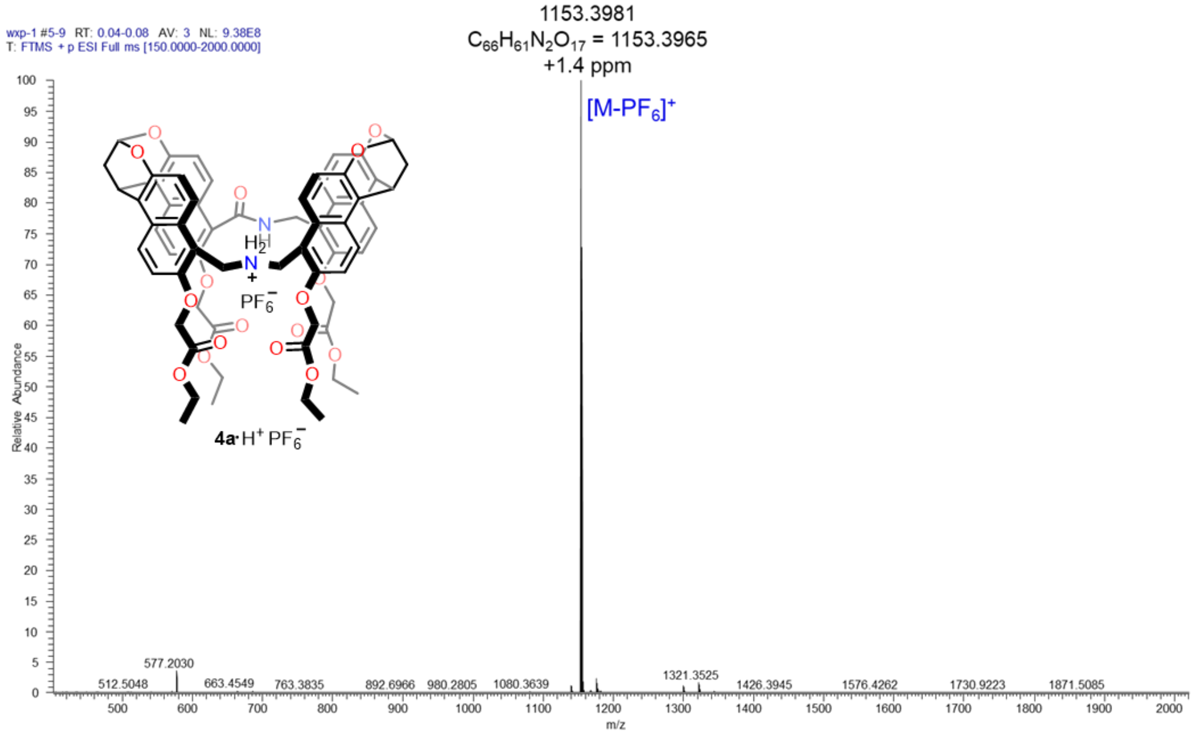

Supplementary Fig. 114 ESI mass spectrum of compound **4a**·H$^+$ PF_6^-.

4b·H$^+$ PF_6^-, White solid, m.p. > 250 ^oC (decomposed); ^1H NMR (500 MHz, CD_2Cl_2, 298 K) δ [ppm] = 8.45 (d, *J* = 9.5 Hz, 1H), 8.39 (d, *J* = 9.5 Hz, 1H), 8.33 (d, *J* = 9.1 Hz, 1H), 8.31 (d, *J* = 9.1 Hz, 1H), 7.72 (d, *J* = 9.2 Hz, 1H), 7.69 (d, *J* = 9.2 Hz, 1H), 7.65 (d, *J* = 9.2 Hz, 1H), 7.57 (d, *J* = 9.1 Hz, 1H), 7.20 (s, 2H), 7.18 – 7.07 (m, 4H), 7.06 – 6.99 (m, 3H), 6.94 (d, *J* = 9.1 Hz, 1H), 6.24 (s, 1H), 6.22 (s, 1H), 5.57 (dd, *J* = 13.8, 7.0 Hz, 1H), 5.45 (d, *J* = 6.3 Hz, 1H), 5.30 (s, 1H), 5.27 (s, 1H), 5.09 – 4.99 (s, 1H), 4.91 – 4.64 (m, 10H), 4.52 (dd, *J* = 13.7, 2.0 Hz, 1H), 4.31 – 4.18 (m, 2H), 4.14 – 4.04 (m, 1H), 4.03 – 3.91 (m, 1H), 3.51 – 3.31 (m, 2H), 3.10 – 3.02 (m, 1H), 3.01 – 2.93 (m, 1H), 2.68 – 2.60 (m, 2H), 2.54 – 2.46 (m, 2H), 1.29 (t, *J* = 7.1 Hz, 3H), 1.09 (t, *J* = 7.1 Hz, 3H), 0.95 (t, *J* = 7.1 Hz, 3H), 0.90 (t, *J* = 7.1 Hz, 3H); ^{13}C NMR (126 MHz, DMSO-d_6, 298 K) δ [ppm] = 170.1, 169.9, 169.7, 169.4, 165.5, 153.2, 153.2, 151.6, 149.9, 149.6, 149.2, 149.1, 149.0, 128.6, 128.5, 128.2, 127.4, 127.21, 127.18, 127.1, 127.0, 126.8, 126.7, 126.5, 125.7, 124.4, 123.5, 123.4, 123.3, 122.1, 120.4, 120.4, 120.4, 120.3, 119.8, 119.7, 119.5, 119.3, 115.0, 114.8, 114.6, 114.5, 114.4, 91.2, 91.1, 67.0, 66.8, 66.2, 65.8, 61.4, 61.28, 61.26, 61.2, 43.2, 42.6, 33.6, 25.6, 25.5, 21.7, 14.5, 14.47, 14.45; ^{19}F NMR (376 MHz, DMSO-d_6) δ [ppm] = -70.58 (d, *J* = 711.2 Hz); ^{31}P NMR (162 MHz, DMSO-d_6) δ [ppm] = -144.65 (hept, *J* = 711.2 Hz); ESI-TOF-HRMS: m/z calcd for $[M-PF_6]^+$ $C_{66}H_{61}N_2O_{17}$, 1153.3965; found 1153.3971 (error = + 0.5 ppm).

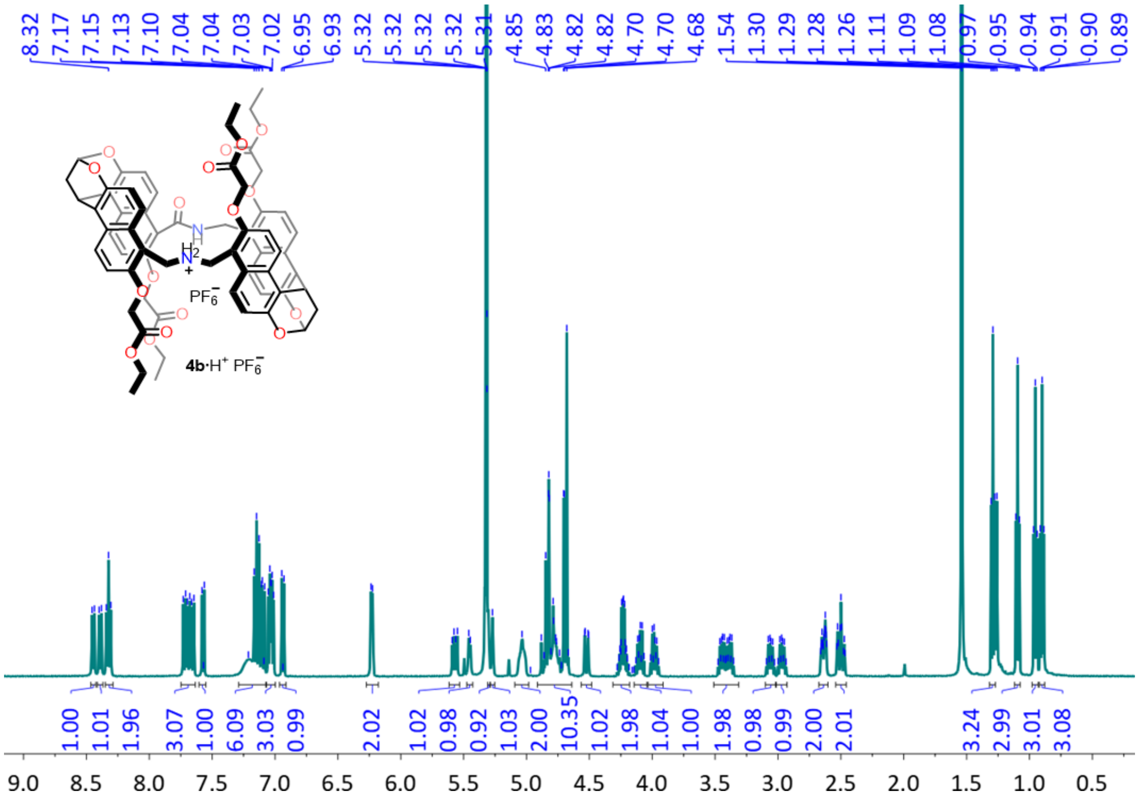

Supplementary Fig. 115 ^1H NMR spectrum (500 MHz, CD_2Cl_2, 298 K) of compound **4b**·H$^+$ PF_6^-.

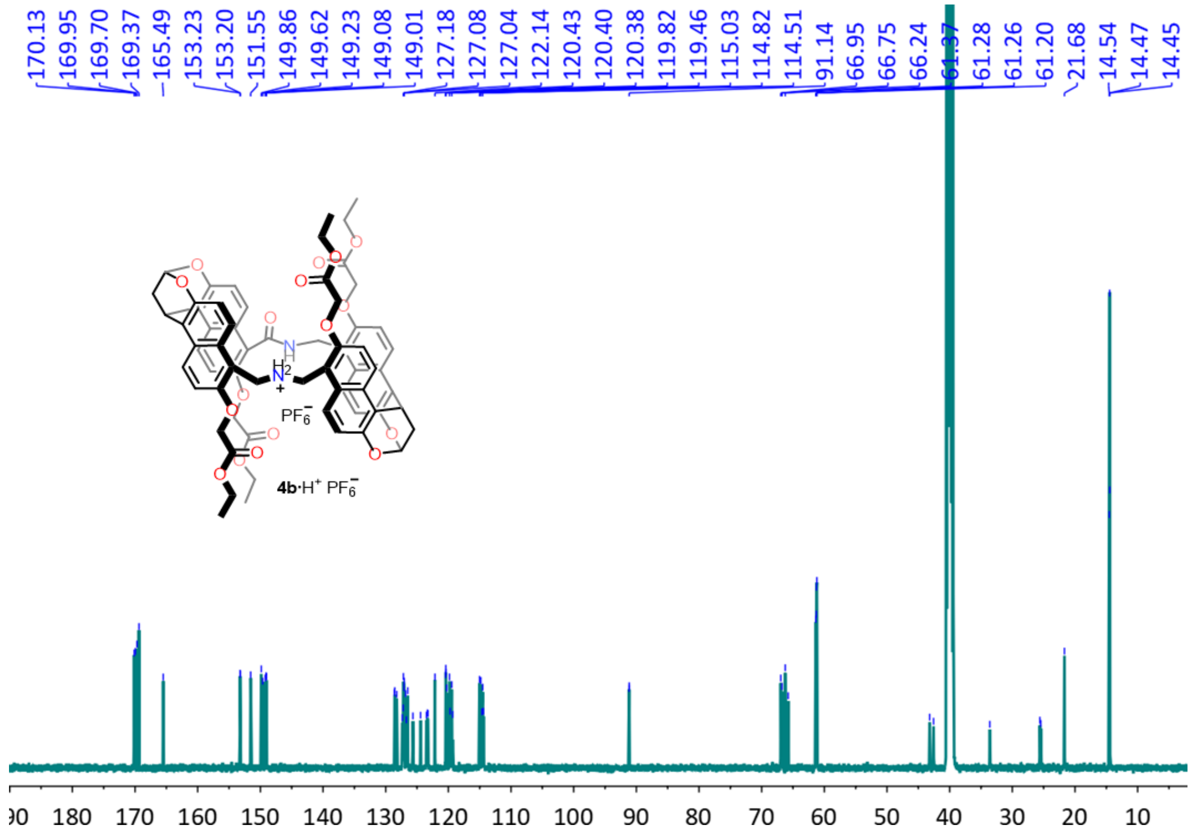

Supplementary Fig. 116 ^{13}C NMR spectrum (126 MHz, DMSO-d_6, 298 K) of compound **4b**·H$^+$ PF_6^-.

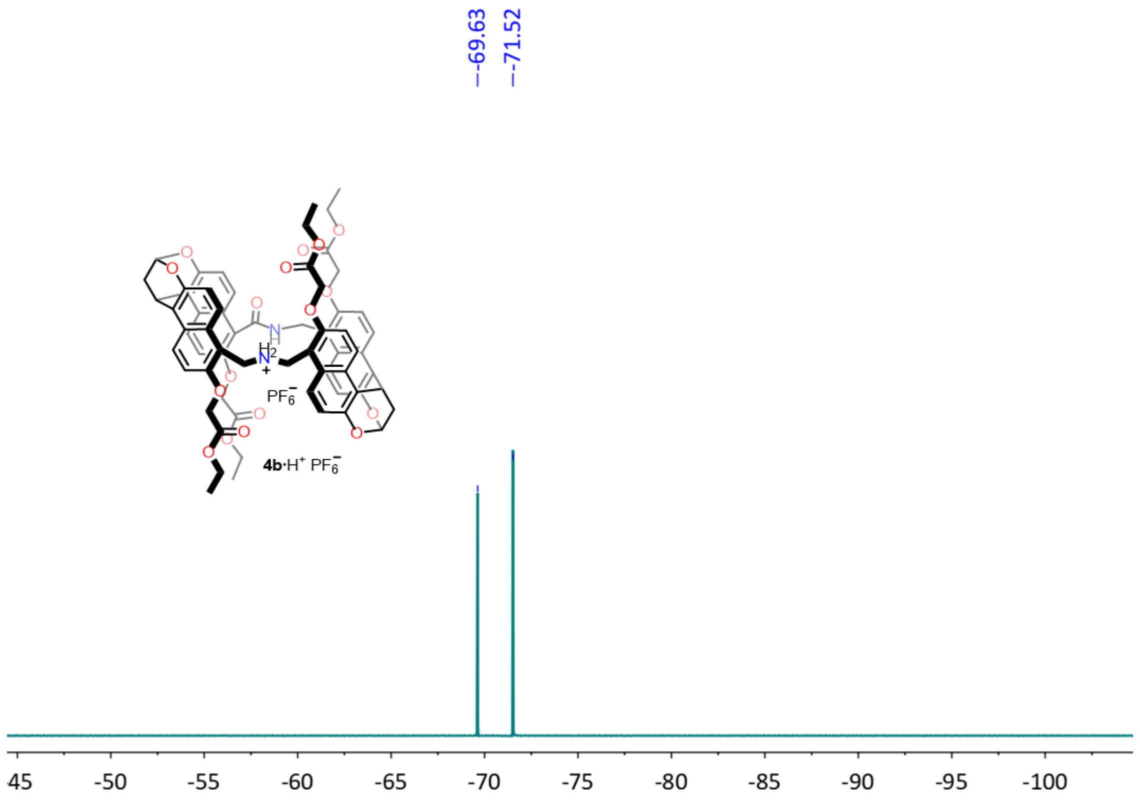

Supplementary Fig. 117 ^{19}F NMR spectrum (376 MHz, DMSO-d_6, 298 K) of compound **4b**·H$^+$ PF_6^-.

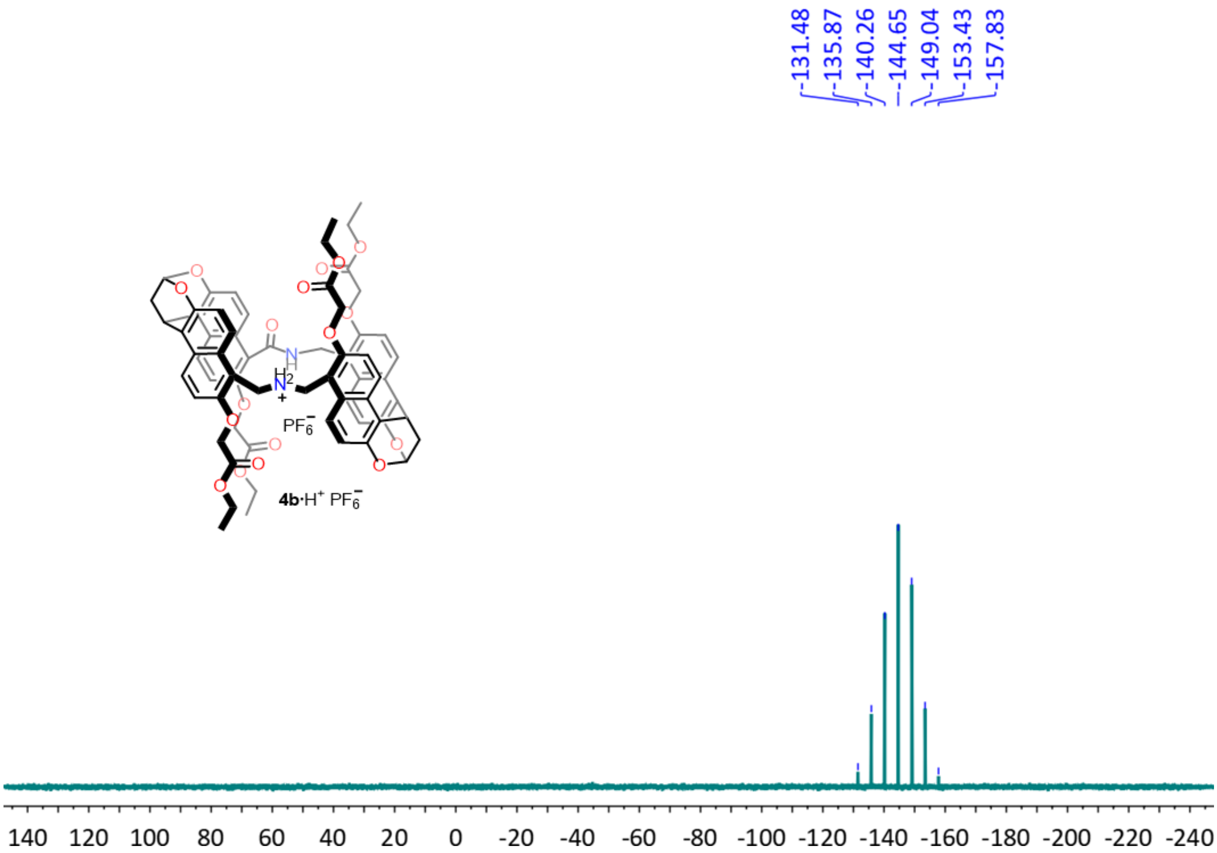

Supplementary Fig. 118 ^{31}P NMR spectrum (162 MHz, DMSO-d_6, 298 K) of compound **4b**·H$^+$ PF_6^-.

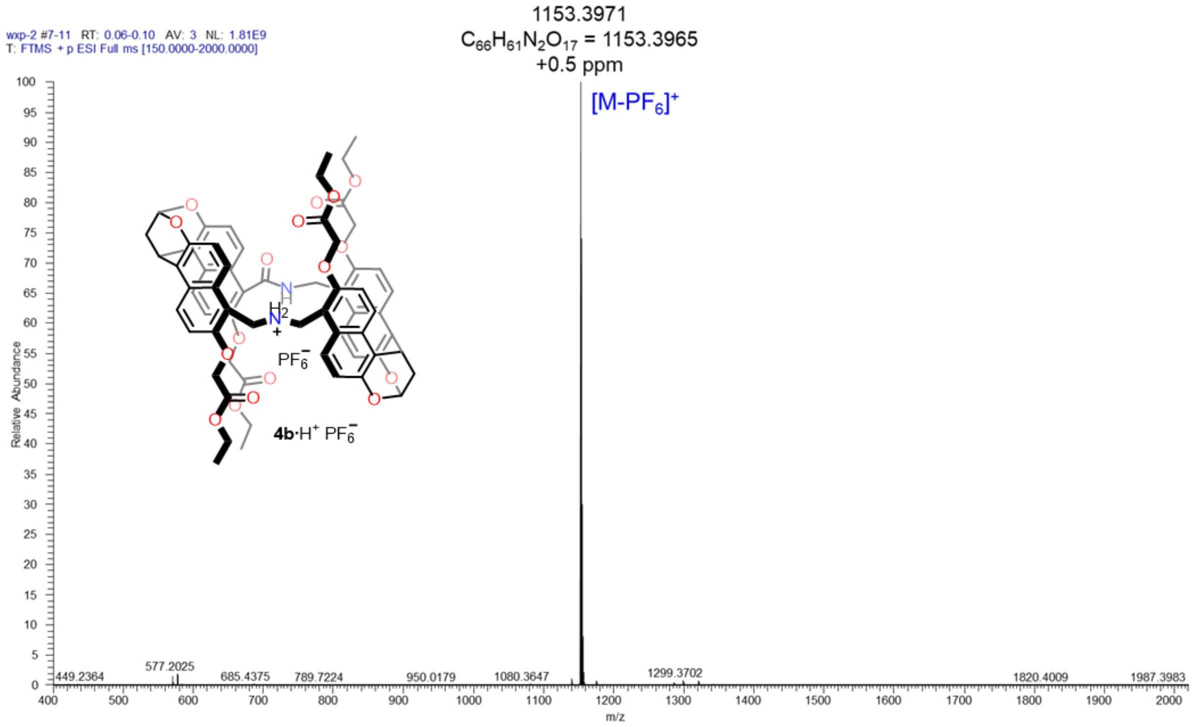

Supplementary Fig. 119 ESI mass spectrum of compound **4b**·H$^+$ PF_6^-.

12.3 Synthesis of 5a and 5b

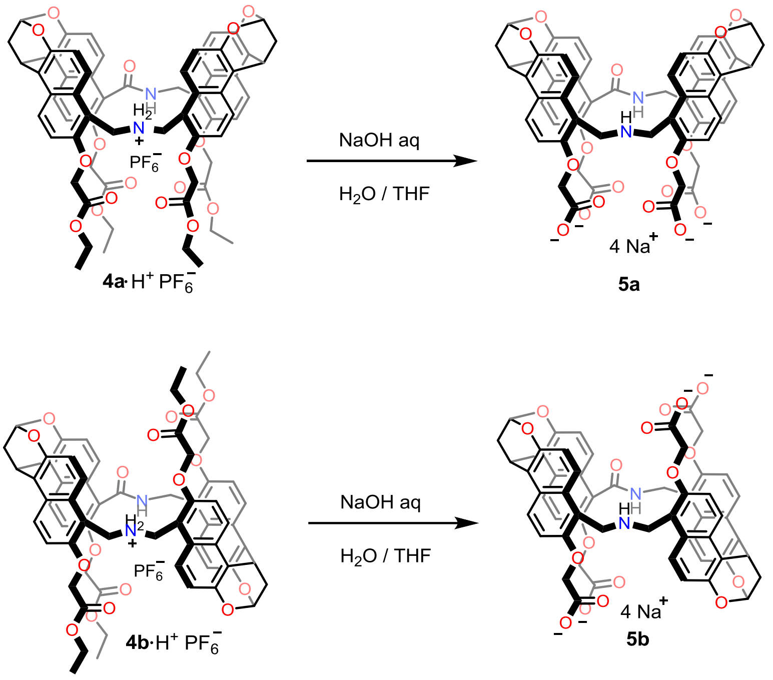

Supplementary Fig. 120 Synthetic procedures of **5a** and **5b**

Synthesis of 5a and 5b. **4a**·H^+ PF_6^-/**4b**·H^+ PF_6^- (200 mg, 0.152 mmol) was loaded into a 250-mL one-neck flask charged with a magnetic stirring bar. MeOH (100 mL), H_2O (20 mL) and THF (100 mL) were added into the flask. Then the NaOH (48.8 mg, 1.22 mmol) was added. The mixture was stirred at room temperature. After 12 h, all volatiles were removed under reduced pressure. The residue was dissolved in H_2O (10 mL), and 3 N HCl were added dropwise to the solution to adjust the pH value to be 2-3. The mixture was filtered and washed by water extensively to get off-white solid (158 mg, quantitative). The solid (158 mg, 0.152 mmol) was loaded into a 250-mL one-neck flask, and H_2O (20 mL) was added into the flask. NaOH (24.4 mg, 0.61 mmol) was then added. The mixture was colorless and transparent. Then all the volatiles were removed under reduced pressure and the residue was the desired product **5a/5b** (171 mg, 0.152 mmol, yield > 99% based on **4a/4b**).

5a, m.p. > 250 ^oC (decomposed); ^1H NMR (500 MHz, D_2O, 298 K) δ [ppm] = 8.47 (d, J = 9.5 Hz, 1H), 8.44 (d, J = 9.6 Hz, 1H), 8.41 (d, J = 9.6 Hz, 1H), 8.33 (d, J = 9.5 Hz, 1H), 7.90 (d, J = 9.2 Hz, 1H), 7.69 (d, J = 9.2 Hz, 1H), 7.23 (d, J = 9.1 Hz, 1H), 7.19 (d, J = 9.1 Hz, 1H), 7.14 (d, J = 9.3 Hz, 1H), 7.08 – 7.01 (m, 3H), 6.99 – 6.89 (m, 3H), 6.78 (d, J = 9.1 Hz, 1H), 6.27 (s, 1H), 6.23 (s, 1H), 5.42 (s, 1H), 5.39 (s, 1H), 5.05 (d, J = 14.2 Hz, 1H), 4.84 – 4.81 (m, 2H), 4.48 – 4.35 (m, 9H), 4.26 – 4.07 (m, 2H), 2.60 (d, J = 13.4 Hz, 2H), 2.47 (d, J = 13.5 Hz, 2H); ^{13}C NMR (126 MHz, D_2O, 298 K) δ [ppm] = 177.1, 177.0, 176.8, 169.1, 152.6, 152.4, 152.1, 150.2, 148.9, 148.7, 147.9, 128.4, 128.0, 126.4, 126.1, 125.3, 125.0, 124.9, 123.3,

123.3, 123.2, 122.3, 121.1, 120.4, 120.2, 120.1, 119.9, 119.8, 119.6, 119.3, 119.0, 118.8, 118.0, 114.2, 113.8, 113.8, 113.6, 91.9, 91.8, 67.9, 67.8, 67.6, 67.5, 41.5, 41.2, 34.1, 25.1, 22.1, 22.0; ESI-TOF-HRMS: m/z calcd for $[M-4Na+3H]^-$ $C_{58}H_{43}N_2O_{17}$, 1039.2567; found 1039.2547 (error = - 1.9 ppm).

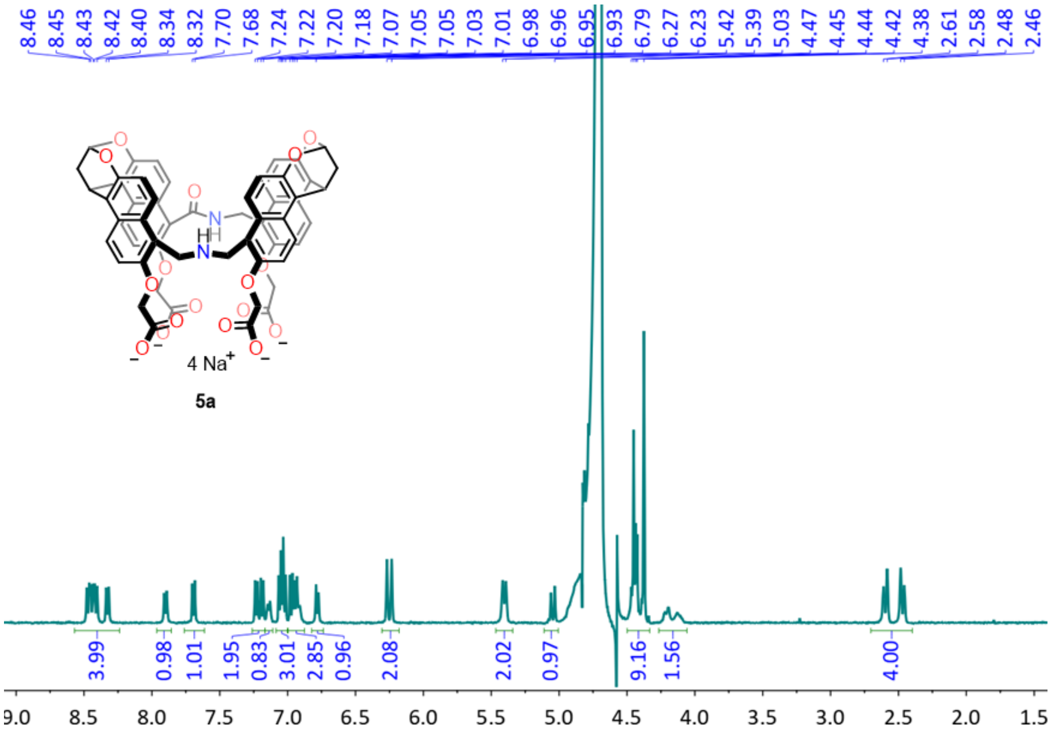

Supplementary Fig. 121 ^{1}H NMR spectrum (500 MHz, D_2O, 298 K) of compound **5a**

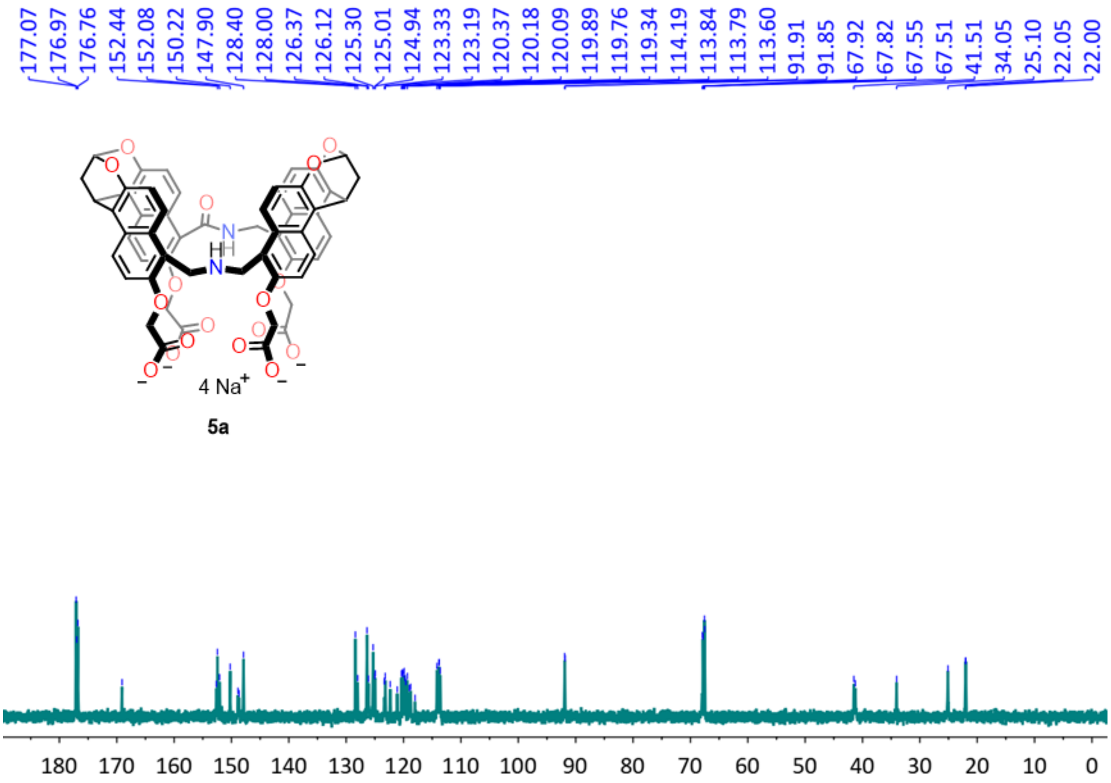

Supplementary Fig. 122 ^{13}C NMR spectrum (126 MHz, D_2O, 298 K) of compound **5a**

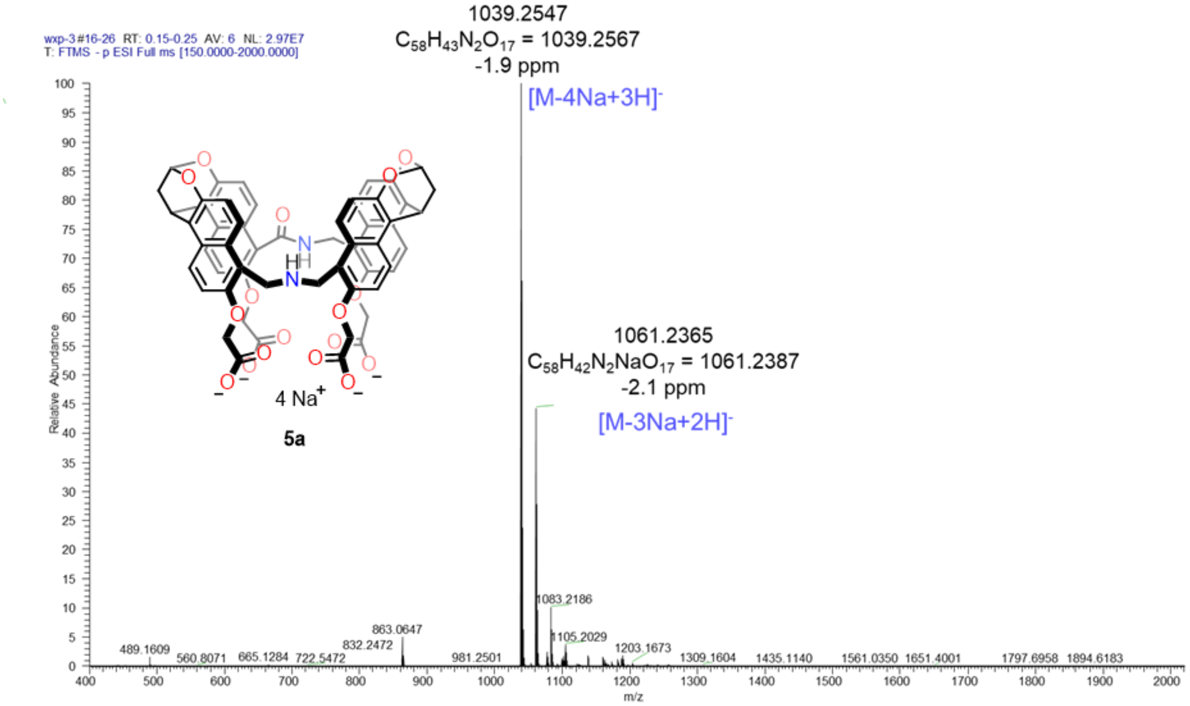

Supplementary Fig. 123 ESI mass spectrum of compound **5a**

5b, m.p. > 250 ^{o}C (decomposed); ^{1}H NMR (500 MHz, D_2O, 298 K) δ [ppm] = 8.47 (d, *J* = 9.5 Hz, 2H), 8.42 (d, *J* = 9.5 Hz, 2H), 7.68 (d, *J* = 9.3 Hz, 1H), 7.64 (d, *J* = 9.3 Hz, 1H), 7.59 (d, *J* = 9.3 Hz, 1H), 7.24 (d, *J* = 9.3 Hz, 1H), 7.13 – 6.95 (m, 8H), 6.24 (s, 2H), 5.44 (s, 1H), 5.44 (s, 1H), 5.43 (s, 1H), 5.10 (d, *J* = 14.0 Hz, 1H), 4.68 – 4.58 (m, 4H), 4.56 – 4.20 (m, 9H), 2.63 – 2.51 (m, 4H); ^{13}C NMR (126 MHz, D_2O, 298 K) δ [ppm] = 176.9, 176.61, 176.59, 170.0, 152.4, 152.2, 150.4, 148.5, 148.4, 147.9, 128.6, 128.24, 128.22, 126.7, 126.4, 125.7, 125.6, 125.4, 125.1, 123.3, 122.8, 122.5, 122.3, 120.0, 119.9, 119.8, 119.7, 119.6, 119.4, 119.3, 119.0, 117.0, 114.2, 113.8, 113.6, 113.3, 92.0, 67.9, 67.4, 67.2, 42.1, 34.8, 24.9, 24.8, 22.0; ESI-TOF-HRMS: m/z calcd for $[M-4Na+5H]^-$ $C_{58}H_{43}N_2O_{17}$, 1041.2713; found 1041.2728 (error = + 3.4 ppm).

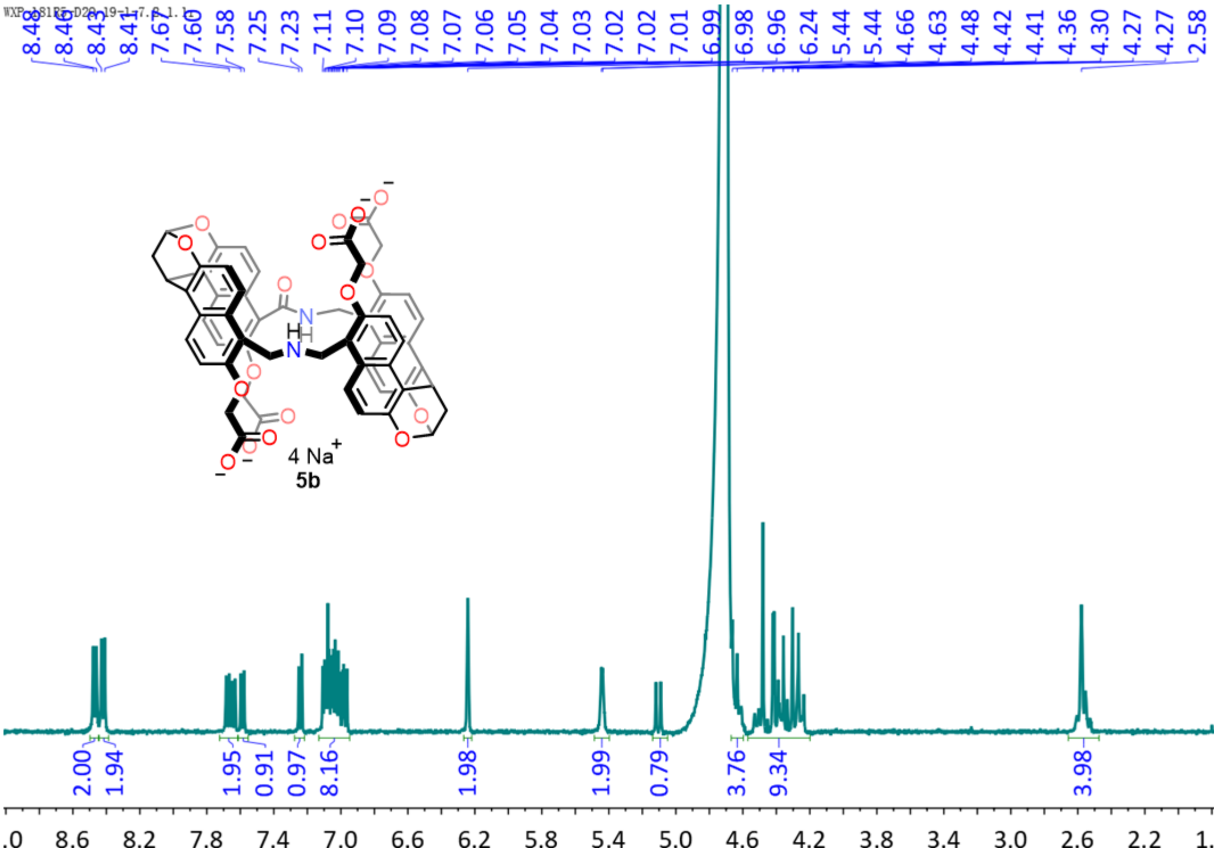

Supplementary Fig. 124 ^{1}H NMR spectrum (500 MHz, D_2O, 298 K) of compound **5b**

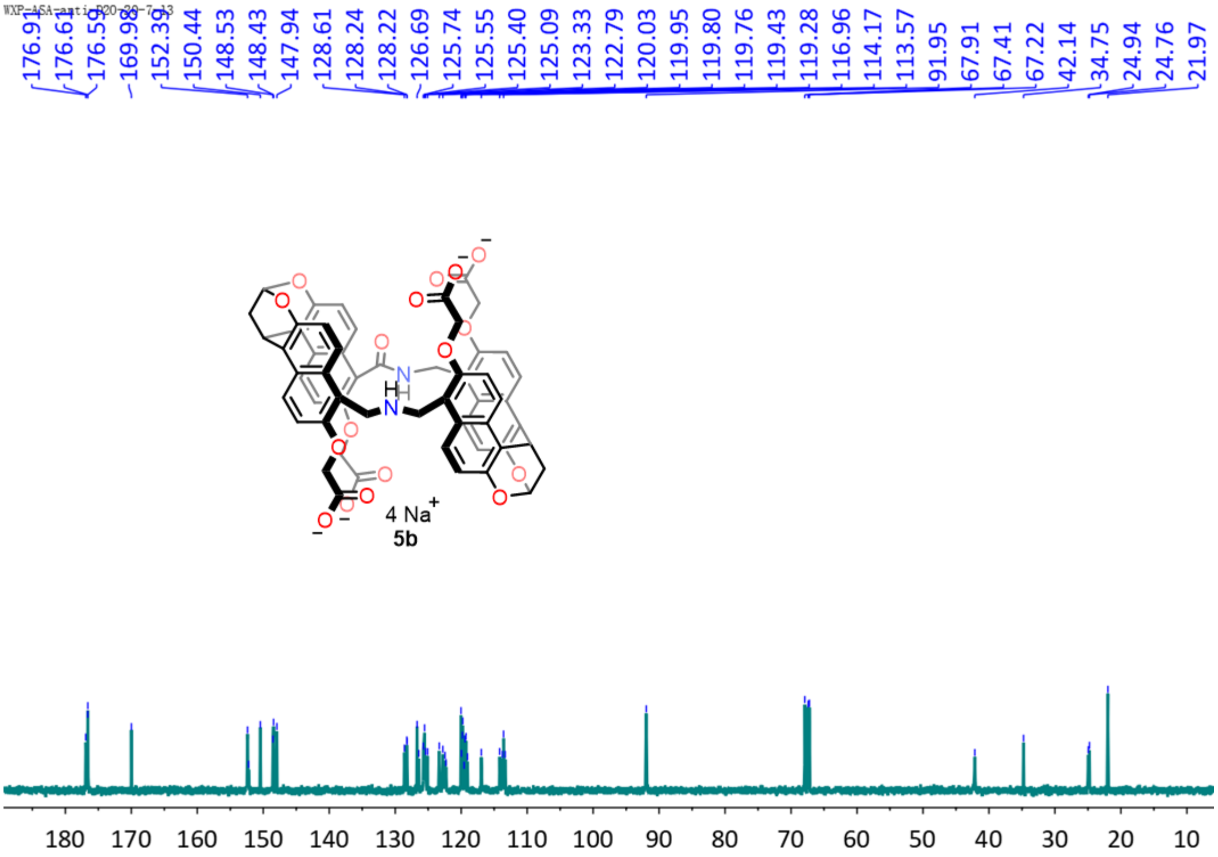

Supplementary Fig. 125 ^{13}C NMR spectrum (126 MHz, D_2O, 298 K) of compound **5b**

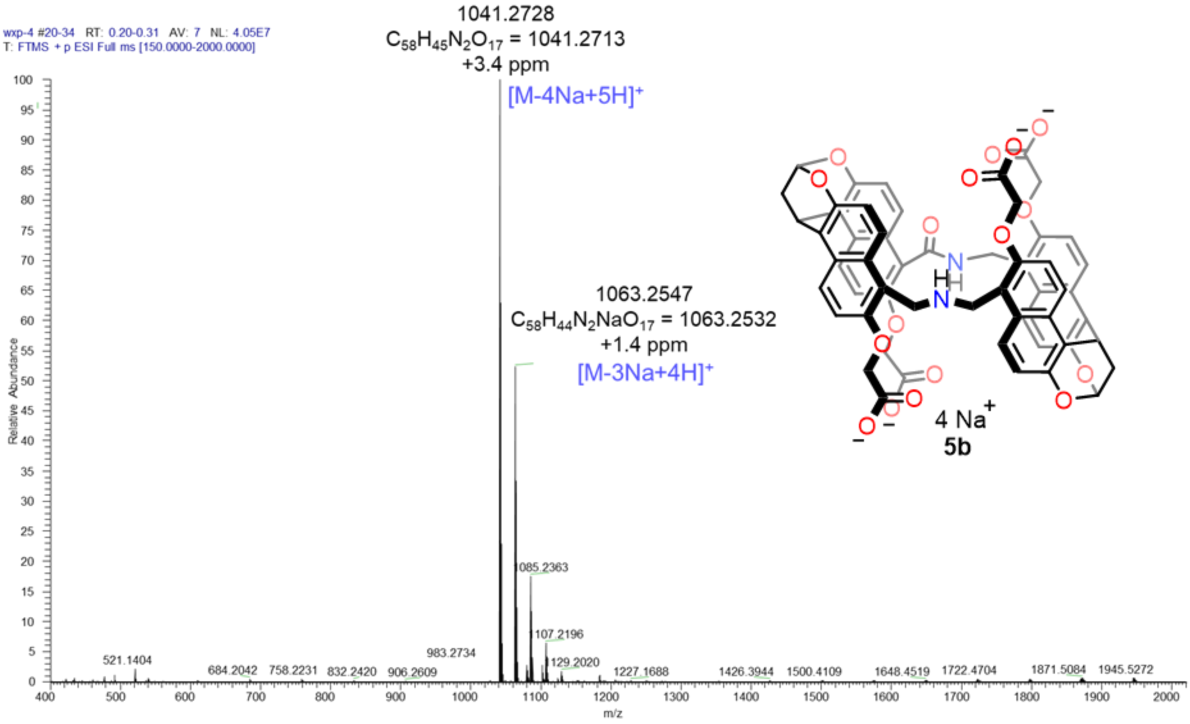

Supplementary Fig. 126 ESI mass spectrum of compound **5b**

13. Computational Data

All calculations were performed using Gaussian 09 package.[11] The host-guest complexes have been optimized employing wB97XD[12,13] functional. For carboxylic anions attached to the host molecular, 6-311+G(d) basis set was used. All other atoms were modeled at the 6-311G(d) level of theory. Geometry optimizations were performed by considering the solvent effects (PCM, water) without applying any geometry Constraints (C1 symmetry). Minima were characterized by the absence of imaginary frequencies.

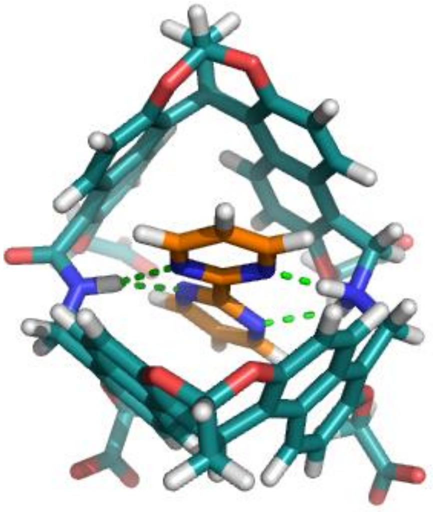

Supplementary Fig. 127 Energy-minimized structure of **G4@5a**·H$^+$. There is no imaginary frequency after optimization and frequency analysis.

Supplementary Table 6 Cartesian coordinates of **G4@5a**·H$^+$.

C	-23.13746500	8.92796300	0.23303300
C	-22.42340400	8.91345900	-0.98471800
C	-21.29935500	9.68036600	-1.17230700
C	-20.87019200	10.52657600	-0.09670400
C	-21.58677200	10.55408900	1.13483100
C	-22.72753100	9.71460900	1.26382600
C	-19.73761300	11.36439700	-0.20125000
C	-19.30306900	12.14085400	0.83763600
C	-20.00136100	12.14269800	2.06220900
C	-21.15282100	11.38957500	2.20026000
O	-22.95453800	8.07761300	-1.93210400
C	-20.56609600	9.56779900	-2.50885400
C	-22.22342100	7.88659900	-3.11927800
C	-21.58214500	9.17588500	-3.58021400
O	-21.27596500	6.85339500	-2.91880000
C	-19.98505800	7.17319500	-2.60164000
C	-19.13142700	6.06627100	-2.42711100

C	-17.83914000	6.25144200	-2.03495500
C	-17.30235400	7.55640200	-1.87705400
C	-18.13903800	8.66420400	-2.18605000
C	-19.52725600	8.46023200	-2.44899900
C	-15.97808700	7.78972700	-1.40430800
C	-15.48434300	9.07565800	-1.32924300
C	-16.23986700	10.15266300	-1.84322500
C	-17.52889100	9.94299400	-2.24165400
H	-24.00365700	8.28233400	0.32590000
H	-23.27058500	9.67146300	2.19921700
H	-19.18497200	11.41146400	-1.12505800
H	-18.42464500	12.75761500	0.69715000
H	-22.94008400	7.48305600	-3.83010200
H	-22.35829000	9.93449700	-3.69604100
H	-21.09624700	9.02585800	-4.54575400
H	-19.54003800	5.07181700	-2.56649000
H	-17.22737600	5.37612700	-1.85626300
H	-15.81800700	11.14659700	-1.91173800
H	-18.07637100	10.78371500	-2.64734900
C	-21.86517100	11.41747100	3.52524100
C	-15.18210300	6.70251600	-0.74504600
H	-22.93621700	11.23284700	3.41844000
H	-21.76421300	12.40437900	3.98100300
H	-20.11949100	10.52279500	-2.77856900
H	-21.08956600	2.01568000	5.96473100
C	-20.46152700	2.87677400	5.75079600
O	-21.33890200	3.88708500	5.27821000
O	-19.63122000	2.46654600	4.68997500
H	-22.99342500	5.69393200	4.66311200
H	-18.32122500	2.16541200	2.57775900
H	-20.24796700	3.50130300	7.80203000
H	-18.91381000	2.52429500	7.16473000
C	-19.62153500	3.32051700	6.92733900
C	-21.12465100	5.19853800	5.60819900
C	-22.13468300	6.09207900	5.19186700
C	-18.59239800	3.30112200	4.38001300
C	-17.96371000	3.01340100	3.15054400
C	-18.91795400	4.60434600	6.49043000
C	-20.00561600	5.63762500	6.27088300
H	-22.74088100	8.11642600	5.03585500
C	-21.99068300	7.42939100	5.41034700
C	-18.18099200	4.34487900	5.17563400
H	-18.21279000	4.90905300	7.26179000
C	-16.95612900	3.80297900	2.68983800
H	-16.51337500	3.56449300	1.73172700
C	-19.93111300	7.00903600	6.65956800
C	-20.88668900	7.92987300	6.15325400
C	-17.12269100	5.18347700	4.69671100
C	-16.51536800	4.93022600	3.43340300
H	-17.09792300	6.52755200	6.39450600

H	-18.27848900	6.82973800	8.05782100
C	-18.95607400	7.51094600	7.55664900
C	-16.67216800	6.30918800	5.42879900
C	-20.73272100	9.31554200	6.41510900
C	-15.51992800	5.82093800	2.94058600
C	-18.85474900	8.84422900	7.84769300
C	-19.72547500	9.77394200	7.23509300
C	-15.73166700	7.17058700	4.93670400
C	-15.16398800	6.93366400	3.67089600
H	-18.08584900	9.17613000	8.53350700
H	-15.45611900	8.04986200	5.50709400
C	-14.90580200	5.69515500	1.57517500
C	-21.60033300	10.31686700	5.69990100
N	-21.27123900	10.42485500	4.40466600
O	-14.29328400	9.22750600	-0.70200500
O	-19.59069000	12.83645400	3.15067100
O	-19.61848300	11.11547800	7.38508700
O	-14.35162200	7.84305200	3.06031300
C	-13.22995800	8.35862100	3.76536300
H	-13.49411400	8.61752800	4.79355900
C	-18.80909900	11.63322200	8.42836700
H	-19.16138200	12.65602300	8.57497000
H	-18.99085400	11.09003800	9.36090400
C	-13.67751500	10.50669100	-0.66453000
H	-12.86342400	10.40070000	0.05352000
C	-18.49147700	13.72211700	3.04285700
H	-14.11060500	6.85783100	-0.84904500
H	-12.96630500	9.28510100	3.25294300
H	-17.61896200	13.21404800	2.62268300
H	-20.49280200	9.87267300	4.05181500
H	-18.24428900	13.98209800	4.07386600
O	-22.49802400	10.95827000	6.23506300
H	-15.08441100	4.73727700	1.09526800
H	-13.83216700	5.87525000	1.63322800
C	-12.00183800	7.42310700	3.77159900
C	-17.29348000	11.69490700	8.14063600
C	-13.08980200	10.99848200	-2.00670200
C	-18.76352500	15.03019100	2.26720900
O	-17.73829300	15.72485700	2.07426500
O	-19.93740200	15.28809700	1.93611800
O	-12.63529800	12.16404800	-1.95768500
O	-13.10277100	10.21618600	-2.97639300
O	-16.89886000	11.44085900	6.98586500
O	-16.60621300	12.03386200	9.13236400
O	-12.06657500	6.35777700	3.12580400
O	-11.03757900	7.86421400	4.43805100
H	-15.41927600	5.70322600	-1.10018100
N	-15.50507300	6.75352100	0.71123400
H	-14.36985600	11.25582400	-0.26794100
H	-16.53818100	6.76790200	0.84717000

H	-15.19924800	7.65755000	1.07873100
N	-18.37296700	6.79302700	1.34201200
C	-20.50110700	5.73503600	1.29953700
N	-20.21637700	7.80444800	2.43551100
C	-20.99235100	6.80126600	2.03536400
C	-18.94241700	7.75764500	2.06813800
C	-19.15539000	5.77818600	0.97763900
H	-22.03725700	6.85819800	2.32031900
H	-21.13246400	4.91222900	0.99242200
H	-18.68438000	4.98122200	0.41423600
C	-18.06585800	8.87537600	2.53988200
C	-17.66337900	10.46651000	4.10659700
C	-16.17746100	10.09708100	2.31124200
C	-16.48032800	10.80159300	3.46265800
N	-16.95898300	9.12217600	1.84662600
N	-18.46456200	9.51117700	3.63840600
H	-17.95929300	10.95607300	5.02960900
H	-15.82591200	11.57314900	3.84603100
H	-15.27360700	10.30105900	1.74621500

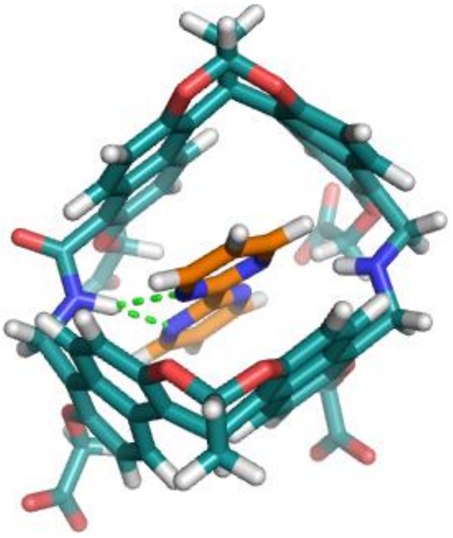

Supplementary Fig. 128 Energy-minimized structure of **G4@5a**. There is no imaginary frequency after optimization and frequency analysis.

Supplementary Table 7 Cartesian coordinates of **G4@5a**.

C	-24.02152700	8.16850600	0.31552100
C	-23.37603700	7.92178500	-0.91385800
C	-22.34011400	8.71142000	-1.34662400
C	-22.00046100	9.87190400	-0.58834700
C	-22.54836900	10.05408600	0.70920700
C	-23.57532200	9.16676300	1.13222200
C	-21.13501400	10.88214700	-1.07585600
C	-20.73855500	11.93013000	-0.29390400
C	-21.14844900	12.00559200	1.05799400

C	-22.05792800	11.09337500	1.55251400
O	-23.83675100	6.83228400	-1.60299900
C	-21.62263200	8.25473900	-2.60239900
C	-23.33422200	6.54911200	-2.89915400
C	-22.70393200	7.75413800	-3.56042200
O	-22.41094400	5.49053900	-2.81463900
C	-21.12043200	5.81064400	-2.47843900
C	-20.27261200	4.69258900	-2.31453600
C	-18.95869200	4.86130400	-2.00401000
C	-18.41553800	6.16273800	-1.81534500
C	-19.28960600	7.27977600	-1.94615400
C	-20.66148600	7.09606100	-2.32325900
C	-17.04480500	6.34568600	-1.47894400
C	-16.57758600	7.61596900	-1.19369600
C	-17.44030100	8.72639600	-1.30948200
C	-18.74508000	8.55622700	-1.68348800
H	-24.82818400	7.51032900	0.61916700
H	-24.03386300	9.28720100	2.10591100
H	-20.80017200	10.86045600	-2.10604500
H	-20.09229400	12.68638200	-0.71991800
H	-24.17692100	6.14207100	-3.45263700
H	-23.45520200	8.52517300	-3.73905400
H	-22.28720700	7.44526100	-4.52089600
H	-20.70103400	3.70446000	-2.44193300
H	-18.33023100	3.99295200	-1.85310400
H	-17.08341600	9.72868300	-1.10930900
H	-19.36698000	9.43453100	-1.75279200
C	-22.33964600	11.04348100	3.03189200
C	-16.12489100	5.16281100	-1.29303800
H	-23.39210200	10.86395600	3.25835000
H	-22.06604500	11.98587700	3.50785600
H	-21.07926400	9.07249700	-3.07331400
H	-22.15605900	0.91308400	4.59965000
C	-21.50896300	1.77169800	4.43845300
O	-22.38652500	2.85441700	4.23418200
O	-20.82241900	1.49805600	3.23157500
H	-23.82570200	4.84800100	3.81261300
H	-19.52238000	0.97112500	1.15146100
H	-21.15521600	2.26219200	6.48340300
H	-19.96044000	1.15380800	5.78738300
C	-20.56778900	2.03889600	5.59084400
C	-21.90046600	4.12002100	4.42830300
C	-22.84132500	5.13986900	4.16122700
C	-19.55744400	1.98390800	3.04686700
C	-18.96080800	1.60774300	1.82577900
C	-19.69609700	3.22460300	5.17606700
C	-20.62801700	4.39928800	4.86602600
H	-23.24691700	7.21937400	4.13936000
C	-22.51320800	6.44756500	4.34025600
C	-18.90321900	2.78255200	3.95557900

H	-19.03697900	3.48314900	6.00290900
C	-17.73226600	2.09461200	1.49085800
H	-17.31902300	1.86762000	0.51670400
C	-20.27236100	5.77579000	5.05839900
C	-21.21980300	6.80973200	4.80573000
C	-17.55322500	3.16142300	3.67853400
C	-16.99856200	2.90702600	2.39478900
H	-17.03301900	3.87818000	5.66557600
H	-18.24766400	5.43016900	5.73752700
C	-18.99969000	6.17267600	5.52569500
C	-16.71929800	3.80553000	4.63194600
C	-20.86070300	8.16923000	4.99397500
C	-15.76557500	3.52068800	2.00900000
C	-18.66896700	7.48556000	5.72825800
C	-19.60631600	8.50628700	5.46214000
C	-15.49456500	4.30049500	4.28634100
C	-15.04468100	4.21859800	2.94827600
H	-17.68115400	7.72404100	6.10067800
H	-14.85686800	4.78948900	5.01400500
C	-15.42663400	3.65206900	0.54498400
C	-21.84737900	9.26968000	4.68214900
N	-21.54566500	9.94909900	3.57112800
O	-15.28869200	7.72496600	-0.78193000
O	-20.65644100	12.92839200	1.92399000
O	-19.35176200	9.82200900	5.64767700
O	-13.87573700	4.83037400	2.58398000
C	-13.87879900	6.25571000	2.63057000
H	-14.89936300	6.63869800	2.51808000
C	-18.05367000	10.22398700	6.04374500
H	-17.85127100	9.89798500	7.07009000
H	-17.29811700	9.77475000	5.39175500
C	-14.74948400	9.00256800	-0.50401800
H	-13.81728200	8.80281300	0.02817400
C	-19.73605200	13.89883600	1.45793000
H	-15.08389400	5.50772100	-1.32375900
H	-13.31003500	6.58889700	1.75838800
H	-18.89243400	13.42257300	0.94760600
H	-20.73373600	9.66283700	3.02846400
H	-19.34349300	14.36609800	2.36297200
O	-22.83484600	9.49009100	5.38110400
H	-15.42755000	2.68597500	0.02706100
H	-14.41819800	4.06989700	0.44000000
C	-13.23833600	6.88645700	3.88319500
C	-17.88595400	11.75284900	5.96470300
C	-14.41976700	9.87353100	-1.73662100
C	-20.32830000	15.01280400	0.56458600
O	-19.46395200	15.76586000	0.05833500
O	-21.56762200	15.08302000	0.44865700
O	-14.04774100	11.03590400	-1.44898600
O	-14.52285500	9.36311200	-2.86959600

O	-16.75673300	12.14302600	6.34840900
O	-18.82714300	12.44782400	5.53879800
O	-12.81635400	6.14669900	4.79453900
O	-13.20180600	8.14171400	3.84312600
H	-16.24150100	4.44199600	-2.11118500
N	-16.45293800	4.52061200	-0.02691900
H	-15.39380800	9.56116700	0.18024200
H	-16.68663800	5.24276800	0.64788800
N	-18.43272700	6.22178100	1.68284700
C	-20.61741100	5.39756000	1.22074200
N	-20.29314200	7.68585400	1.76102200
C	-21.09626600	6.67485400	1.44518400
C	-18.99817000	7.40947400	1.87410000
C	-19.25129100	5.22663500	1.34355400
H	-22.15200300	6.90452400	1.37720600
H	-21.27192100	4.57720800	0.95671400
H	-18.78359700	4.27164800	1.14661500
C	-18.10996800	8.55747000	2.25316300
C	-17.92306100	10.78207300	2.66788500
C	-16.04956100	9.35267200	2.77161800
C	-16.55921000	10.63938800	2.87037700
N	-16.81984600	8.30796600	2.46200400
N	-18.69983800	9.74823100	2.35890900
H	-18.41962000	11.74051700	2.77660500
H	-15.93027900	11.48277900	3.12372400
H	-14.99994000	9.13352300	2.96139200

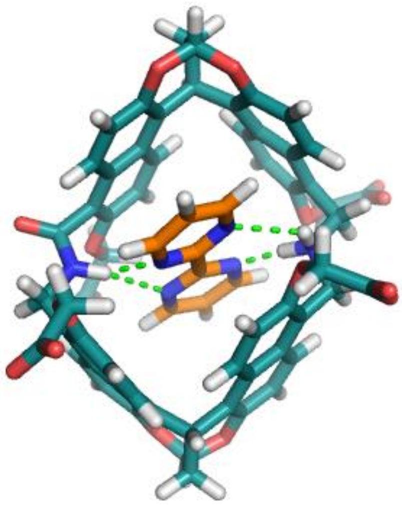

Supplementary Fig. 129 Energy-minimized structure of **G4@5b·H**$^{+}$. There is no imaginary frequency after optimization and frequency analysis.

Supplementary Table 8 Cartesian coordinates of **G4@5b·H**$^{+}$.

C	-9.97084800	2.93637900	-1.74538500

C	-10.58795300	1.67280300	-1.63668900
C	-9.86378300	0.53302200	-1.37365000
C	-8.45137300	0.66453200	-1.16993900
C	-7.82235900	1.94122700	-1.25350100
C	-8.63029200	3.06960800	-1.55793600
C	-7.63630400	-0.45005000	-0.85469000
C	-6.29912500	-0.32689300	-0.59933000
C	-5.68840600	0.93828500	-0.67252000
C	-6.42234400	2.05318500	-1.01374200
O	-11.94390800	1.69637900	-1.81129000
C	-10.60829400	-0.79979000	-1.26208000
C	-12.64211100	0.52089100	-1.47076400
C	-11.92730600	-0.69104200	-2.02516000
O	-12.80460500	0.47704900	-0.06412200
C	-12.01079500	-0.34100500	0.69662800
C	-12.30853600	-0.34845100	2.07422600
C	-11.53570400	-1.07044100	2.93350000
C	-10.50000900	-1.91744600	2.45295700
C	-10.26702800	-1.96557400	1.05201900
C	-10.96642900	-1.07149100	0.18671400
C	-9.68177400	-2.67580400	3.33836200
C	-8.71393100	-3.51824900	2.83119900
C	-8.59562100	-3.69186200	1.43245500
C	-9.34279600	-2.92911100	0.57915900
H	-10.59643800	3.79478900	-1.96170400
H	-8.19649400	4.05836000	-1.62914200
H	-8.07488900	-1.43135300	-0.77959200
H	-5.72301000	-1.19843400	-0.31108700
H	-13.63963400	0.66088100	-1.87945100
H	-11.77184100	-0.54037000	-3.09498400
H	-12.53019800	-1.58927900	-1.88427000
H	-13.10818600	0.28140200	2.44738900
H	-11.72624000	-0.97334000	3.99412100
H	-7.91005300	-4.42118200	1.02108600
H	-9.23484700	-3.10149200	-0.48528700
C	-9.75560600	-2.41488600	4.81818000
C	-5.68142500	3.36086300	-1.02182100
H	-9.20176800	-3.17234700	5.37255400
H	-10.78572800	-2.44018300	5.18626200
H	-10.01013700	-1.60081800	-1.69336600
H	-1.12962400	1.85763100	7.40337200
C	-2.08191900	2.06463000	6.92238600
O	-2.83711100	0.88821200	7.08580700
O	-1.77616700	2.23236400	5.54821900
H	-4.25609500	-1.16818800	7.06747900
H	-1.26234100	2.59996400	3.11115100
H	-3.03761100	3.05078700	8.55118800
H	-2.16485000	4.14133700	7.46113400
C	-2.80401400	3.25799900	7.50543400
C	-4.18983900	0.97241500	6.88640300

C	-4.85101900	-0.27672000	6.90452100
C	-2.53559200	3.06854200	4.77969100
C	-2.13518200	3.15730800	3.43160100
C	-4.06732500	3.45359200	6.66991400
C	-4.86803100	2.15142500	6.69014900
H	-6.68569200	-1.31504700	6.71242300
C	-6.19379000	-0.34895400	6.70382300
C	-3.62282200	3.76587700	5.25192900
H	-4.65314900	4.26505300	7.09743400
C	-2.87902100	3.88176200	2.54664200
H	-2.57989600	3.89080300	1.50475800
C	-6.28700400	2.08878700	6.48923500
C	-6.95662300	0.83237200	6.48924600
C	-4.29585900	4.66205000	4.37054900
C	-3.99550500	4.64172700	2.98307900
H	-5.42446800	5.74867300	5.87612200
H	-6.63597000	4.21802700	6.33110900
C	-7.08538000	3.23894100	6.30066600
C	-5.27014700	5.59411200	4.81592000
C	-8.35479700	0.77239900	6.27043300
C	-4.82612500	5.35876300	2.07073000
C	-8.43745300	3.16977700	6.08760400
C	-9.09030300	1.91950100	6.05864200
C	-6.01154500	6.33009500	3.93819200
C	-5.84001600	6.15678400	2.54577100
H	-8.99132400	4.08994300	5.95438400
H	-6.76982700	7.01047600	4.30792300
C	-4.75761700	5.07571400	0.60057900
C	-9.05839300	-0.56130000	6.27527700
N	-9.17545800	-1.10199100	5.05857400
O	-7.88836400	-4.12673700	3.72201000
O	-4.39346500	1.13611300	-0.28942700
O	-10.41486400	1.76109100	5.83540700
O	-6.73745600	6.69255100	1.66872100
C	-6.89299800	8.10794600	1.66780900
H	-6.98020300	8.49542200	2.68680700
C	-11.22952900	2.90900200	5.67974900
H	-11.26498500	3.47585700	6.61623500
H	-10.82314300	3.56800900	4.90499300
C	-6.87300400	-4.99329800	3.24747400
H	-6.22360900	-5.15821900	4.10898000
H	-6.27176900	-4.50032800	2.47662500
C	-3.37389900	0.32152600	-0.85511000
H	-6.24702000	4.18716900	-1.44253800
H	-7.84286400	8.29572900	1.16464700
H	-3.68196500	-0.72618500	-0.89152500
H	-8.79207100	-0.59974900	4.26097600
H	-2.53136100	0.39088900	-0.16524200
O	-9.45273200	-1.09800800	7.30667200
H	-3.74953100	5.07534700	0.18760700

H	-5.35026400	5.80293000	0.05463200
C	-5.77980000	8.87881900	0.92614500
C	-12.66532200	2.53665100	5.26134000
C	-7.35401800	-6.37230300	2.74170200
C	-2.90883800	0.76744500	-2.25824000
O	-3.37901300	1.82165000	-2.73146100
O	-2.06562800	-0.00079100	-2.77519200
O	-8.54571600	-6.68650600	2.92823100
O	-6.45107500	-7.05504800	2.20384100
O	-12.93551900	1.34877800	5.00717400
O	-13.43015000	3.52988100	5.20125300
O	-5.88201400	10.12452500	1.02263600
O	-4.92048900	8.22570000	0.30195800
H	-4.74470500	3.24615900	-1.56904800
N	-5.34878900	3.72021100	0.38664400
H	-6.19590700	3.61205600	0.98553200
H	-4.72179800	2.99671500	0.74615300
N	-5.62148400	1.54823500	2.51343700
C	-4.82759800	-0.59888600	3.17036400
N	-7.15798600	-0.15664000	3.09389300
C	-6.14797400	-0.98548600	3.33627900
C	-6.84982500	1.07338300	2.69275000
C	-4.61782000	0.70374200	2.75047900
H	-6.41595300	-1.98085400	3.68044800
H	-4.00568700	-1.27364300	3.36848400
H	-3.61773400	1.09373000	2.59820300
C	-8.00281300	1.99674200	2.43158500
C	-8.76932300	4.01550500	1.71445400
C	-10.23681500	2.35749700	2.51806800
C	-10.06833600	3.63480200	2.00676800
N	-9.21014000	1.54067400	2.73830700
N	-7.73548800	3.19946700	1.91758600
H	-8.53382000	4.99454100	1.30928400
H	-10.90650500	4.29734000	1.83714900
H	-11.21796600	1.96679700	2.76948500

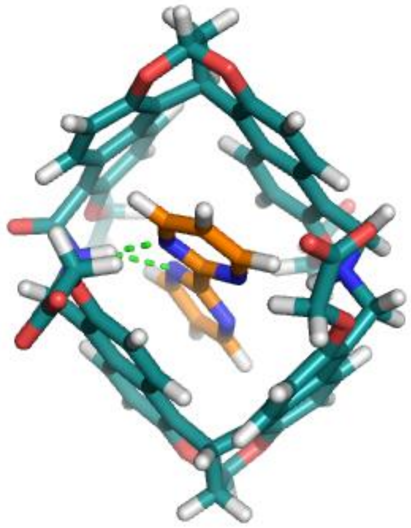

Supplementary Fig. 130 Energy-minimized structure of **G4@5b**. There is no imaginary frequency after optimization and frequency analysis.

Supplementary Table 9 Cartesian coordinates of **G4@5b**.

C	-9.30319400	3.22941900	-2.05095500
C	-10.03508100	2.03222200	-1.92439600
C	-9.40999700	0.81327900	-1.85752100
C	-7.99858100	0.75655600	-2.06973800
C	-7.23692100	1.95943800	-2.11373900
C	-7.94116300	3.19524500	-2.09072800
C	-7.30176100	-0.46389200	-2.24402700
C	-5.93719300	-0.50503000	-2.31834500
C	-5.18022300	0.68166600	-2.18994400
C	-5.81056300	1.90693700	-2.13048700
O	-11.39248900	2.19253800	-1.83207000
C	-10.29533200	-0.37585600	-1.52807300
C	-12.24432100	1.05725800	-1.80722600
C	-11.57782200	-0.19208700	-2.33972600
O	-12.69123300	0.85370400	-0.49057600
C	-11.83153400	0.20276000	0.35669300
C	-12.23821500	0.22022400	1.70783600
C	-11.47584500	-0.36665600	2.66810500
C	-10.25908600	-1.02328900	2.33317800
C	-9.85131600	-1.01969100	0.96609000
C	-10.66498400	-0.40244200	-0.04170800
C	-9.45716200	-1.65262100	3.32654300
C	-8.24427400	-2.21427000	2.97209900
C	-7.83160500	-2.20997900	1.62400000
C	-8.61770900	-1.63670300	0.66200400
H	-9.84281900	4.16991000	-2.07316200
H	-7.40024700	4.13238100	-2.12277200
H	-7.84781600	-1.39307500	-2.35374100
H	-5.44016300	-1.45723800	-2.45850500
H	-13.13217200	1.34629100	-2.36440900

H	-11.35426600	-0.08108000	-3.40190800
H	-12.26703800	-1.02940100	-2.21500100
H	-13.15721800	0.73835200	1.95762300
H	-11.79484300	-0.29986400	3.70050900
H	-6.89180500	-2.65996300	1.33072200
H	-8.25991200	-1.65663600	-0.35559600
C	-9.84457600	-1.65141900	4.78469600
C	-5.00479800	3.14155700	-1.80666000
H	-9.46511100	-2.55465100	5.26719500
H	-10.92822900	-1.65912800	4.91881400
H	-9.82773000	-1.31799400	-1.80890400
H	-1.16778300	1.13633200	7.09541900
C	-2.08095200	1.46491000	6.60554000
O	-2.97983200	0.37145500	6.71473100
O	-1.72601500	1.63801400	5.25509300
H	-4.60862700	-1.52320100	7.09846000
H	-1.34784700	1.85185800	2.78895600
H	-2.80121800	2.64460000	8.25246900
H	-1.92483300	3.54937700	7.00517600
C	-2.64433100	2.74681300	7.17749200
C	-4.31920800	0.59966300	6.87629000
C	-5.10457000	-0.55958200	7.06514200
C	-2.58309000	2.36658100	4.47085700
C	-2.22890300	2.40052700	3.10348300
C	-3.96699700	2.99585300	6.45329400
C	-4.88547900	1.84923100	6.82816000
H	-7.06689600	-1.35640500	7.18883400
C	-6.46375300	-0.45771400	7.11873900
C	-3.69086800	3.02379200	4.94765700
H	-4.37538500	3.95518900	6.76812000
C	-2.98326100	3.09790700	2.21125200
H	-2.71220800	3.08370100	1.16358500
C	-6.28296100	1.97828900	7.08882400
C	-7.09576700	0.81600100	7.11631800
C	-4.51990700	3.71819700	4.00472700
C	-4.15584200	3.78758100	2.62885600
H	-6.05146000	4.31832400	5.41210700
H	-6.32829800	4.11678900	7.46460900
C	-6.92076400	3.21879000	7.33454200
C	-5.73015400	4.33988200	4.38276800
C	-8.50905400	0.94674400	7.12950100
C	-4.97153400	4.48487600	1.69408900
C	-8.28029500	3.32205500	7.45858300
C	-9.09812700	2.18139000	7.27825500
C	-6.54619600	4.95297700	3.47172800
C	-6.18005700	5.01261100	2.11053900
H	-8.71761500	4.29075900	7.66168700
H	-7.47350600	5.39225300	3.81653500
C	-4.62224600	4.51970500	0.22470000
C	-9.34121000	-0.25457300	6.75281600

N	-9.27122900	-0.48238200	5.43306900
O	-7.48404200	-2.70932400	3.97876800
O	-3.82251800	0.66380500	-2.12399100
O	-10.44981200	2.23035000	7.20709000
O	-6.98029500	5.55223900	1.15542500
C	-8.27790800	5.99259000	1.50682800
H	-8.79978700	5.23217500	2.09443300
C	-11.10461900	3.47125500	7.40154400
H	-10.93981800	3.83256500	8.42174200
H	-10.71061400	4.22662800	6.71099700
C	-6.27590900	-3.38295600	3.67730900
H	-5.77919100	-3.50440000	4.64180600
H	-5.62551800	-2.76000500	3.05616500
C	-3.19223700	-0.49665400	-1.60913800
H	-5.42721200	4.03723800	-2.28728300
H	-8.80908900	6.08359700	0.55704900
H	-3.09988400	-1.26270300	-2.38644400
H	-8.67023200	0.12256900	4.87641600
H	-3.79000500	-0.92024200	-0.79371100
O	-9.97181000	-0.94826000	7.54415200
H	-3.55047700	4.69528800	0.07940100
H	-5.14440500	5.35872700	-0.25503500
C	-8.35677000	7.35385900	2.23215100
C	-12.62032500	3.35460300	7.14832800
C	-6.43313900	-4.78077500	3.03731600
C	-1.78692700	-0.19441200	-1.05168800
O	-1.38844300	0.98491800	-1.00626200
O	-1.17828900	-1.22402000	-0.67259900
O	-7.57408500	-5.27960300	2.97875900
O	-5.35085900	-5.28702300	2.65844100
O	-13.07547400	2.29573700	6.67613700
O	-13.24984600	4.39937200	7.43920100
O	-9.50915000	7.64433800	2.63228900
O	-7.31761100	8.03552200	2.33441800
H	-3.98355600	3.04079300	-2.17902100
N	-4.98830500	3.23727700	-0.35345200
H	-5.88810300	2.95404100	0.02094900
N	-6.58303500	1.47825800	1.52343800
C	-5.00057700	0.11657800	2.65999600
N	-7.05499700	0.69390700	3.70503800
C	-5.88248500	0.06534300	3.72441900
C	-7.35333500	1.37350000	2.60062300
C	-5.40686400	0.85524700	1.56206200
H	-5.65879100	-0.50131500	4.61942600
H	-4.04079900	-0.38223800	2.68965800
H	-4.77226400	0.99210000	0.69256700
C	-8.68035800	2.07372500	2.58664900
C	-10.35701600	3.04027600	1.41642400
C	-10.44935400	2.86039200	3.76169900
C	-11.05024000	3.29128200	2.59014000

N	-9.26517900	2.25517800	3.76952500
N	-9.17036300	2.43935800	1.40579900
H	-10.76743700	3.31720600	0.44990600
H	-12.01720200	3.77703600	2.58940000
H	-10.93338700	2.98239000	4.72543600

14. Supplementary References

[1] Huang, X. et al. Biomimetic recognition and optical sensing of carboxylic acids in water by using a buried salt bridge and the hydrophobic effect. *Angew. Chem. Int. Ed.* **60**, 1929-1935 (2021).

[2] Ke, H. et al. Shear-induced assembly of a transient yet highly stretchable hydrogel based on pseudopolyrotaxanes. *Nat. Chem.* **11**, 470-477 (2019).

[3] Chai, H. et al. Enantioselective recognition of neutral molecules in water by a pair of chiral biomimetic macrocyclic receptors. *CCS Chem.* **2**, 440-452 (2020).

[4] http://ibond.nankai.edu.cn/.

[5] Calculated using Advanced Chemistry Development (ACD/Labs) Software V11.02 (© 1994-2021 ACD/Labs)

[6] The pK_a values were obtained by predicting based on highly similar structures provided on http://ibond.nankai.edu.cn/.

[7] Sheldrick, G. M. SHELXT-integrated space-group and crystal-structure determination. *Acta Cryst.* **A71**, 3–8 (2015).

[8] Sheldrick, G. M. Crystal structure refinement with SHELXL. *Acta Cryst.* **C71**, 3–8 (2015).

[9] Dolomanov, O. V., Bourhis, L. J., Gildea, R. J., Howard, J. A. K. & Puschmann, H. OLEX2: A complete structure solution, refinement and analysis program *J. Appl. Cryst.* **42**, 339–341 (2009).

[10] Spek, A.L. A tool for the calculation of the disordered solvent contribution to the calculated structure factors. *Acta Crystallorgr.* **C71**, 9-18 (2015).

[11] *Gaussian 09, Revision D.01,* Frisch, M. J., Trucks, G. W., Schlegel, H. B., Scuseria, G. E., Robb, M. A., Cheeseman, J. R., Scalmani, G., Barone, V., Mennucci, B., G. Petersson, A., Nakatsuji, H., Caricato, M., Li, X.; H. Hratchian, P., Izmaylov, A. F., Bloino, J., Zheng, G., Sonnenberg, J. L., Hada, M., Ehara, M., Toyota, K., Fukuda, R., Hasegawa, J., Ishida, M., Nakajima, T., Honda, Y., Kitao, O., Nakai, H., Vreven, T., Montgomery, J. A., Peralta, J. E., Ogliaro, F., Bearpark, M., Heyd, J. J., Brothers, E., Kudin, K. N., Staroverov, V. N., Kobayashi, R., Normand, J., Raghavachari, K.,Rendell, A.; Burant, J. C., Iyengar, S. S., Tomasi, J., Cossi, M., Rega, N., Millam, J. M., Klene, M.; Knox, J. E., Cross, J. B., Bakken, V., Adamo, C., Jaramillo, J., Gomperts, R., Stratmann, R. E., Yazyev, O., Austin, A. J., Cammi, R., Pomelli, C., Ochterski, J. W., Martin, R. L., Morokuma, K., Zakrzewski, V. G., Voth, G. A., Salvador, P., Dannenberg, J. J., Dapprich, S., Daniels, A. D., Farkas, O., Foresman,J. B., Ortiz, J. V., Cioslowski, J. & Fox, D. J., Gaussian, Inc.: Wallingford CT, USA, 2013.

[12] Chai, J.-D. & Head-Gordon, M. Long-range corrected hybrid density functionals with damped atom–atom dispersion corrections. *Phys. Chem. Chem. Phys.* **10**, 6615–6620 (2008).

[13] Marenich, A. V., Cramer, C. J. & Truhlar, D. G. Universal solvation model based on solute electron density and on a continuum model of the solvent defined by the bulk dielectric constant and atomic surface tensions. *J. Phys. Chem. B.* **113**, 6378–6396 (2009).
